# CCD-Optimized UA-DMSPE Combined with DFT Assisted Selection of a Novel Anthraquinone-Based Sorbent for Pb(II) and Cd(II) Determination in Rice and Paddy Soil

**DOI:** 10.1021/acsomega.6c05694

**Published:** 2026-08-26

**Authors:** Taşkın Mumcu, Serkan Öncüoğlu, Akın Mumcu, Ülkü Yılmaz

**Affiliations:** † Department of Chemistry, Faculty of Sciences, 37508 Dokuz Eylul University, Merkez Campus, 35160 Buca-Izmir, Turkey; ‡ Department of Chemistry, Faculty of Sciences, Dokuz Eylul University, Merkez Campus, 35160 Buca-Izmir, Turkey; § 37520 Inonu University, Scientific and Technological Research Center, Laboratory of NMR, Malatya 44280, Turkey; ∥ 531771 Malatya Turgut Özal University, Faculty of Engineering and Natural Sciences, Department of Engineering Basic Sciences, Malatya 44900, Turkey

## Abstract

In this study, a novel ultrasound-assisted dispersive micro solid-phase extraction (UA-DMSPE) method coupled with flame atomic absorption spectrometry (FAAS) was developed for the rapid, reliable, low-cost, and environmentally friendly determination of Pb­(II) and Cd­(II) ions in rice and paddy soil samples. Three novel anthraquinone-derived ligands, not previously reported in the literature, were synthesized and their metal-binding behaviors were evaluated through density functional theory (DFT) calculations. Based on Frontier molecular orbital (FMO), molecular electrostatic potential (MEP), and global reactivity descriptor analyses, Derivative 2, exhibiting the lowest HOMO–LUMO energy gap (Δ*E* = 2.1284 eV) together with the highest softness (σ = 347.8739) and electrophilicity index (ω = 28.2323), was identified as the most promising ligand for Pb­(II) and Cd­(II) coordination. Accordingly, Derivative 2 was selected and loaded onto silica to prepare a novel anthraquinone-functionalized sorbent and employed throughout all experimental studies. The principal parameters affecting extraction efficiency were optimized using a six-factor central composite design (CCD) coupled with response surface methodology (RSM), enabling statistical evaluation of the relationships between experimental variables and extraction recovery. Under optimized conditions, the developed method exhibited low limits of detection of 0.19 and 0.28 μg L^–1^ for Pb­(II) and Cd­(II), respectively, wide linear working ranges (0.8–500 and 1.0–500 μg L^–1^), and satisfactory precision (%RSD < 2.2). The proposed method demonstrated high tolerance toward coexisting ions and satisfactory recovery values in rice and paddy soil samples. Moreover, the simultaneous evaluation of rice and paddy soil samples enabled a reliable assessment of the potential transfer of heavy metals from soil to plant systems. The environmental performance of the proposed method was assessed using Analytical Eco-Scale, GAPI, and AGREE metrics, demonstrating high environmental compatibility owing to low reagent consumption, solvent-free operation, and minimal waste generation (AGREE score ≈ 0.84). Overall, the developed method combines DFT-assisted sorbent selection, CCD-based optimization, high selectivity, low detection limits, and strong green analytical performance, providing an effective and reliable analytical platform for the determination of Pb­(II) and Cd­(II) in rice and paddy soil samples.

## Graphical abstract

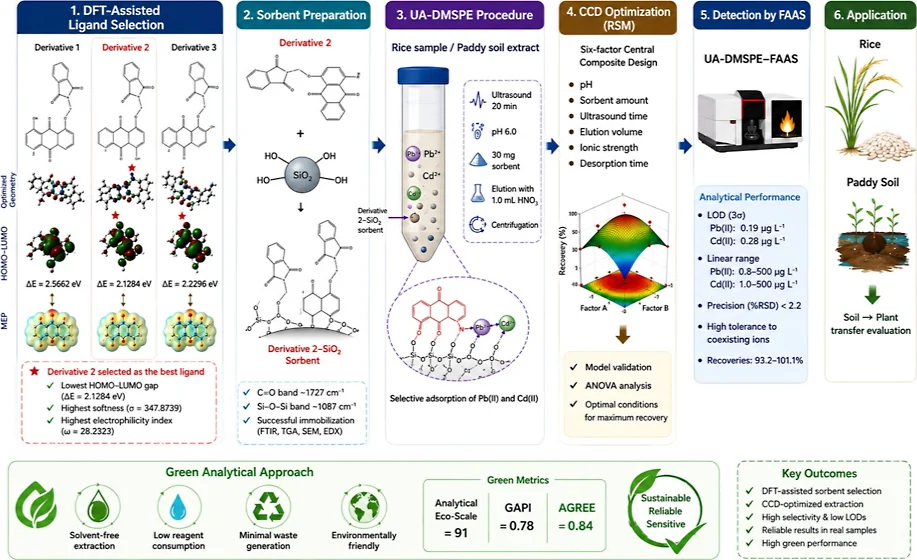

## Introduction

Heavy metal pollution is regarded as one of the major environmental concerns worldwide due to the persistence of toxic elements in the environment, their nonbiodegradable nature, and their tendency to undergo bioaccumulation through transport along the food chain.^1^ Heavy metals, which are silent poisons, are becoming a threat to the environment and human health in daily life. Recently, there has been a shift toward using phytochemicals or similar synthetic molecules to remove heavy metals from the environment.^2^ Among these contaminants, lead (Pb) and cadmium (Cd) are of particular concern owing to their high toxicity, bioaccumulation potential, and their ability to induce severe physiological disorders even at trace levels.^3^ These metals are introduced into the environment through various anthropogenic sources, including industrial activities, mining operations, phosphate-based fertilizer applications, wastewater discharge, and atmospheric deposition, eventually accumulating particularly in agricultural areas.^4^ Paddy soils used for rice cultivation are of particular importance in terms of heavy metal mobility, solubility, and bioavailability due to their continuously flooded conditions. Under anaerobic environments, the chemical behavior of Pb­(II) and Cd­(II) ions may change significantly, thereby influencing the uptake of these metals by rice plants.^5^ Under flooded paddy conditions, changes in soil redox potential may substantially alter the mobility and speciation of heavy metals, thereby affecting their transfer from soil to rice grains.^6^ In particular, Cd­(II) and Pb­(II) can be absorbed through rice root systems and subsequently translocated to edible tissues, representing an important pathway for human dietary exposure.^7^ Since the transfer of toxic metals from soil to rice grains constitutes a critical pathway for human exposure, the simultaneous evaluation of both paddy soil and rice samples is of considerable importance for understanding metal transport, bioavailability, and food-chain contamination. Given that rice serves as a staple food for a substantial proportion of the global population, the accumulation of toxic metals in rice poses serious risks to human health. Therefore, the accurate, sensitive, and selective determination of Pb­(II) and Cd­(II) ions in rice and paddy soil samples is of great significance for environmental monitoring and food safety applications.^8^ The presence of heavy metal ions at very low concentrations in environmental samples may limit the sensitivity of direct instrumental determination methods.^9^ In addition, complex sample matrices may cause various interference effects during analysis. Therefore, the implementation of appropriate separation and preconcentration procedures prior to instrumental analysis often becomes essential.^10^ Conventional techniques, such as liquid–liquid extraction (LLE), solid-phase extraction (SPE), cloud point extraction (CPE), and coprecipitation, have been widely employed; however, these methods are often associated with several drawbacks, including high solvent consumption, lengthy extraction procedures, and multistep operational processes.^11^ In recent years, dispersive micro solid-phase extraction (DMSPE) has emerged as an attractive alternative owing to its low sorbent consumption, short extraction time, high enrichment factor, and environmentally friendly characteristics.^12^ Furthermore, ultrasound-assisted dispersive micro solid-phase extraction (UA-DMSPE) significantly enhances extraction efficiency by increasing sorbent–analyte interactions and accelerating mass transfer through ultrasonic energy.^13^ Moreover, coupling UA-DMSPE with flame atomic absorption spectrometry (FAAS) provides a cost-effective and accessible analytical platform by compensating for the relatively lower sensitivity of FAAS through efficient preconcentration, thereby enabling reliable determination of trace metal ions in complex environmental matrices. In recent years, considerable attention has been directed toward the development of sustainable and environmentally friendly analytical methodologies.^14^ In accordance with the principles of green analytical chemistry, extraction techniques involving low solvent consumption, minimal waste generation, and environmentally benign materials have gained increasing importance.^15^ In this context, the UA-DMSPE technique represents a promising alternative for sustainable analytical applications by not only providing high extraction performance but also reducing chemical consumption and environmental impact.^16^ Chelating ligands play a crucial role in the selective binding and retention of metal ions. Donor atoms present in ligand structures enable the selective formation of complexes with metal ions through coordination interactions.^17^ In particular, anthraquinone derivatives exhibit a strong tendency to form complexes with heavy metal ions owing to their carbonyl groups, extended π-electron systems, and suitable functional substituents.^18^ Anthraquinones constitute an important class of naturally occurring and synthetically accessible compounds characterized by their chemically versatile quinone framework and rich coordination potential. In addition to their well-documented biological activities, anthraquinone derivatives have attracted increasing attention in environmental and analytical applications owing to their electron-rich oxygen-containing functional groups and strong interaction capability toward metal ions.^19^ The presence of carbonyl and hydroxyl functionalities within anthraquinone structures enables effective coordination with toxic heavy metals, making these compounds promising candidates for the development of selective sorbent materials.^20,21^ Recent studies have demonstrated that anthraquinone-based materials can exhibit efficient binding behavior toward hazardous metal species, highlighting their potential applicability in environmental remediation and metal preconcentration studies.^22^ Moreover, the electronic distributions and charge transfer characteristics of these compounds directly influence their interaction behavior toward metal ions.^23^ Therefore, the theoretical evaluation of the electronic properties of ligand structures may provide significant advantages in predicting experimental extraction performance. Density functional theory (DFT) calculations constitute powerful theoretical tools for understanding ligand–metal interactions, evaluating HOMO–LUMO energy levels, and determining chemical reactivity parameters.^24^ Although numerous sorbent materials and extraction approaches have been proposed for heavy metal determination, studies integrating DFT-assisted ligand selection, anthraquinone-based functional sorbents, UA-DMSPE methodology, multivariate CCD optimization, and green analytical assessment for the simultaneous determination of Pb­(II) and Cd­(II) in rice and paddy soil samples remain extremely limited.^25^ Therefore, the development of a selective, environmentally friendly, and theoretically supported analytical strategy for such matrices represents a relevant and timely research objective. In the present study, three novel anthraquinone-derived ligands (Derivative 1, Derivative 2, and Derivative 3), which have not previously been reported in the literature, were synthesized and comparatively investigated for their carrier/chelating properties toward Pb­(II) and Cd­(II) ions through both theoretical and experimental approaches. To rationally identify the most suitable ligand candidate for metal coordination, density functional theory (DFT) calculations were performed, and comparative evaluation of HOMO–LUMO energy levels, electron density distributions, and chemical reactivity descriptors revealed that Derivative 2 possessed more favorable electronic characteristics than the other derivatives. In particular, the lower HOMO–LUMO energy gap of Derivative 2 suggested a greater tendency for metal–ligand interactions and facilitated electron transfer processes.^26^ Based on these theoretical findings, Derivative 2 was selected and loaded onto a silica surface to prepare a novel anthraquinone-functionalized sorbent material. The developed sorbent was subsequently applied in an ultrasound-assisted dispersive micro solid-phase extraction (UA-DMSPE) procedure for the preconcentration of Pb­(II) and Cd­(II) ions from both rice and paddy soil samples, while metal determination was carried out using flame atomic absorption spectrometry (FAAS), providing a cost-effective analytical platform for trace metal analysis. To achieve reliable extraction performance, the principal experimental parameters affecting recovery, including ligand concentration, eluent volume, pH, sorbent amount, extraction time, and sample volume, were systematically optimized. Since conventional univariate optimization approaches may be insufficient to explain parameter interactions and generally require a high number of experiments, multivariate optimization was performed using a central composite design (CCD) coupled with response surface methodology (RSM), enabling simultaneous evaluation of variable interactions and determination of optimum conditions with a reduced number of experimental runs.^27^ Accordingly, preliminary working intervals were first established using one-factor-at-a-time optimization, followed by CCD-based multivariate optimization implemented through Stat-Ease software. Validation experiments demonstrated close agreement between predicted and experimental responses, while the adequacy of the quadratic model and the nonsignificant lack-of-fit value confirmed the statistical reliability of the developed method. Finally, the environmental sustainability of the proposed analytical strategy was comprehensively assessed according to the principles of green analytical chemistry using contemporary green metrics, including Analytical Eco-Scale, GAPI, and AGREE, demonstrating the environmentally favorable nature of the developed method in terms of low reagent consumption, minimal waste generation, and reduced environmental impact.

## Experimental Section

### Chemicals and Reagents

All reagents and solvents used throughout the study were of analytical grade and obtained from Merck, Sigma-Aldrich, Fluka or Riedel-de Haen. Silica gel (70–230 mesh) was used as the solid support material for sorbent preparation. Lead­(II) chloride (PbCl_2_) and cadmium­(II) chloride (CdCl_2_), used in analytical studies, were purchased from Merck or Sigma-Aldrich. Stock solutions of Pb­(II) and Cd­(II) (1000 mg L^–1^) were prepared in ultrapure water and stored at +4 °C. Working standard solutions were freshly prepared daily by appropriate dilution of the stock solutions with ultrapure water. Nitric acid (HNO_3_) and hydrochloric acid (HCl), employed for pH adjustment, sample preparation, and elution studies, were of analytical grade. Ultrapure water (resistivity ≥ 18.2 MΩ cm) used throughout all experiments was supplied by a Thermo Scientifi Smart2Pure Pro ultrapure water purification system. Structural elucidation of the synthesized compounds was carried out using a Bruker Avance III HD 600 NMR spectrometer (Bruker BioSpin GmbH, Rheinstetten, Germany) equipped with a 5 mm BBO probe (^1^H/^13^C/^15^N), operating at 600.13 MHz for ^1^H and 150.92 MHz for ^13^C measurements. Infrared spectra were recorded in the range of 4000–650 cm^–1^ using an ATR accessory on a PerkinElmer Spectrum One FT-IR spectrometer. High-resolution mass spectrometry (HR-MS) analyses were performed using a Thermo Scientific Exactive Plus Benchtop Full-Scan Orbitrap LC-HRMS system equipped with a heated electrospray ionization interface. The melting points (m.p.) of the compounds were determined using a Cole-Parmer Stuart MP-200D digital melting point apparatus. The surface morphology and elemental composition of the synthesized sorbent were examined using scanning electron microscopy coupled with energy-dispersive X-ray spectroscopy (SEM–EDX) on a Zeiss EVO HD15 instrument equipped with a tungsten electron source. Elemental compositions (C, H and N) were determined using a LECO CHNS-O-9320 elemental analyzer. Density functional theory (DFT) calculations were performed to evaluate the electronic properties and metal-binding tendencies of the synthesized anthraquinone derivatives. Based on Frontier molecular orbital analysis and calculated chemical reactivity descriptors, Derivative 2 was selected for experimental sorbent preparation and subsequent analytical applications. Ultrasound-assisted dispersive micro solid-phase extraction (UA-DMSPE) experiments were carried out using a Bandelin ultrasonic bath. Quantitative determination of Pb­(II) and Cd­(II) ions was performed using a PerkinElmer AAnalyst 700 flame atomic absorption spectrometer (FAAS) equipped with hollow cathode lamps. The analytical wavelengths employed for metal determination were 217.0 nm for Pb­(II) and 228.8 nm for Cd­(II), selected according to manufacturer recommendations and relevant literature data. Instrumental parameters were individually optimized to ensure maximum sensitivity, precision, and signal stability.

### Real Samples Preparation

Rice samples were collected from paddy cultivation areas and thoroughly washed with ultrapure water to remove adhering surface impurities. The samples were dried at 60–70 °C until constant weight and subsequently ground into fine powder using a laboratory grinder. Approximately 0.50 g of the homogenized rice sample was accurately weighed into a digestion vessel, followed by the addition of 8 mL HNO_3_ (65%) and 2 mL H_2_O_2_ (30%). The mixture was initially left at room temperature for 30 min for predigestion and then heated on a hot plate at 120 °C until a clear solution was obtained. After cooling, the digested solution was filtered through 0.45 μm membrane filter and diluted to 25.0 mL with ultrapure water.^28^ The resulting solution was subjected to the proposed UA-DMSPE procedure prior to FAAS analysis. Paddy soil samples were collected from rice cultivation fields, air-dried at room temperature, homogenized, and sieved through a 100-mesh sieve to remove stones and plant residues. Approximately 0.50 g of dried soil sample was transferred into a digestion vessel, followed by the addition of 9 mL HNO_3_ (65%), 3 mL HCl (37%), and 2 mL H_2_O_2_ (30%). The mixture was gradually heated on a hot plate at 120–140 °C until near dryness. After cooling, the residue was dissolved in 1% HNO_3_, filtered through a 0.45 μm membrane filter, and diluted to 25.0 mL with ultrapure water.^29^ The obtained solution was then subjected to the developed UA-DMSPE method prior to FAAS determination.

### General Synthetic Procedure for Novel Anthraquinone Derivatives 1–3

The mixture of DHA (1,8-dihydroxyanthraquinone, 1,4-dihydroxyanthraquinone, or 1,2-dihydroxyanthraquinone) (2 mmol, 0.48 g), K_2_CO_3_ (2 mmol, 0.28 g), 2-(bromomethyl)­isoindoline-1,3-dione (2 mmol, 0.48 g), and DMF (30 mL) was stirred for 48 h under reflux. After the finished reaction time, the mixture was cooled and poured into cold water (100 mL). The precipitated bright-yellow solid was filtered. The solid was purified via column chromatography with a mixture of dichloromethane: diethyl ether (3:1 mL) (Figure 1).

**Figure 1:**
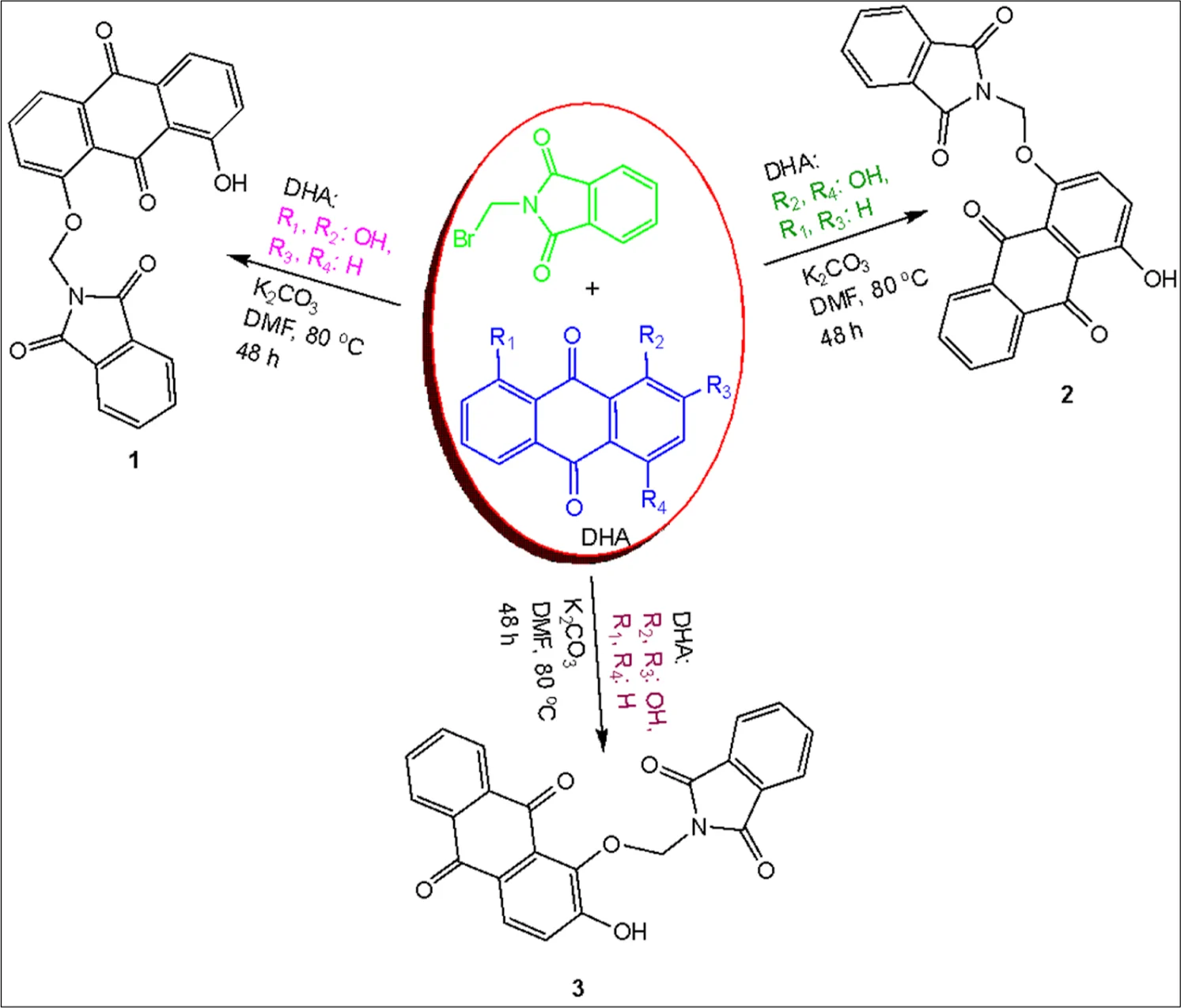
Synthetic pathway for the preparation of novel anthraquinone Derivatives **1**–**3**.

#### Synthesis of Derivative 1:2-(((8-Hydroxy-9,10-dioxo-9,10-dihydroanthracen-1-yl)­oxy)­methyl)­isoindoline-1,3-dione

Color: bright yellow, yield: 85%, m.p.: 246–247 °C. FT-IR: υ­(C=O)­anthraquinone: 1670 cm^–1^, υ­(C=O)­isoindoline: 1782, 1723 cm^–1^, υ­(C–O): 1239 cm^–1^. LC-HRMS (ESI+); found, [M + Na]^+^: 422.0596 *m*/*z*. Calcd: [Na + C_23_H_13_NO_6_]^+^: 422.25 *m*/*z*. ^1^H NMR (CDCl_3_): δ 12.78 (s, OH, 1H), 8.10 (d, Ar–H, 1H, *J* = 12 Hz), 7.94–7.93 (m, Ar–H, 2H), 7.80–7.77 (m, Ar–H, 3H), 7.74 (t, Ar–H, 1H, *J* = 9 Hz), 7.62 (t, Ar–H, 1H, *J* = 9 Hz), 7.58 (d, Ar–H, 1H, *J* = 6 Hz), 7.28 (d, Ar–H, 1H, *J* = 12 Hz), 5.85 (s, CH_2_, 2H) ppm. ^13^C NMR (CDCl_3_): δ 188.3 and 182.4 (C=O)_anthraquinone_, 167.1 (C=O)_isoindoline_, 162.5, 157.7, 136.1, 135.8, 134.7, 132.7, 131.7, 125.4, 124.8, 124.1, 123.5, 123.1, 119.0, 116.9 (Ar–C), 67.6 (CH_2_) ppm.

#### Synthesis of Derivative 2:2-(((4-hydroxy-9,10-dioxo-9,10-dihydroanthracen-1-yl)­oxy)­methyl)­isoindoline-1,3-dione

Color: bright yellow, yield: 80%., m.p.: 271–272 °C. FT-IR: υ­(C=O)­anthraquinone: 1671 cm^–1^, υ­(C=O)­isoindoline: 1772, 1726 cm^–1^, υ­(C–O): 1240 cm^–1^. LC-HRMS (ESI+); found, [M + Na]^+^: 422.0561 *m*/*z*. Calcd: [Na + C_23_H_13_NO_6_]^+^: 422.25 *m*/*z*. ^1^H NMR (CDCl_3_): δ 13.08 (s, OH, 1H), 8.29–8.28 (m, Ar–H, 1H), 8.26–8.24 (m, Ar–H, 1H), 7.92–7.90 (m, Ar–H, 2H), 7.80–7.77 (m, Ar–H, 4H), 7.42 (d, Ar–H, 1H, *J* = 12 Hz), 7.24 (d, Ar–H, 1H, *J* = 12 Hz), 5.77 (s, CH_2_, 2H) ppm. ^13^C NMR (CDCl_3_): δ 188.7 and 181.7 (C=O)_anthraquinone_, 167.3 (C=O)_isoindoline_, 160.0, 150.5, 134.8, 134.6, 134.5, 133.7, 132.8, 132.4, 131.8, 129.4, 127.4, 127.1, 126.5, 126.1, 124.0 (Ar–C), 68.8 (CH_2_) ppm.

#### Synthesis of Derivative 3:2-(((2-hydroxy-9,10-dioxo-9,10-dihydroanthracen-1-yl)­oxy)­methyl)­isoindoline-1,3-dione

Color: yellow, yield: 60%, m.p.: 257–258 °C. FT-IR: υ­(C=O)­anthraquinone: 1666 cm^–1^, υ­(C=O)­isoindoline: 1784, 1724 cm^–1^, υ­(C–O): 1259 cm^–1^. LC-HRMS (ESI+); found, [M + Na]^+^: 422.0597 *m*/*z*. Calcd: [Na + C_23_H_13_NO_6_]^+^: 422.25 *m*/*z*. ^1^H NMR (CDCl_3_): δ 12.97 (s, OH, 1H), 8.32–8.30 (m, Ar–H, 2H), 7.94–7.93 (m, Ar–H, 2H), 7.85–7.79 (m, Ar–H, 5H), 7.48 (d, Ar–H, 1H, *J* = 12 Hz), 5.85 (s, CH_2_, 2H) ppm. ^13^C NMR (DMSO-*d*_6_): δ 189.3 and 181.1 (C=O)_anthraquinone_, 153.2 (C=O)_isoindoline_, 151.2, 135.6, 134.6, 134.0, 133.3, 127.2, 127.0, 124.3, 123.8, 121.6, 121.3, 116.8 (Ar–C), 66.7 (CH_2_) ppm.

### Preparation of Sorbent

Prior to ligand loading, silica gel was activated by washing with 0.5 mol L^–1^ HNO_3_ to remove surface impurities and trace metal contaminants, followed by extensive rinsing with ultrapure water until neutral pH was achieved.^30^ The activated silica was subsequently dried and used as the support material for ligand loading. For sorbent preparation, three different anthraquinone-derived ligands, designated as Derivative 1, Derivative 2, and Derivative 3, were separately loaded onto the silica surface. Briefly, 1 g of pretreated silica was mixed with 10 mL of a 1 × 10^–4^ mol L^–1^ chloroform solution of each synthesized ligand, and the resulting suspensions were stirred at room temperature for 24 h to promote interactions between the ligand molecules and the surface silanol groups of silica.^31^ After the loading process, the solid materials were separated by filtration using a sintered glass funnel, thoroughly washed with chloroform and ultrapure water to remove unbound or weakly retained ligand molecules, and dried at room temperature. The resulting sorbents were designated as Derivative 1–SiO_2_, Derivative 2–SiO_2_, and Derivative 3–SiO_2_. Since no silanization reagent or coupling agent was employed during sorbent preparation, the ligand is considered to be noncovalently immobilized on the silica surface, most likely through hydrogen-bonding interactions between the surface silanol groups and the oxygen-containing functional groups of the ligand, together with weaker van der Waals interactions. Although DFT calculations indicated that Derivative 2 possessed more favorable electronic properties and a stronger interaction tendency toward metal ions, all three sorbents were experimentally evaluated under optimum extraction conditions to comparatively assess their extraction performance toward Pb­(II) and Cd­(II) ions. Based on both theoretical predictions and experimental findings, the Derivative 2-loaded silica sorbent exhibited superior extraction performance and was therefore selected for further optimization and real sample applications. The prepared sorbents were characterized by FT-IR, SEM surface morphology analysis, and elemental analysis to confirm successful ligand loading onto the silica surface prior to their application in UA-DMSPE experiments.

### UA-DMSPE Procedure for Metal Ion Extraction

Initial extraction experiments were conducted under preliminary conditions to establish a basis for subsequent optimization studies. For the preliminary extraction procedure, 10.0 mL of the metal solution was transferred into a polypropylene centrifuge tube, and 40 mg of ligand-functionalized silica sorbent was added. The pH of the medium was adjusted using dilute HNO_3_ and NaOH solutions. The resulting suspension was subjected to ultrasonication in an ultrasonic bath at room temperature to ensure effective dispersion of the sorbent within the solution and rapid adsorption of Pb­(II) and Cd­(II) ions onto the active binding sites of the sorbent. Following the extraction step, phase separation was achieved by centrifugation at 5000 rpm for 10 min, and the supernatant was carefully decanted. For the desorption of the retained metal ions, 1.0 mL of 1.0 mol L^–1^ HNO_3_ solution was added onto the collected sorbent, followed by a second ultrasonication step. The mixture was subsequently centrifuged again under the same conditions (5000 rpm for 10 min), and the obtained eluate was collected for metal determination.^32^ Following optimization studies, all subsequent experiments, calibration studies, and real sample analyses were performed under the optimized conditions unless otherwise stated. The optimum extraction conditions were established as pH 6.0, 30 mg sorbent amount, 20 min extraction time, 10.0 mL sample volume, and elution using 1.0 mL of 0.5 mol L^–1^ HNO_3_. Under these conditions, the determination of Pb­(II) and Cd­(II) ions was performed using flame atomic absorption spectrometry (FAAS). The same optimized procedure was subsequently applied to pretreated rice and paddy soil samples without modification. All experiments were carried out in triplicate together with procedural blanks and spiked samples to evaluate the accuracy of the method. The selectivity of the proposed method was assessed through interference and matrix tolerance studies, whereas the regeneration and reusability of the sorbent were investigated by consecutive adsorption–desorption cycles.

### Density Functional Theory

Density Functional Theory (DFT) calculations were performed to investigate the electronic structure and physicochemical properties of the synthesized Derivative 1, Derivative 2, and Derivative 3 ligands, together with the optimized Derivative 2–Pb­(II) and Derivative 2–Cd­(II) complexes. Molecular structures were initially constructed and preoptimized using ChemDraw and Avogadro, while molecular visualization and orbital analyses were carried out with GaussView 5.0. All quantum-chemical calculations were performed using the Gaussian 09W software package.^33^ Geometry optimizations of the free ligands were carried out using the B3LYP hybrid functional together with the 6–311G basis set for C, H, N, and O atoms, whereas the Pb­(II) and Cd­(II) complexes were optimized at the B3LYP level employing the 6–31G­(d) basis set for the ligand atoms and the LANL2DZ effective core potential for the metal centers.^34^ Vibrational frequency calculations were subsequently performed for all optimized structures to obtain the thermodynamic parameters (*E*_0_, Δ*H*, and Δ*G*) used in the theoretical analysis.

To evaluate charge distribution and identify potential reactive regions, molecular electrostatic potential (MEP) maps were generated. In these maps, regions with positive electrostatic potential are represented in blue, whereas regions exhibiting negative electrostatic potential are shown in red. Areas with intermediate potential values are displayed in green, enabling visualization of weakly polarized regions and probable interaction sites within the molecular framework.^35^ In addition, Frontier molecular orbital (FMO) analysis was conducted to examine the energies and spatial distributions of the highest occupied molecular orbital (HOMO) and lowest unoccupied molecular orbital (LUMO).^36^ These orbitals provide important information regarding electron-donating and electron-accepting tendencies, molecular reactivity, kinetic stability, and ligand–metal interaction characteristics.^37^ The HOMO–LUMO energy gap (Δ*E*) was evaluated as an important descriptor of electronic stability and chemical reactivity. Furthermore, global reactivity parameters, including ionization potential, electron affinity, electronegativity, chemical hardness, softness, chemical potential, and electrophilicity index, together with the thermodynamic parameters (*E*_0_, Δ*H*, and Δ*G*), were calculated using theoretical relationships reported in the literature,^38^ providing a comprehensive quantum-chemical interpretation of both the free ligands and the optimized metal complexes.

1 $${\Delta E}_{gap}=|E_{HOMO}-E_{LUMO}|$$

2 $$I=-E_{HOMO}$$

3 $$EA=-E_{LUMO}$$

4 $$\chi =\frac{I+EA}{2}$$

5 $$\eta =\frac{E_{LUMO}-E_{HOMO}}{2}$$

6 $$\sigma =\frac{1}{2\eta }$$

7 $$\mu =\frac{E_{HOMO}+E_{LUMO}}{2}$$

8 $$\omega =\frac{\mu^{2}}{2\eta }$$

## Results and Discussion

### DFT-Based Selection of the Optimal Anthraquinone Derivative

Prior to sorbent preparation, density functional theory (DFT) calculations were performed to comparatively evaluate the electronic properties, charge distribution characteristics, and metal-binding potential of the newly synthesized anthraquinone derivatives. Since the extraction performance of functional sorbents is strongly influenced by the electronic structure and coordination behavior of the ligand, DFT analysis was employed as a rational approach to identify the most suitable derivative for Pb­(II) and Cd­(II) binding. Particular emphasis was placed on optimized molecular geometries, Frontier molecular orbitals (HOMO–LUMO), molecular electrostatic potential (MEP) distributions, and global reactivity descriptors of the free ligands. Furthermore, to provide direct theoretical evidence for metal coordination and to validate the ligand selection, additional DFT calculations were carried out for the optimized Derivative 2–Pb­(II) and Derivative 2–Cd­(II) complexes, including analyses of their optimized geometries, electronic properties, and thermodynamic stability. The combined results established a comprehensive theoretical basis for selecting the most appropriate ligand for sorbent fabrication.

### Optimized Molecular Geometries

The optimized molecular geometries of Derivative 1, Derivative 2, Derivative 3, Derivative 2–Pb­(II), and Derivative 2–Cd­(II), obtained at the B3LYP level of theory, are presented in Figure 2. The three free ligands possess a common anthraquinone-based molecular framework; however, noticeable differences in molecular conformation and substituent orientation were observed depending on the position of the hydroxyl group. These structural variations influence the spatial arrangement of the donor oxygen atoms and may consequently affect their accessibility for metal coordination. Geometry optimization therefore provides a reliable structural basis for evaluating the relationship between molecular conformation and coordination behavior.^39–42^ Following the identification of Derivative 2 as the most promising free ligand based on its electronic properties, additional geometry optimizations were performed for the Derivative 2–Pb­(II) and Derivative 2–Cd­(II) complexes. The optimized structures confirmed stable O,O-chelated coordination through the deprotonated phenolic oxygen and the adjacent anthraquinone carbonyl oxygen, supporting the structural feasibility of complex formation. These optimized geometries served as the foundation for the subsequent analyses of electronic structure, charge distribution, and thermodynamic stability, which together provide a comprehensive theoretical evaluation of the metal-binding behavior of Derivative 2.^43–45^

**Figure 2:**
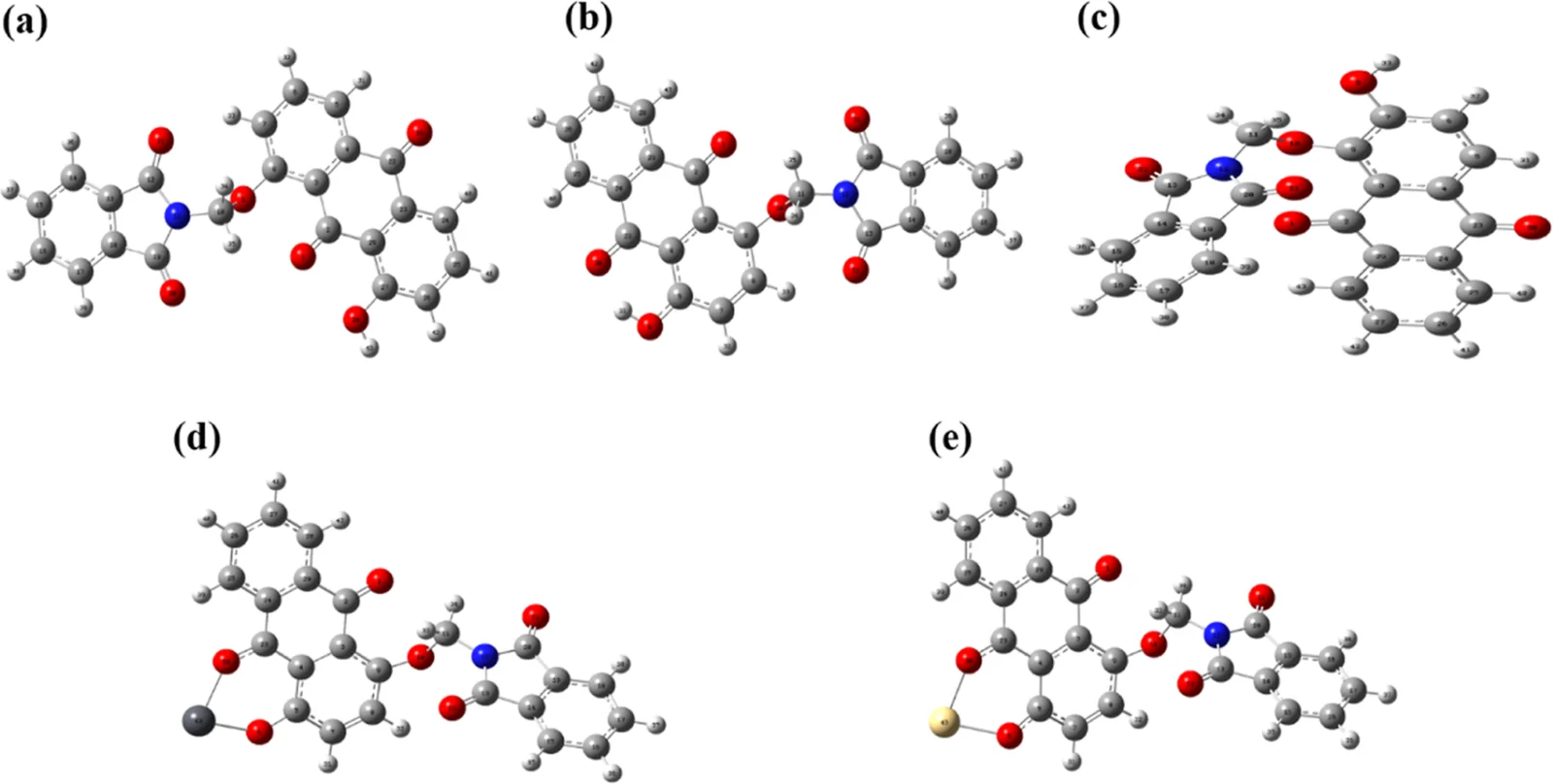
Optimized geometries of (a) Derivative 1 (b) Derivative 2 (c) Derivative 3, (d) Derivative 2 Pb and (e) Derivative 2-Cd illustrating the electronic charge distribution and potential coordination regions.

### Molecular Electrostatic Potential Analysis

The molecular electrostatic potential (MEP) maps of Derivative 1, Derivative 2, Derivative 3, Derivative 2–Pb­(II), and Derivative 2–Cd­(II) are presented in Figure 3, providing valuable insight into the electronic charge distribution and the potential coordination sites involved in metal binding. For the three free ligands, regions of negative electrostatic potential were predominantly localized around the oxygen-containing functional groups, particularly the carbonyl and hydroxyl oxygen atoms, identifying these sites as the most probable coordination centers for Pb­(II) and Cd­(II) ions.^46^ In contrast, regions of positive electrostatic potential were mainly distributed over hydrogen-bearing aromatic domains, corresponding to electron-deficient regions of the molecular framework. Among the free ligands, Derivative 2 exhibited a more continuous distribution of negative electrostatic potential over the oxygen donor region, indicating a more favorable electronic environment for metal coordination than Derivative 1 and Derivative 3. This observation is consistent with the Frontier molecular orbital and global reactivity analyses, which demonstrated enhanced electronic flexibility and charge redistribution for Derivative 2.^46^ Following complex formation, the MEP distributions of the Derivative 2–Pb­(II) and Derivative 2–Cd­(II) complexes revealed a noticeable redistribution of electrostatic potential around the coordination sphere, reflecting ligand-to-metal electron donation and confirming the participation of the deprotonated phenolic oxygen and the adjacent anthraquinone carbonyl oxygen in metal coordination. These results, together with the optimized geometries and global reactivity descriptors, provide complementary theoretical evidence supporting the superior coordination behavior of Derivative 2 toward Pb­(II) and Cd­(II) ions.

**Figure 3:**
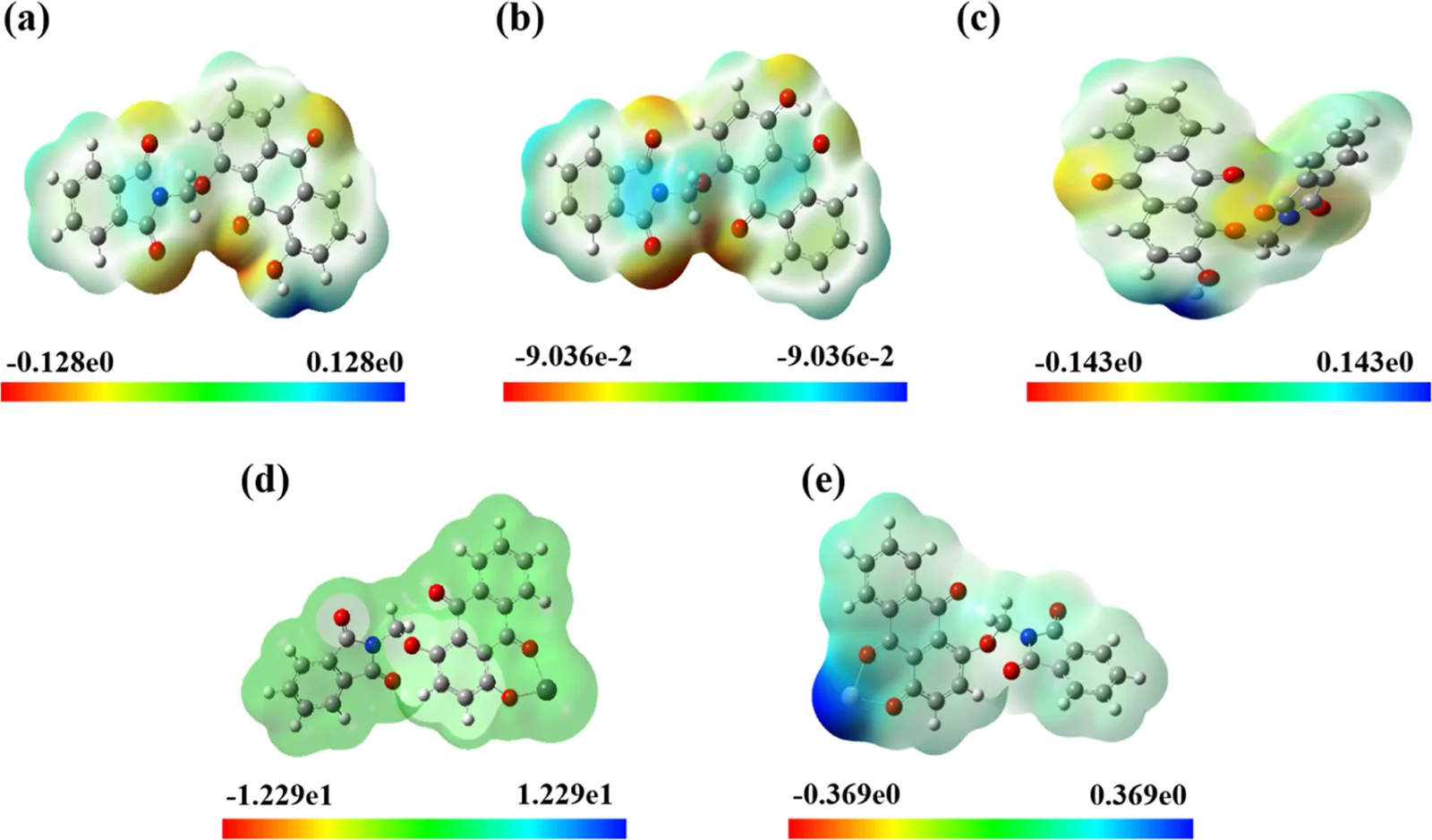
Molecular electrostatic potential (MEP) maps of (a) Derivative 1, (b) Derivative 2, (c) Derivative 3, (d) Derivative 2 Pb and (e) Derivative 2-Cd illustrating the electronic charge distribution and potential coordination regions.

### Frontier Molecular Orbital Analysis

Frontier molecular orbital (FMO) analysis was performed to obtain further insight into the electronic properties and reactivity of the investigated anthraquinone derivatives. The HOMO and LUMO distributions together with the corresponding energy gaps (Δ*E*) are presented in Figure 4. For the three free ligands, the HOMO was predominantly distributed over the π-conjugated aromatic framework and oxygen-containing donor groups, indicating the regions with the greatest electron-donating capability. In contrast, the LUMO was mainly localized around the carbonyl-containing moieties and adjacent aromatic carbons, representing the most favorable electron-accepting regions during intermolecular interactions.^47–49^ Notable differences in Frontier orbital distributions were observed among the investigated derivatives. Derivative 2 exhibited a more extensive HOMO and LUMO delocalization than Derivative 1 and Derivative 3, suggesting more efficient electronic communication throughout the molecular framework. Such orbital delocalization is commonly associated with enhanced charge redistribution and improved chemical reactivity.^50,51^ Consistent with these observations, Derivative 2 exhibited the lowest HOMO–LUMO energy gap (Δ*E* = 2.1284 eV), compared with Derivative 3 (2.2296 eV) and Derivative 1 (2.5662 eV), indicating greater electronic flexibility among the free ligands. Additional FMO calculations performed for the optimized Derivative 2–Pb­(II) and Derivative 2–Cd­(II) complexes revealed a further decrease in the HOMO–LUMO energy gaps to 1.5415 and 1.6511 eV, respectively, accompanied by increased orbital delocalization over the coordination environment. These results indicate that complex formation enhances electronic communication between the ligand and the coordinated metal center and are fully consistent with the global reactivity descriptors and optimized geometries, providing additional theoretical support for the superior coordination behavior of Derivative 2 toward Pb­(II) and Cd­(II) ions (Figure 4).

**Figure 4:**
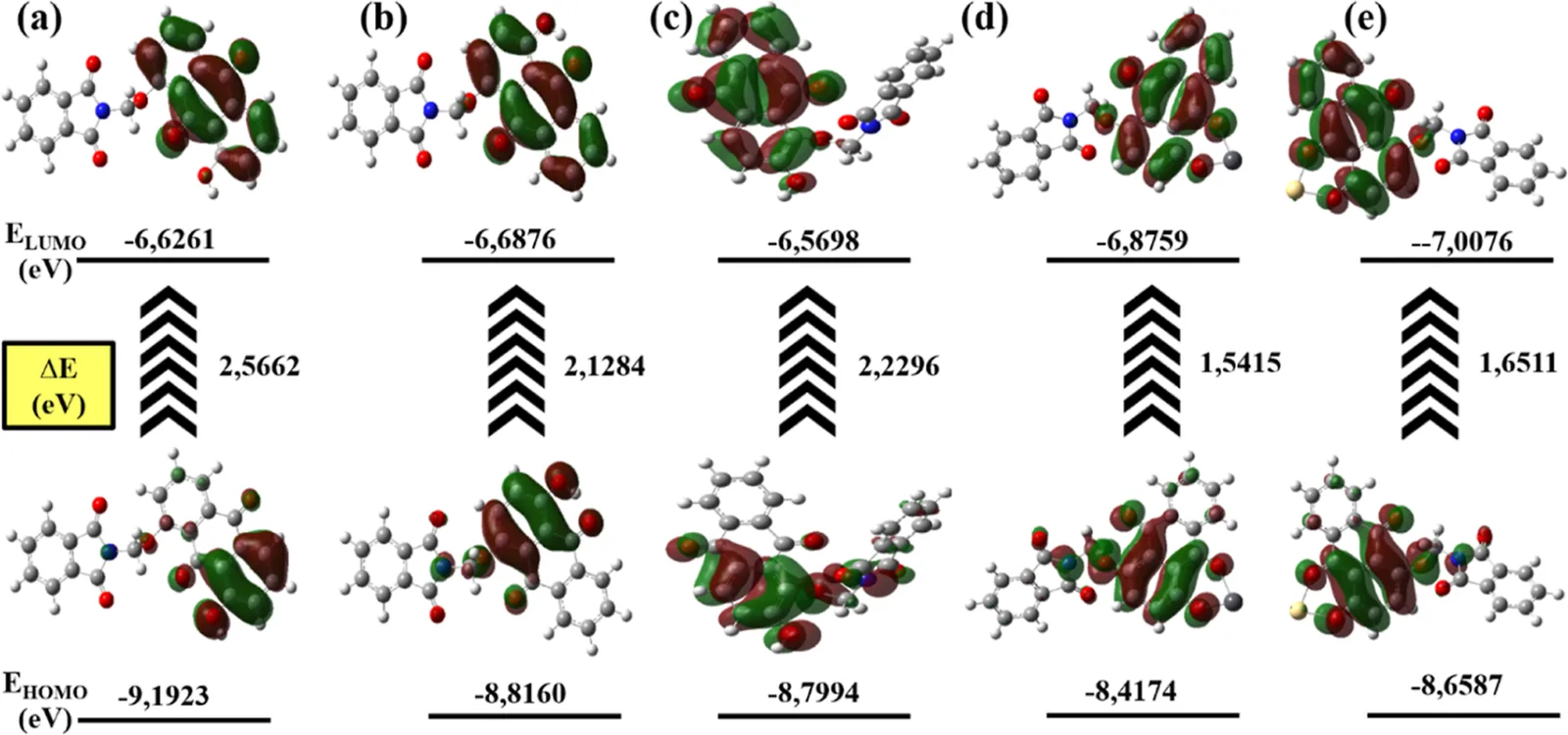
Frontier molecular orbital (HOMO–LUMO) distributions and corresponding energy gaps (Δ*E*) of (a) Derivative 1, (b) Derivative 2, (c) Derivative 3, (d) Derivative 2 Pb and (e) Derivative 2-Cd.

The different coordination behavior of the investigated derivatives can be attributed to the positional effect of the hydroxyl substituent on the anthraquinone framework. In Derivative 2, the hydroxyl group occupies a position that promotes more effective electronic communication with the neighboring carbonyl functionality through the conjugated π-system, resulting in greater electron delocalization over the O,O donor region. Consequently, Derivative 2 exhibits a lower HOMO–LUMO energy gap, higher softness, enhanced electrophilicity, and a more favorable electrostatic environment for metal coordination than Derivative 1 and Derivative 3. These findings provide a structural and electronic rationale for the superior Pb­(II) and Cd­(II) coordination behavior observed for Derivative 2.

### Global Reactivity Descriptors

Among the investigated free ligands, Derivative 2 exhibited the lowest chemical hardness (η = 1.0642) and the highest softness (σ = 0.4700), indicating enhanced electronic flexibility and a greater tendency for charge redistribution. Lower hardness values are generally associated with increased chemical reactivity and stronger interaction capability toward metal ions, whereas higher softness reflects improved adaptability during coordination processes.^52^ Furthermore, Derivative 2 displayed the highest electrophilicity index (ω = 28.2323) among the free ligands, suggesting superior electron-accepting ability and a stronger tendency to participate in charge-transfer interactions.^53^ In contrast, Derivative 1 exhibited the highest hardness and the lowest softness, indicating a relatively more stable but less reactive electronic structure, whereas Derivative 3 showed intermediate behavior. These global reactivity descriptors were consistent with the MEP and Frontier molecular orbital analyses, supporting the prediction that Derivative 2 possesses the most favorable electronic characteristics for Pb­(II) and Cd­(II) coordination. To further validate this theoretical prediction, geometry optimizations of the Derivative 2–Pb­(II) and Derivative 2–Cd­(II) complexes were subsequently performed. Upon complex formation, both complexes exhibited a further reduction in the HOMO–LUMO energy gap (Δ*E* = 1.5415 eV for Derivative 2–Pb and 1.6511 eV for Derivative 2–Cd), accompanied by lower hardness (η = 0.7707 and 0.8256, respectively), higher softness (σ = 0.6487 and 0.6056, respectively), and markedly increased electrophilicity indices (ω = 37.9314 and 37.1610, respectively) compared with the free ligand. These findings demonstrate that metal coordination enhances electron delocalization and charge-transfer capability, providing direct theoretical evidence that corroborates the superior coordination behavior of Derivative 2 toward Pb­(II) and Cd­(II) ions (Table 1).

**Table 1:** Calculated Global Reactivity Descriptors of Derivative 1, Derivative 2, Derivative 3, Derivative 2 Pb and Derivative 2-Cd Obtained From DFT Calculations

chemical parameters	derivative 1	derivative 2	derivative 3	derivative 2 Pb	derivative 2-Cd
EHOMO	–9.1923	–8.8160	–8.7994	–8,4174	–8,6587
ELUMO	–6.6261	–6.6876	–6.5698	–6,8759	–7,0076
Δ*E* energy gap	2.5662	2.1284	2.2296	1,5415	1,6511
ionization potential (IP)	9.1923	8.8160	8.7994	8,4174	8,6587
electron affinity (EA)	6.6261	6.6876	6.5698	6,8759	7,0076
electronegativity (χ)	7.9092	7.7518	7.6846	7,6466	7,8331
hardness (η)	1.2831	1.0642	1.1148	0,7707	0,8256
softness (σ)	0.3897	0.4700	0.4482	0,6487	0,6056
chemical potential (μ)	–7.9092	–7.7518	–7.6846	–7,6466	–7,8331
global electrophilicity (ω)	24.3763	28.2323	26.4852	37,9314	37,1610
*E*_0_ (kcal/mol)	196.9205	196.8767	197.0748	189,8278	190,3323
Δ*H* (298 K)(kcal/mol)	211.9762	211.1394	212.0803	205,7036	206,0908
Δ*G* (298 K)(kcal/mol)	162.5659	162.0671	162.9864	153,3812	153,5751

Based on the combined interpretation of optimized molecular geometries, molecular electrostatic potential (MEP) distributions, Frontier molecular orbital characteristics, global reactivity descriptors, and the DFT investigation of the Derivative 2–Pb­(II) and Derivative 2–Cd­(II) complexes, Derivative 2 was identified as the most promising ligand candidate for Pb­(II) and Cd­(II) coordination. The optimized complexes exhibited thermodynamically favorable coordination together with enhanced electron delocalization, reduced chemical hardness, increased softness, and higher electrophilicity compared with the free ligand, providing direct theoretical evidence for its superior metal-binding capability. Accordingly, Derivative 2 was selected for immobilization onto the silica surface and employed in all subsequent experimental extraction studies. The consistency between the computational predictions and the experimental recovery results further confirmed the suitability of Derivative 2 as the functional ligand for the selective extraction of Pb­(II) and Cd­(II) ions.

### FT-IR Spectroscopic Characterization of the Ligand-Functionalized Silica Sorbents

The successful loading of Derivative 2 onto the silica surface was evaluated by FT-IR spectroscopy (Figure 5). The FT-IR spectrum of bare silica (a) exhibited a broad absorption band centered at approximately 3448 cm^–1^, attributed to the stretching vibrations of surface silanol (O–H) groups. Characteristic silica framework vibrations were also observed, including the strong Si–O–Si asymmetric stretching band in the 1000–1100 cm^–1^ region, together with bands at approximately 808 cm^–1^ and 514 cm^–1^, corresponding to symmetric Si–O–Si stretching and Si–O bending vibrations, respectively, confirming the structural integrity of the silica matrix. The FT-IR spectrum of Derivative 2 (b) displayed characteristic absorption bands associated with its molecular structure. The broad band around 3448 cm^–1^ was attributed to phenolic O–H stretching vibrations, while the distinct absorption band at approximately 1727 cm^–1^ was assigned to the carbonyl (C=O) stretching vibration characteristic of the anthraquinone/phthalimide moiety. Additional absorption bands in the 1630–1350 cm^–1^ region were attributed to aromatic skeletal vibrations and conjugated structural features, whereas the band near 1222 cm^–1^ corresponded to C–O–C stretching vibrations. In the FT-IR spectrum of the Derivative 2-loaded silica sorbent (c), characteristic absorption bands originating from both silica and the ligand were simultaneously observed, confirming the successful loading of Derivative 2 onto the silica surface. In particular, the persistence of the ligand-related C=O stretching vibration (∼1720 cm^–1^), together with the preserved Si–O–Si framework bands, indicates that the molecular structure of Derivative 2 remained intact after the loading process and that the silica framework was not chemically altered. Furthermore, the broad absorption band around 3404 cm^–1^, attributed to overlapping hydroxyl vibrations of the surface silanol groups and the phenolic hydroxyl group of the ligand, suggests the presence of hydrogen-bonding interactions between the ligand and the silica surface. Since no new absorption bands attributable to the formation of covalent Si–O–C or Si–C bonds were observed, the FT-IR results do not provide evidence for chemical grafting. Instead, the ligand is considered to be noncovalently immobilized on the silica surface, most likely through hydrogen-bonding interactions involving surface silanol groups and the oxygen-containing functional groups of Derivative 2, together with weaker van der Waals interactions. Overall, the coexistence of silica-specific and ligand-derived absorption bands provides supportive evidence for the successful loading of Derivative 2 onto the silica surface.

**Figure 5:**
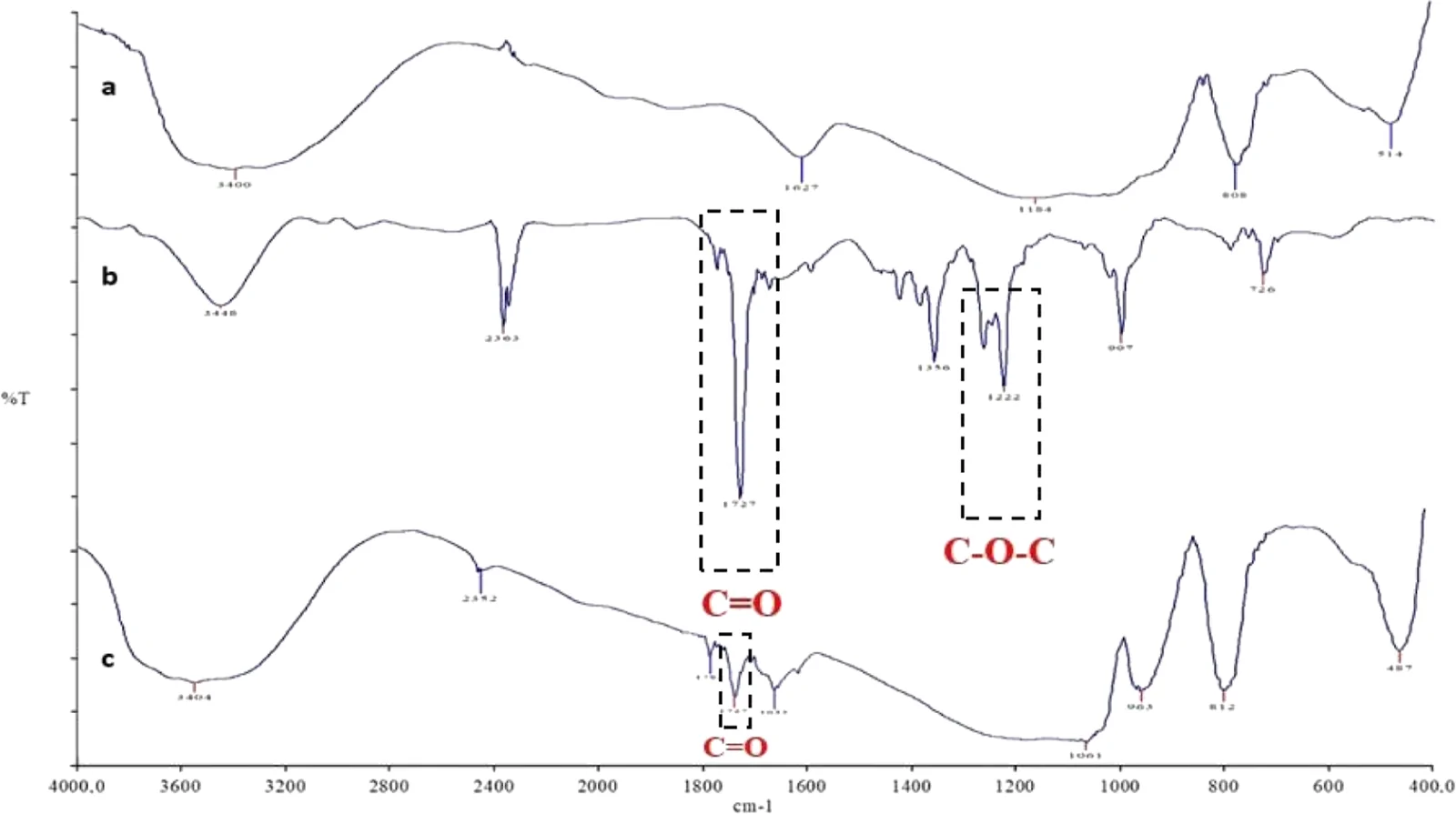
FT-IR spectra of bare silica (a), Derivative 2 (b), and Derivative 2-functionalized silica sorbent (c).

### Elemental Analysis of the Ligand-Functionalized Silica

Elemental CHN analysis was performed to verify the successful immobilization of Derivative 2 onto the silica surface and to estimate the ligand loading. As expected, bare silica exhibited negligible carbon and nitrogen contents (*C* = 0.079%, *H* = 0.842%, *N* = 0.000%), whereas the Derivative 2-loaded silica showed a pronounced increase in the carbon and nitrogen percentages (*C* = 21.218%, *H* = 1.013%, *N* = 1.083%), confirming the presence of the organic ligand on the silica support. The ligand loading, calculated independently from both the carbon and nitrogen contents, was found to be 0.765 and 0.773 mmol g^–1^, respectively. The excellent agreement between these independently calculated values confirms a ligand loading of approximately 0.77 mmol g^–1^ of sorbent. These results demonstrate the successful physical immobilization of Derivative 2 on the silica surface and indicate a high ligand incorporation efficiency (Table 2).

**Table 2:** Elemental CHN Analysis of Bare Silica and Derivative 2-Loaded SiO_2_

sample	C (%)	H (%)	N (%)
SiO_2_	0.079	0.842	0.000
derivative 2-loaded SiO_2_	21.218	1.013	1.083

### Spectroscopic Characterization of Anthraquinone Derivatives

The structure of Derivative 1 was confirmed by FT-IR, ^1^H NMR, ^13^C NMR, and HR-MS analyses. In the FT-IR spectrum, the absorption band at 1670 cm^–1^ was attributed to the anthraquinone carbonyl groups, while the characteristic bands at 1782 and 1723 cm^–1^ corresponded to the carbonyl vibrations of the isoindoline-1,3-dione moiety. In the ^1^H NMR spectrum, the singlet signal observed at δ 12.78 ppm indicated the presence of a phenolic OH proton involved in intramolecular hydrogen bonding. The aromatic proton resonances appeared within the expected region, whereas the singlet signal at δ 5.85 ppm confirmed the presence of the O–CH_2_ group. In the ^13^C NMR spectrum, the anthraquinone carbonyl carbons resonated at δ 188.3 and 182.4 ppm, while the imide carbonyl carbon appeared at δ 167.1 ppm. Furthermore, the HR-MS spectrum exhibited a [M + Na]^+^ ion peak in good agreement with the calculated mass value, supporting the proposed molecular structure. The spectroscopic data of Derivative 2 were likewise consistent with the proposed structure. In the FT-IR spectrum, the anthraquinone carbonyl stretching vibration was observed at 1671 cm^–1^, whereas the characteristic carbonyl absorptions of the isoindoline-1,3-dione unit appeared at 1772 and 1726 cm^–1^. The ^1^H NMR spectrum displayed a phenolic OH singlet at δ 13.08 ppm, indicating strong intramolecular hydrogen bonding. The aromatic proton resonances were distributed in accordance with the anthraquinone–phthalimide framework, while the singlet signal at δ 5.77 ppm confirmed the methylene bridge (O–CH_2_). In the ^13^C NMR spectrum, the anthraquinone carbonyl carbons resonated at δ 188.7 and 181.7 ppm, and the isoindoline carbonyl carbon appeared at δ 167.3 ppm. In addition, the sodium adduct molecular ion peak ([M + Na]^+^) observed in the HR-MS analysis showed excellent agreement with the theoretical mass value, further supporting the assigned structure. Finally, the structure of Derivative 3 was similarly verified using multiple spectroscopic techniques. The FT-IR spectrum exhibited anthraquinone carbonyl absorption at 1666 cm^–1^ together with characteristic isoindoline-1,3-dione carbonyl bands at 1784 and 1724 cm^–1^. In the ^1^H NMR spectrum, the singlet signal observed at δ 12.97 ppm confirmed the presence of the phenolic OH proton, while the aromatic multiplet signals were fully consistent with the proposed anthraquinone derivative. The singlet resonance at δ 5.85 ppm supported the existence of the O–CH_2_ group. In the ^13^C NMR spectrum, the anthraquinone carbonyl carbons were identified at δ 189.3 and 181.1 ppm, whereas the aliphatic methylene carbon resonated at δ 66.7 ppm. Moreover, the [M + Na]^+^ ion peak observed in the HR-MS spectrum was in close agreement with the calculated molecular mass, confirming the successful formation of the target derivative. Detailed FT-IR, ^1^H NMR, ^13^C NMR, and HR-MS spectra of Derivatives 1–3 are provided in the Supporting Information.

### Surface Morphology and Elemental Composition of the Sorbent

The surface morphology of the synthesized sorbent before and after the extraction process was investigated by scanning electron microscopy (SEM), while elemental composition changes were evaluated using SEM–EDX analysis. As shown in Figure 6, the unloaded sorbent exhibited an irregular morphology consisting of fractured and angular silica particles with relatively rough and heterogeneous surfaces, which may favor adsorption by providing accessible interaction sites and increased surface area.^54^ The corresponding EDX spectrum confirmed the presence of the characteristic framework elements (Si and O) together with minor carbon signals associated with the ligand structure, while no detectable signals corresponding to Pb or Cd were observed. Following the extraction process, the general morphology of the sorbent remained largely preserved, although minor localized surface deposits and slight textural changes became noticeable. Since SEM-based morphological differences were relatively limited, the principal evidence for metal retention was obtained from the SEM–EDX analysis. In particular, the appearance of distinct Pb­(II) and Cd­(II) signals in the postextraction EDX spectrum, absent in the unloaded sorbent, clearly confirms the successful adsorption of the target metal ions onto the sorbent surface. Furthermore, the preservation of the overall silica morphology after extraction suggests that the sorbent maintained its structural integrity during the adsorption process, supporting its suitability for repeated analytical use.

**Figure 6:**
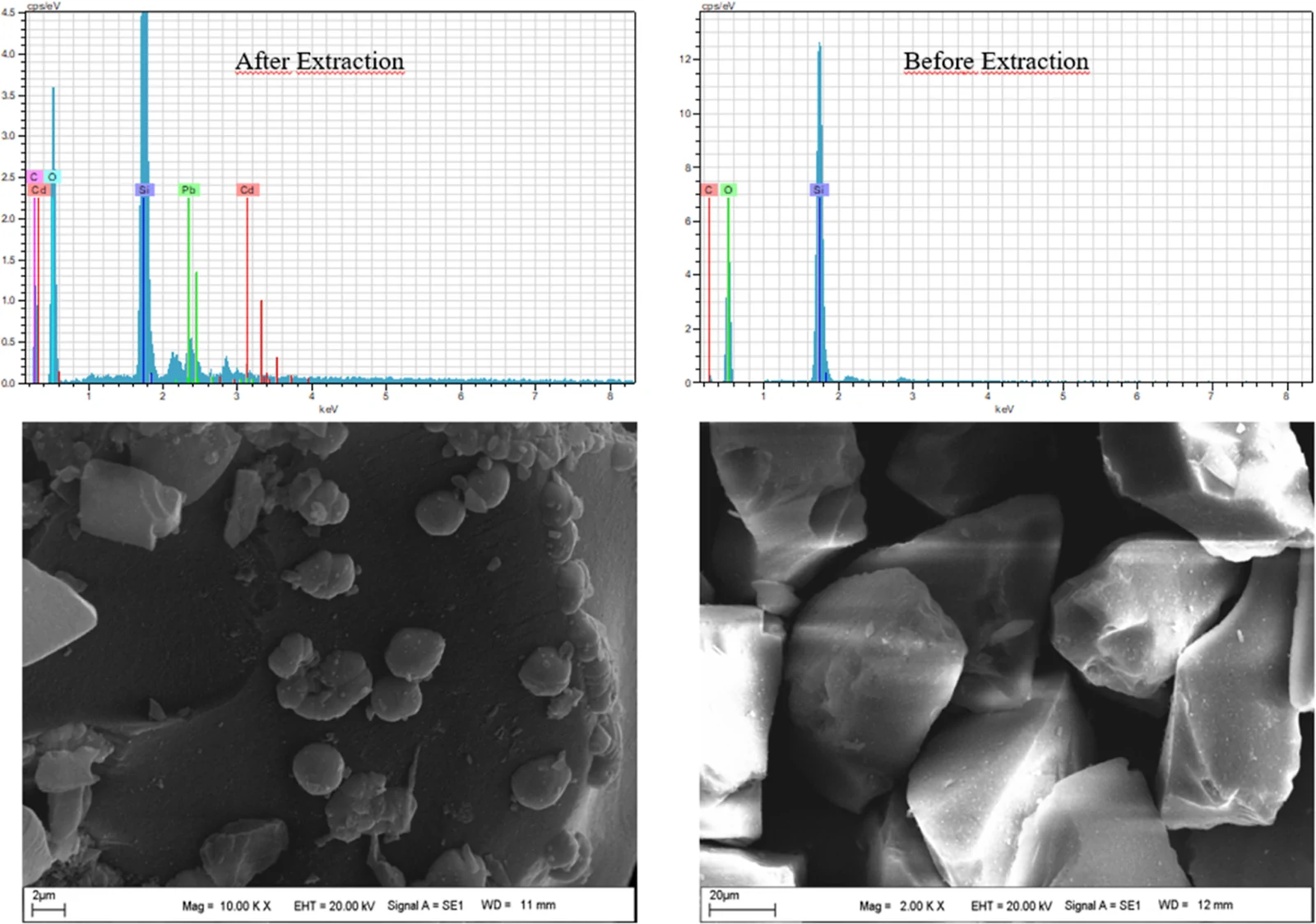
SEM–EDX characterization of the sorbent before and after Pb­(II) and Cd­(II) adsorption.

### DFT-Assisted Interpretation of Pb­(II) and Cd­(II) Adsorption Mechanism

The adsorption of Pb­(II) and Cd­(II) ions onto the Derivative 2-functionalized silica sorbent is mainly governed by coordination interactions occurring between the target metal ions and the oxygen-containing donor groups of the surface-loaded anthraquinone derivative. Based on the molecular structure of Derivative 2, the most probable coordination centers are the carbonyl oxygen atoms of the anthraquinone moiety, the carbonyl groups associated with the phthalimide unit, and the phenolic hydroxyl oxygen atom, which collectively provide electron-rich binding sites for metal ion complexation. The coexistence of multiple oxygen donor atoms within the ligand framework may facilitate stable coordination and enhance adsorption efficiency toward Pb­(II) and Cd­(II) ions. At the optimum pH value (pH 6.0), the metal ions remain predominantly in their soluble ionic forms, while partial deprotonation of the phenolic hydroxyl group may increase electron density around the coordinating oxygen atoms, thereby facilitating metal–ligand interactions. In addition, ultrasonic irradiation improves sorbent dispersion throughout the solution and accelerates mass transfer, allowing rapid contact between the active adsorption sites and target metal ions. The adsorption behavior is further supported by DFT calculations, which demonstrated that Derivative 2 possesses a more favorable electronic distribution and interaction tendency compared with the other synthesized derivatives. In particular, molecular electrostatic potential (MEP) and Frontier molecular orbital analyses suggested that electron-rich oxygen-containing regions represent the most likely interaction sites for metal coordination. During the elution step, nitric acid protonates the donor oxygen atoms and weakens metal–ligand coordination, enabling efficient desorption of retained Pb­(II) and Cd­(II) ions and regeneration of the sorbent.

### Experimental and Statistical Optimization of the UA-DMSPE Procedure

To achieve high extraction efficiency and reliable analytical performance, the principal experimental variables affecting the ultrasound-assisted dispersive micro solid-phase extraction (UA-DMSPE) procedure were systematically optimized. The investigated parameters included ligand concentration, eluent volume, sample pH, sorbent amount, extraction time, and sample volume, all of which may significantly influence the retention efficiency of Pb­(II) and Cd­(II) ions. Initially, preliminary one-factor-at-a-time (OFAT) experiments were performed to establish suitable working ranges for each parameter prior to multivariate optimization. Subsequently, a central composite design (CCD) coupled with response surface methodology (RSM) was employed to simultaneously evaluate the effects of multiple experimental variables, investigate possible interaction effects, and determine the optimum extraction conditions with a reduced number of experimental runs. A six-factor CCD model consisting of ligand concentration (A), eluent volume (B), pH (C), sorbent amount (D), extraction time (E), and sample volume (F) was generated using Stat-Ease software, while extraction recovery (%) was selected as the response variable. The experimental design matrix together with the corresponding recovery values is presented in Table S1. The suitability of the generated mathematical model was evaluated through sequential model sum of squares analysis, model fitting statistics, and analysis of variance (ANOVA). Among the tested polynomial models, the quadratic model was selected as the most appropriate due to its statistically significant contribution (*p* < 0.0001), high predictive capability, and nonaliased structure. Furthermore, the fit statistics demonstrated strong agreement between the adjusted and predicted determination coefficients, while the nonsignificant lack-of-fit value (*p* = 0.2669) confirmed the adequacy of the model for describing the experimental response. The effects and interactions of the selected variables on extraction recovery were further interpreted using contour and three-dimensional response surface plots. Finally, the optimum extraction conditions were predicted through the desirability function approach, and validation experiments conducted under the predicted optimum conditions demonstrated close agreement between experimental and predicted recovery values, confirming the robustness and reliability of the proposed UA-DMSPE procedure.

### Preliminary Univariate (OFAT) Optimization Studies

Prior to multivariate optimization using CCD–RSM, preliminary one-factor-at-a-time (OFAT) experiments were conducted to establish suitable working intervals for the principal experimental variables affecting the UA-DMSPE procedure. In this approach, a single parameter was varied while all remaining variables were kept constant to evaluate its individual influence on extraction recovery. The investigated parameters included ligand concentration, eluent volume, pH, sorbent amount, extraction time, and sample volume. The experimental ranges selected from these preliminary studies were subsequently used as input levels for the CCD model.

### Role of Ligand Loading in Pb­(II) and Cd­(II) Retention

The extraction performance of the functionalized sorbent is strongly influenced by the amount of ligand loaded onto the silica surface, since the density of accessible coordination sites directly governs metal–sorbent interactions. Insufficient ligand loading may result in a limited number of available binding sites, thereby restricting metal ion retention. Conversely, excessive ligand loading may lead to partial surface crowding and reduced accessibility of active coordination centers, thereby negatively affecting extraction efficiency due to steric hindrance and restricted diffusion of metal ions toward the sorbent surface.^55^ To investigate the influence of ligand loading on extraction performance, a series of sorbents was prepared by loading 10 mL of Derivative 2 solutions at concentrations of 1 × 10^–5^, 3 × 10^–5^, 1 × 10^–4^, 3 × 10^–4^, and 5 × 10^–4^ mol L^–1^ onto 1 g of silica, while maintaining all other preparation conditions constant. The extraction recoveries of Pb­(II) and Cd­(II) ions obtained using the prepared sorbents are shown in Figure 7. As the ligand concentration increased from 1 × 10^–5^ to 3 × 10^–4^ mol L^–1^, the recovery values of both Pb­(II) and Cd­(II) ions gradually increased, indicating enhanced metal uptake due to the increased availability of coordination sites on the sorbent surface. Under these conditions, the maximum recoveries of approximately 92% for Pb­(II) and 89% for Cd­(II) were obtained at a ligand concentration of 3 × 10^–4^ mol L^–1^. However, a slight decrease in extraction efficiency was observed at 5 × 10^–4^ mol L^–1^, suggesting that excessive ligand loading may partially reduce surface accessibility and limit effective interaction between metal ions and active binding sites.^56^ Furthermore, the comparable recovery behavior of Pb­(II) and Cd­(II) demonstrates that the developed sorbent possesses favorable affinity toward both target ions and enables their simultaneous preconcentration under similar experimental conditions. Therefore, 3 × 10^–4^ mol L^–1^ was selected as the optimum ligand concentration for subsequent experiments.

**Figure 7:**
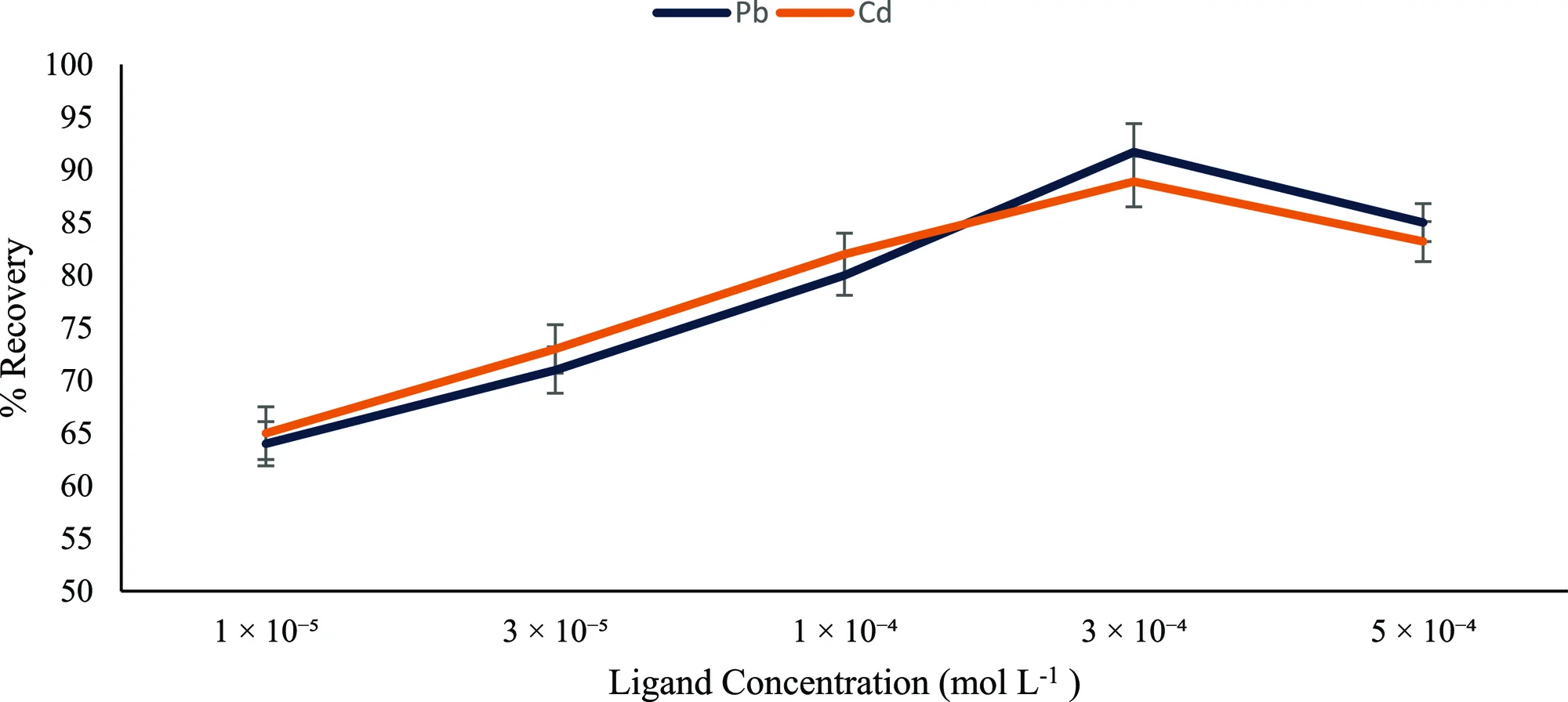
Influence of ligand loading on the extraction performance of Pb­(II) and Cd­(II) (*n* = 3).

### Optimization of Eluent Type and Volume

In UA-DMSPE-based analytical procedures, the effective desorption of retained metal ions from the sorbent phase represents a fundamental step directly influencing extraction efficiency and analytical performance. The selection of an appropriate elution medium is particularly important, as strong interactions established between metal ions and active coordination sites on the sorbent surface must be sufficiently disrupted to enable quantitative recovery. In this context, acidic eluents are widely employed in heavy metal extraction studies due to their ability to protonate donor atoms at the sorbent interface, thereby weakening metal–ligand coordination and promoting efficient release of adsorbed analytes. In the present study, nitric acid (HNO_3_), hydrochloric acid (HCl), and acetic acid (CH_3_COOH) were investigated as potential elution solvents due to their frequent use in heavy metal extraction studies and their compatibility with flame atomic absorption spectrometry (FAAS). Elution experiments were initially carried out using identical volumes of each acid under the same experimental conditions. HNO_3_ exhibited superior desorption efficiency for both Pb­(II) and Cd­(II) compared with HCl and CH_3_COOH, yielding the highest recovery values. This enhanced performance may be attributed to the stronger protonation capability of nitric acid, which more effectively disrupts coordination interactions between metal ions and the functional groups located on the sorbent surface.^57^ Following the selection of nitric acid as the most effective eluent, the influence of acid concentration was investigated using 0.1, 0.25, 0.5, 1.0, and 2.0 mol L^–1^ HNO_3_ solutions in order to establish the optimum desorption conditions. As shown in Figure 8, the recovery values of both Pb­(II) and Cd­(II) gradually increased with increasing acid concentration up to 0.5 mol L^–1^, at which the highest extraction efficiencies were achieved. However, a slight decrease in recovery was observed at higher acid concentrations (1.0–2.0 mol L^–1^), which may be attributed to excessive proton competition and partial destabilization of desorbed metal species in the eluent phase.^58^ Therefore, 0.5 mol L^–1^ HNO_3_ was selected as the optimum elution solvent concentration for subsequent studies. The superior performance of nitric acid observed in the present study is also consistent with previous reports, in which HNO_3_ has frequently been identified as one of the most effective eluents for heavy metal desorption in silica-based extraction systems due to its high elution efficiency and excellent compatibility with FAAS and ICP-based analytical techniques.

**Figure 8:**
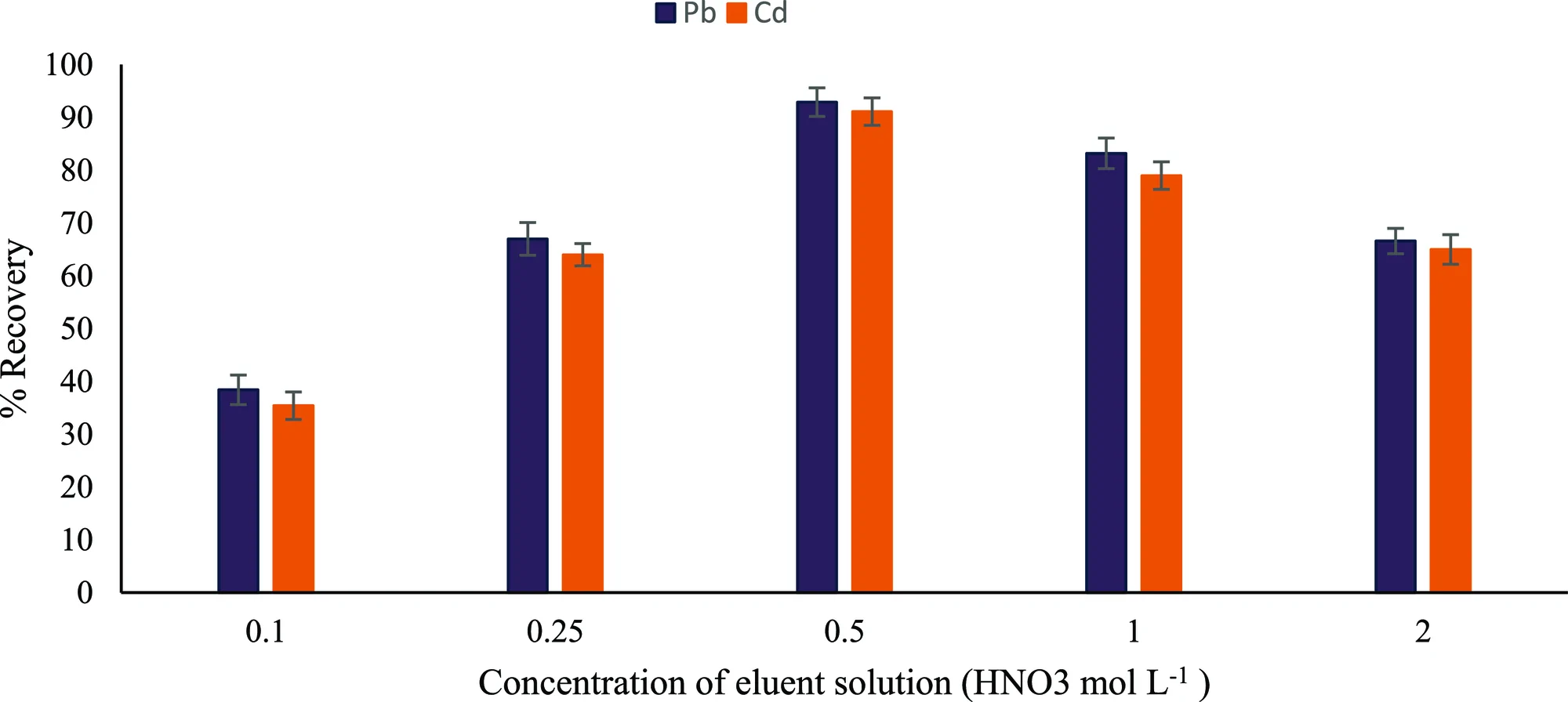
Effect of eluent concentration (HNO_3_, mol L^–1^) on the recovery of Pb­(II) and Cd­(II) ions (*n* = 3).

Optimization of eluent volume is another important parameter in microextraction procedures, as lower eluent volumes may provide higher enrichment factors and improved analytical sensitivity while still ensuring quantitative desorption of analytes. In the present study, elution experiments were performed using different volumes (0.25, 0.50, 1.00, 1.25, and 1.50 mL) of HNO_3_ under identical experimental conditions. As shown in Figure 9, the recovery values of both Pb­(II) and Cd­(II) gradually increased with increasing eluent volume up to 1.00 mL, indicating more effective desorption of retained metal ions from the sorbent surface. The highest recoveries were obtained at 1.00 mL, with approximately 93% for Pb­(II) and 91% for Cd­(II). However, further increases in eluent volume resulted in a slight decrease at 1.25 mL and a more pronounced decline at 1.50 mL, which may mainly be attributed to dilution effects leading to reduced analyte concentration in the final solution rather than incomplete desorption.^59^ Therefore, 1.00 mL HNO_3_ was selected as the optimum eluent volume for all subsequent experiments.

**Figure 9:**
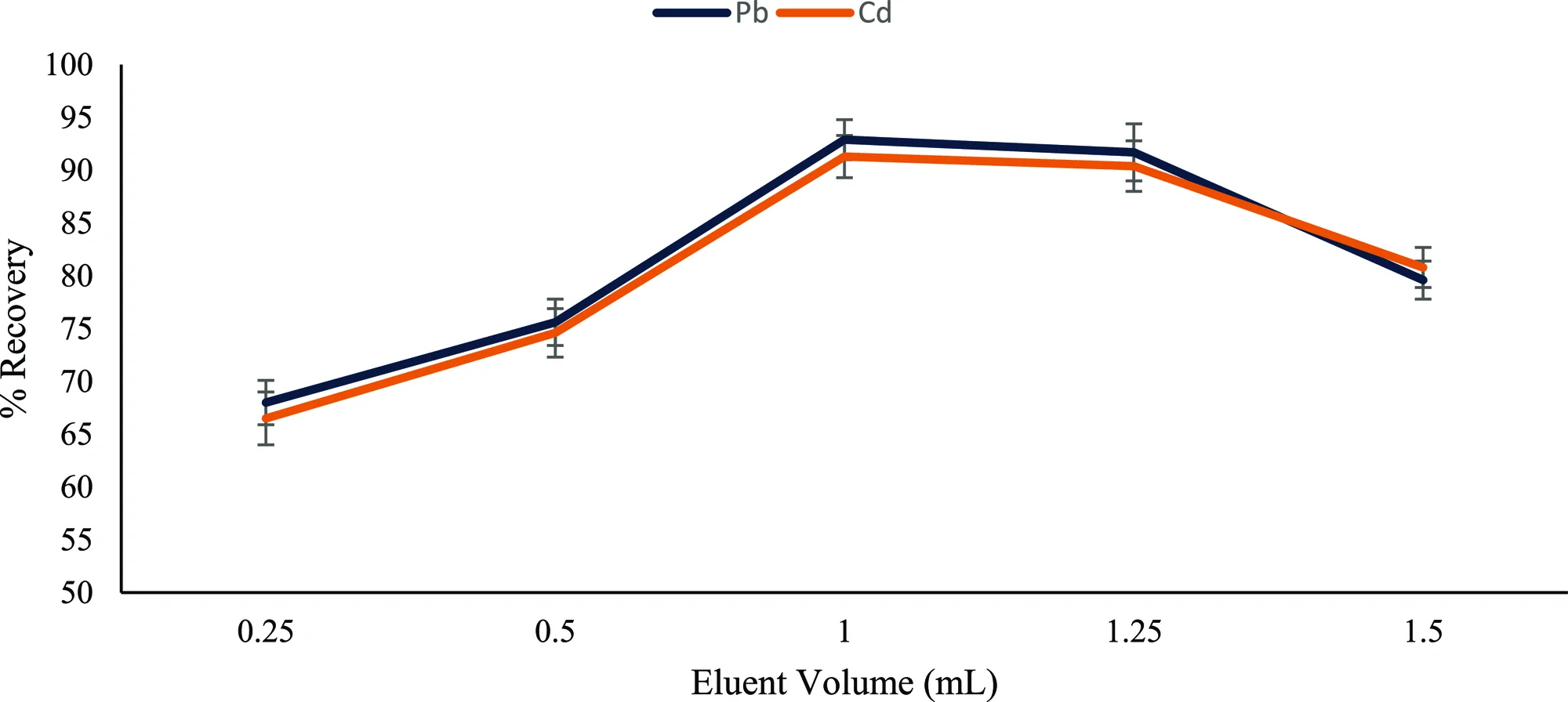
Effect of eluent volume (mL) on the recovery of Pb­(II) and Cd­(II) ions using 0.5 mol L^–1^ HNO_3_ (*n* = 3).

### Optimization of pH Conditions for Metal Extraction

The pH of the sample solution is one of the most influential parameters in UA-DMSPE procedures, as it directly affects both the speciation of metal ions in solution and the ionization state of functional groups located on the sorbent surface. Since metal retention is primarily governed by coordination interactions between analyte ions and active binding sites, careful optimization of pH is necessary to maximize extraction efficiency and analytical reliability.^11^ To evaluate the influence of solution pH, extraction experiments were performed over the pH range of 3–9. As illustrated in Figure 10, the recovery values of Pb­(II) and Cd­(II) were highly dependent on the solution pH. At strongly acidic conditions (pH 3–4), relatively low recoveries were obtained for both analytes. This behavior can be attributed to the high concentration of hydrogen ions competing with metal ions for the available coordination sites on the sorbent surface, thereby limiting effective metal binding. A pronounced increase in extraction recovery was observed with increasing pH, and the highest recoveries were achieved at pH 6, reaching approximately 94% for Pb­(II) and 89% for Cd­(II). At this pH, the active functional groups of the sorbent are expected to be sufficiently deprotonated to favor coordination with metal ions, while Pb­(II) and Cd­(II) predominantly remain in their dissolved ionic forms, allowing efficient sorbent–metal interactions. At pH 7, a slight decline in recovery was observed, whereas a substantial decrease occurred under more alkaline conditions (pH 8–9). This reduction may be attributed to changes in metal speciation and the progressive formation of hydrolyzed or insoluble hydroxide species, particularly for Pb­(II) and Cd­(II), thereby decreasing the concentration of free metal ions available for sorption.^46^ Based on the obtained results, pH 6 was selected as the optimum extraction pH for subsequent UA-DMSPE experiments.

**Figure 10:**
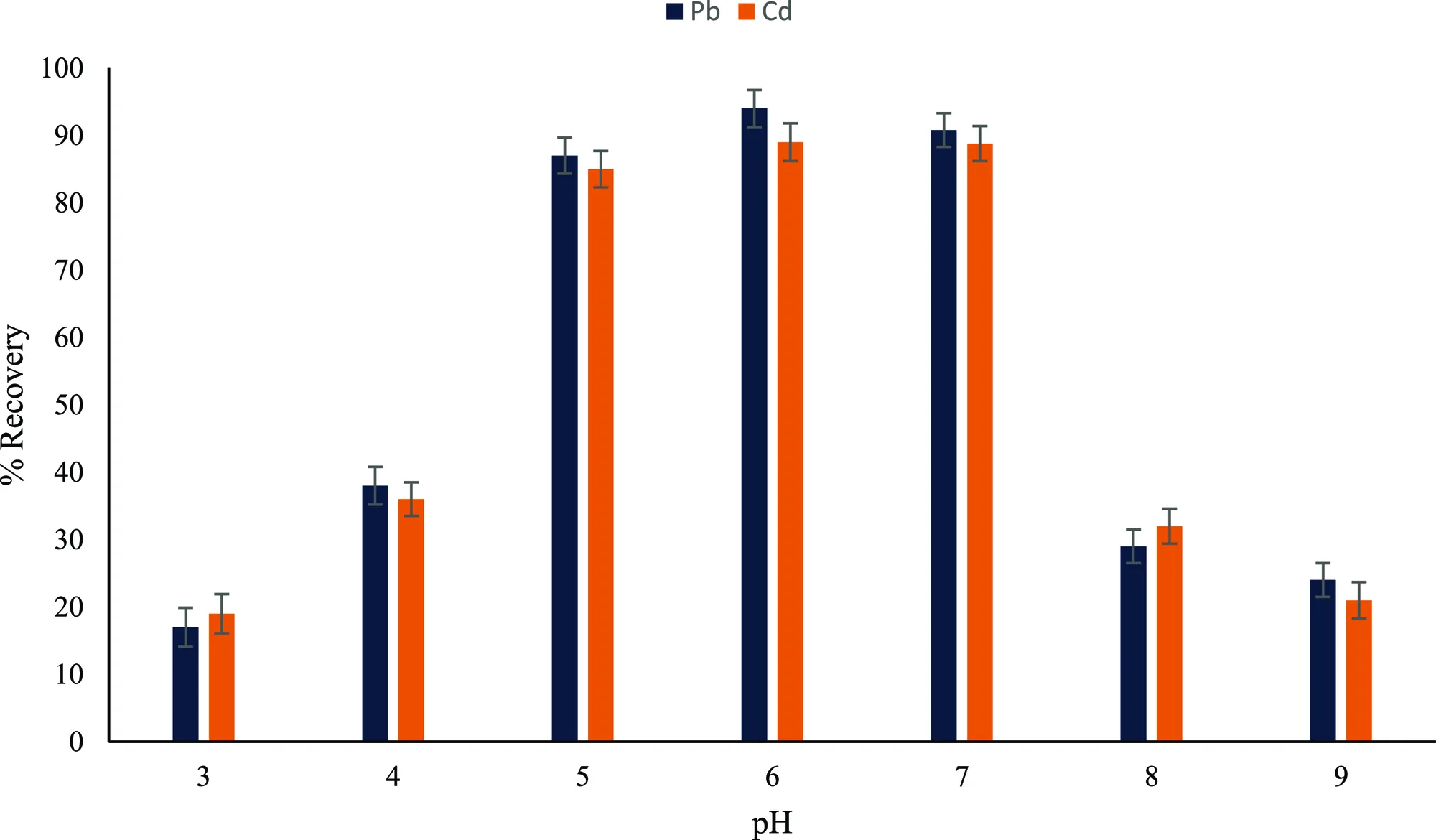
Effect of solution pH on the recovery of Pb­(II) and Cd­(II) ions in the UA-DMSPE procedure (*n* = 3).

### Effect of Sorbent Amount on Extraction Efficiency

The amount of sorbent employed in the UA-DMSPE procedure is an important factor affecting extraction performance, as it determines the total number of available adsorption and coordination sites for interaction with target metal ions. An insufficient sorbent dosage may limit metal retention due to the restricted availability of active functional groups, whereas excessive sorbent amounts may adversely influence extraction efficiency by reducing effective mass transfer and elution performance.^60^ To investigate the influence of sorbent dosage, extraction experiments were performed using 10, 20, 30, 40, 50, 60, and 70 mg of the synthesized sorbent under otherwise identical experimental conditions. As presented in Figure 11, the recovery values of Pb­(II) and Cd­(II) gradually increased with increasing sorbent amount up to 30 mg, indicating enhanced interaction between metal ions and the active functional groups available on the sorbent surface. The highest recoveries were obtained at 30 mg, suggesting that this amount provided an adequate number of accessible binding sites for efficient sorption of both analytes. However, when the sorbent amount exceeded 30 mg, a slight decrease in recovery was observed. This behavior may be associated with partial aggregation of sorbent particles at higher dosages, leading to reduced effective surface accessibility and less efficient contact between metal ions and active sites. In addition, increasing the sorbent mass may hinder quantitative desorption during the elution step, thereby negatively affecting overall extraction recovery.^61^ Therefore, 30 mg was selected as the optimum sorbent amount for all subsequent experiments.

**Figure 11:**
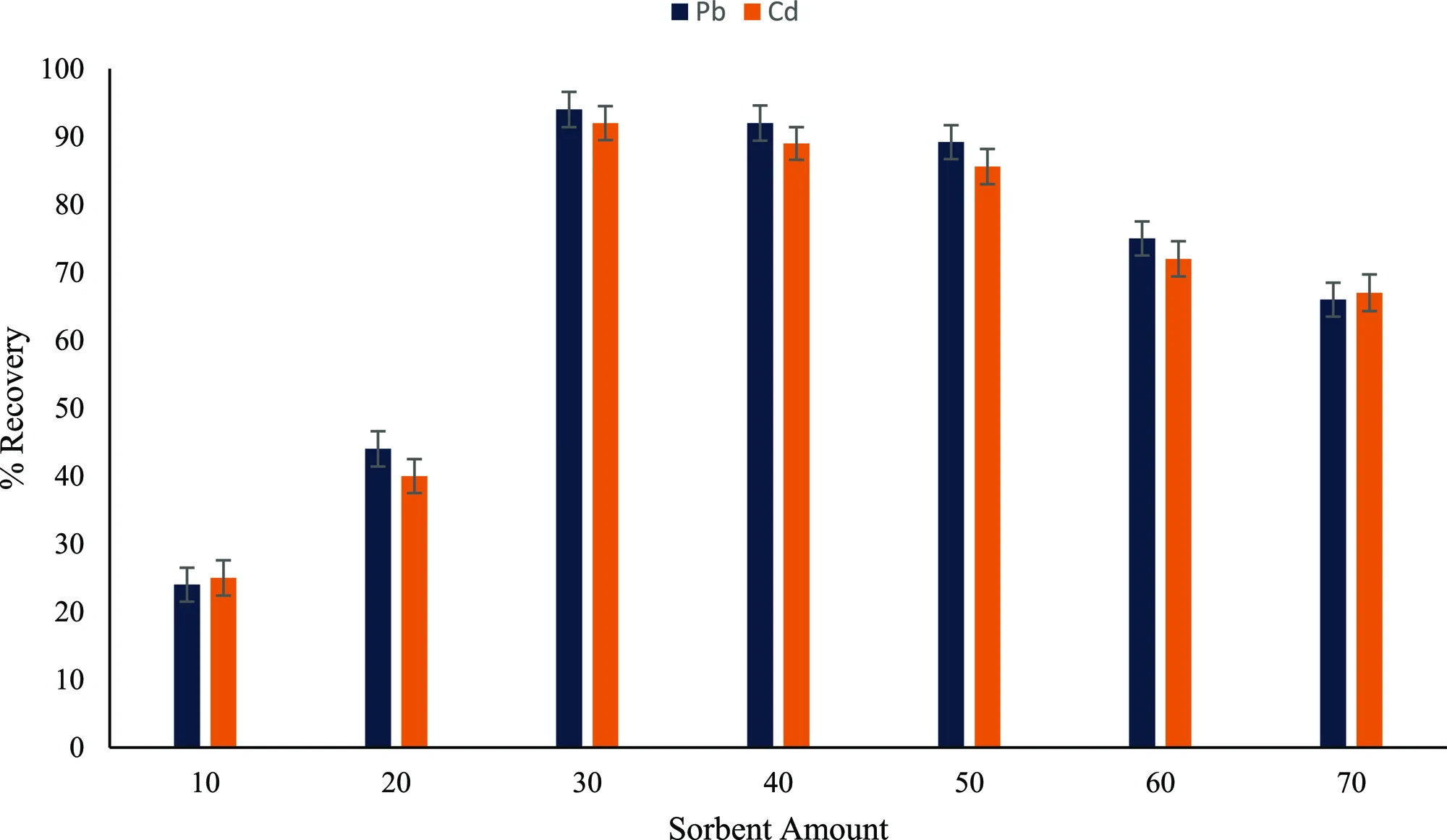
Effect of sorbent amount (mg) on the recovery of Pb­(II) and Cd­(II) ions in the UA-DMSPE procedure (*n* = 3).

### Optimization of Extraction Time

Extraction time is an important parameter in UA-DMSPE systems because it directly affects the establishment of equilibrium between dissolved metal ions and the active binding sites available on the sorbent surface. Sufficient contact time is required to ensure effective interaction between analyte ions and the sorbent; however, excessively prolonged extraction periods may negatively influence extraction efficiency due to shifts in adsorption–desorption equilibrium.^62^ To evaluate the influence of extraction time, experiments were performed over the range of 5–35 min (5, 10, 15, 20, 25, 30, and 35 min) under otherwise optimized conditions. As shown in Figure 12, the recovery values of Pb­(II) and Cd­(II) gradually increased with increasing extraction time, indicating improved interaction between metal ions and the active functional groups on the sorbent surface. Ultrasonic agitation likely promoted more homogeneous dispersion of sorbent particles throughout the solution, thereby enhancing mass transfer and facilitating more effective sorbent–metal interactions. The highest recoveries for both analytes were obtained at an extraction time of 20 min, suggesting that adsorption equilibrium was effectively reached under these conditions. However, extending the extraction time beyond 20 min resulted in a slight decrease in recovery values. This behavior may be associated with alterations in adsorption–desorption equilibrium during prolonged ultrasonic treatment, which could reduce the efficiency of metal retention on the sorbent surface. Similar observations have been reported in previous UA-DMSPE studies employing functionalized silica-based sorbents, where excessive sonication time negatively affected extraction efficiency.^63^ Considering both extraction efficiency and analytical practicality, 20 min was selected as the optimum extraction time for subsequent experiments. The relatively short equilibration period further indicates the beneficial contribution of ultrasonic assistance to improving extraction kinetics in the developed UA-DMSPE procedure.

**Figure 12:**
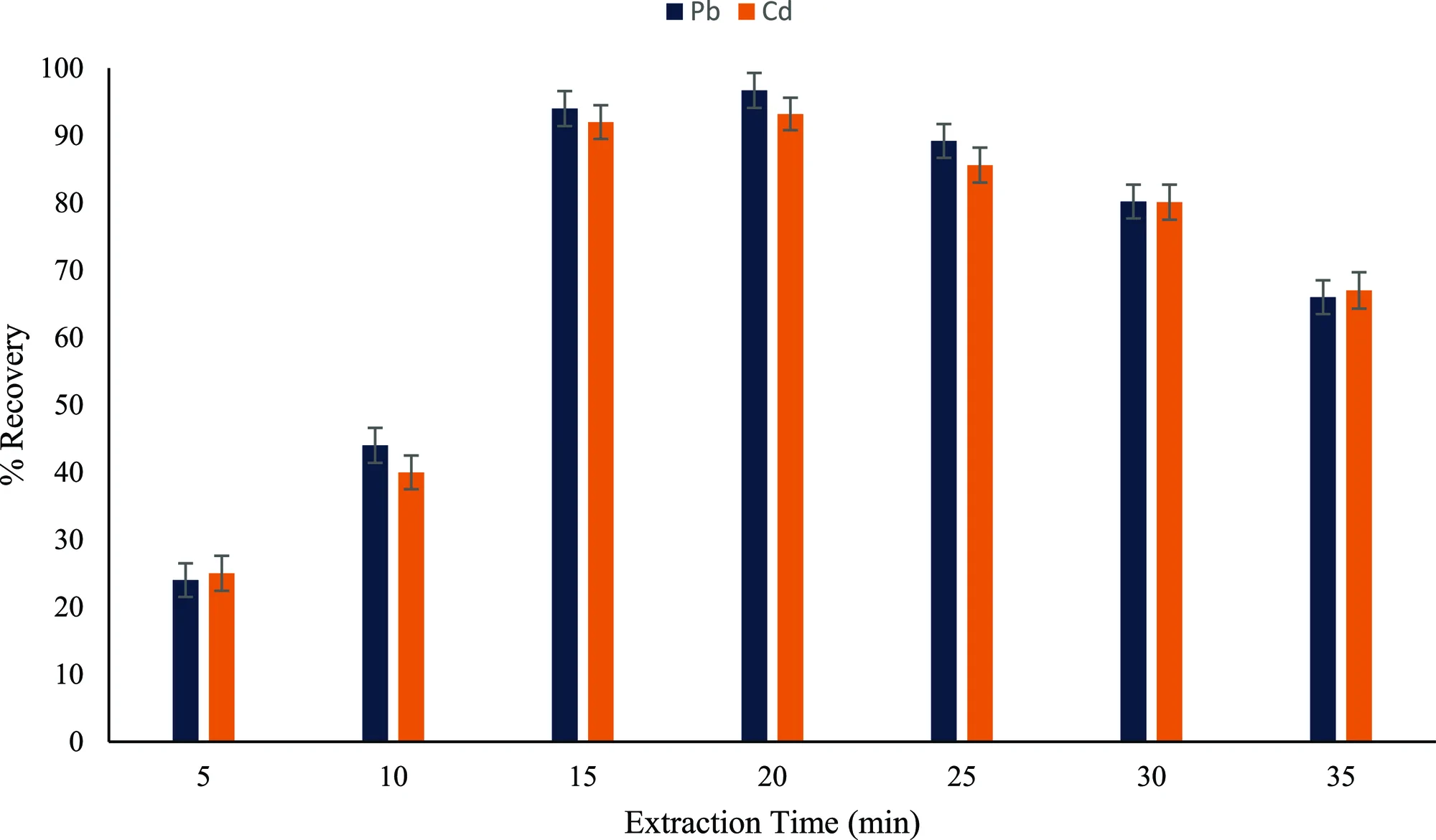
Effect of extraction time (min) on the recovery of Pb­(II) and Cd­(II) ions in the UA-DMSPE procedure (*n* = 3).

The optimized experimental conditions established for the ultrasound-assisted dispersive micro solid-phase extraction (UA-DMSPE) procedure are summarized in Table 2. Several experimental variables that may influence the extraction behavior of Pb­(II) and Cd­(II), including ligand concentration used for sorbent preparation, solution pH, sorbent dosage, extraction time, and the characteristics of the elution medium (type, concentration, and volume), were systematically investigated. Preliminary optimization was performed through a one-factor-at-a-time (OFAT) approach, in which a single variable was varied while all remaining parameters were maintained constant to evaluate its individual contribution to extraction performance. The optimum operating conditions were selected based on the highest extraction recoveries together with satisfactory reproducibility, thereby ensuring efficient metal retention and reliable analytical performance of the developed UA-DMSPE method.

### Multivariate Optimization Using Central Composite Design

Following the preliminary one-factor-at-a-time (OFAT) optimization studies, suitable experimental ranges were established for the principal variables influencing the UA-DMSPE procedure. Although the OFAT approach is useful for evaluating the individual influence of a single parameter, it does not adequately account for possible interaction effects among multiple variables and may require a large number of experimental trials. Therefore, a multivariate optimization strategy based on central composite design (CCD) coupled with response surface methodology (RSM) was employed to simultaneously evaluate the effects of experimental parameters and their interactions. This statistical approach enables the determination of optimum operating conditions with a reduced number of experiments while providing a comprehensive understanding of the extraction system (Table 3).

**Table 3:** Optimized Experimental Conditions for the UA-DMSPE Procedure Applied to Pb­(II) and Cd­(II) Determination

parameter	optimum condition
ligand concentration (for sorbent preparation)	3 × 10^–4^ mol L^–1^
sorbent amount	30 mg
sample volume	10.0 mL
sample pH	6.0
extraction time	20 min
eluent type	HNO_3_
eluent concentration	0.5 mol L^–1^ HNO_3_
eluent volume	1.0 mL

Based on the experimental ranges established through preliminary OFAT optimization, a central composite design (CCD) coupled with response surface methodology (RSM) was employed to optimize the extraction conditions of the developed UA-DMSPE procedure. Six independent variables, namely ligand concentration (A, 1 × 10^–5^–5 × 10^–4^ mol L^–1^), eluent volume (B, 0.2–2.5 mL), solution pH (C, 3–9), sorbent amount (D, 10–50 mg), extraction time (E, 5–30 min), and sample volume (F, 2.5–20 mL), were selected as process variables, while extraction recovery (%) was considered as the analytical response (Table 4). A total of 86 experimental runs, including factorial, axial, and center points, were performed to evaluate both the individual and interactive effects of the investigated variables on extraction performance. Under the studied experimental domain, the recovery values varied between approximately 19.1% and 100%, indicating a substantial influence of experimental conditions on extraction efficiency. The complete CCD experimental matrix together with the corresponding recovery values is provided in the Supporting Information (Table S1).

**Table 4:** Experimental Factors and Their Investigated Ranges Employed in the Central Composite Design (CCD)

factor	symbol	unit	low level	center point	high level
ligand concentration	A	mol L^–1^	1 × 10^–5^	2.55 × 10^–4^	5 × 10^–4^
eluent volume	B	mL	0.2	1.35	2.5
pH	C	–	3	6	9
sorbent amount	D	mg	10	30	50
extraction time	E	min	5	17.5	30
sample volume	F	mL	2.5	11.25	20

### Statistical Modeling and Analysis of Variance (ANOVA)

To identify the most appropriate mathematical model describing the relationship between the experimental variables and extraction recovery, sequential model sum of squares and analysis of variance (ANOVA) were evaluated. Among the tested models, the quadratic model was selected as the most suitable owing to its statistical significance and satisfactory fitting characteristics. The model exhibited a high F-value (39.85) with a statistically significant probability value (*p* < 0.0001), indicating that the developed response surface model adequately represented the extraction system and that the observed variation was not attributable to random noise. According to the ANOVA results, the quadratic terms (A^2^, B^2^, C^2^, D^2^, E^2^, and F^2^) were found to be statistically significant (*p* < 0.0001), suggesting the presence of curvature effects in the response surface and highlighting the importance of nonlinear relationships among the investigated variables. In contrast, the linear and interaction terms showed comparatively lower statistical significance within the studied experimental domain. Furthermore, the lack-of-fit test was statistically nonsignificant (*F* = 1.50, *p* = 0.2669), demonstrating good agreement between the experimental and predicted values and confirming the adequacy of the proposed model for response prediction. The statistical performance of the quadratic model is summarized in Table 5. The model exhibited an R^2^ of 0.9488, an adjusted R^2^ of 0.9250, and a predicted R^2^ of 0.8608, indicating excellent agreement between the experimental and predicted responses. The difference between the adjusted and predicted R^2^ values was less than 0.2, confirming satisfactory predictive capability of the model. Furthermore, the Adequate Precision value of 18.1184, which is substantially higher than the recommended minimum value of 4, demonstrates an adequate signal-to-noise ratio and confirms that the developed model is suitable for navigating the experimental design space.

**Table 5:** ANOVA Results of the Quadratic Model Obtained From CCD–RSM Optimization of the UA-DMSPE Procedure

source	sum of squares	df	mean square	F-value	p-value	
model	69577,28	27	2576,94	39,85	<0.0001	significant
A-ligand concentration	1,42	1	1,42	0,0219	0,8829	
B-eluent volume	0,1005	1	0,1005	0,0016	0,9687	
C-pH	44,34	1	44,34	0,6857	0,4110	
D-sorbent amount	22,94	1	22,94	0,3547	0,5538	
E-extraction time	3,97	1	3,97	0,0614	0,8051	
F-sample volume	0,4307	1	0,4307	0,0067	0,9352	
AB	16,50	1	16,50	0,2552	0,6153	
AC	5,58	1	5,58	0,0863	0,7700	
AD	0,3164	1	0,3164	0,0049	0,9445	
AE	0,1314	1	0,1314	0,0020	0,9642	
AF	0,5439	1	0,5439	0,0084	0,9272	
BC	49,53	1	49,53	0,7659	0,3851	
BD	68,27	1	68,27	1,06	0,3085	
BE	0,1139	1	0,1139	0,0018	0,9667	
BF	3,38	1	3,38	0,0522	0,8201	
CD	97,27	1	97,27	1,50	0,2250	
CE	0,7877	1	0,7877	0,0122	0,9125	
CF	15,90	1	15,90	0,2459	0,6219	
DE	0,2627	1	0,2627	0,0041	0,9494	
DF	11,65	1	11,65	0,1801	0,6729	
EF	1,66	1	1,66	0,0256	0,8734	
A^2^	1455,53	1	1455,53	22,51	<0.0001	
B^2^	2178,14	1	2178,14	33,68	<0.0001	
C^2^	3373,31	1	3373,31	52,16	<0.0001	
D^2^	2888,47	1	2888,47	44,67	<0.0001	
E^2^	2501,74	1	2501,74	38,69	<0.0001	
F^2^	2624,96	1	2624,96	40,59	<0.0001	
residual	3750,75	58	64,67			
lack of fit	3340,65	49	68,18	1,50	0,2669	not significant
pure error	410,10	9	45,57			
cor total	73328,03	85				

To facilitate a clearer visual comparison of the relative contribution of each model term, a standardized Pareto chart was constructed based on the ANOVA results and is presented in Figure 13. As shown, only the quadratic terms (A^2^, B^2^, C^2^, D^2^, E^2^, and F^2^) exceeded the statistical significance threshold, whereas all linear and two-factor interaction terms remained below the reference line. These findings indicate that extraction recovery was mainly influenced by the quadratic effects of the investigated variables. The negligible contribution of the interaction terms suggests that simultaneous changes in two experimental factors did not produce statistically significant synergistic or antagonistic effects within the investigated design space. These observations are consistent with the ANOVA results summarized in Table 5.

**Figure 13:**
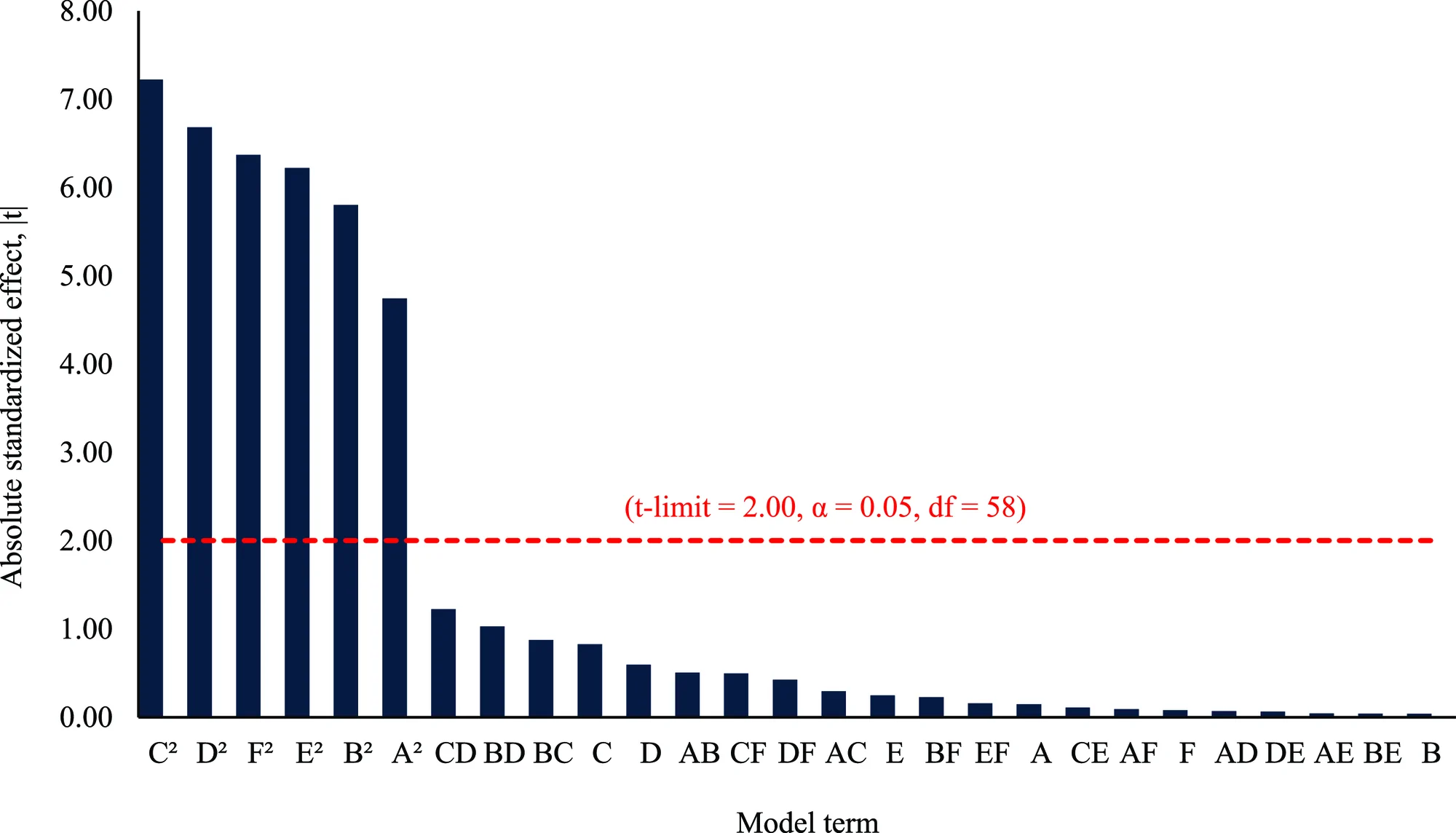
Standardized Pareto chart showing the relative effects of model terms on extraction recovery.

### Response Surface and Contour Plot Analysis

To further elucidate the combined influence of experimental variables on extraction recovery, three-dimensional (3D) response surface and contour plots were generated based on the quadratic CCD model. These graphical representations provide a visual interpretation of the response behavior within the investigated experimental domain and facilitate the evaluation of variable interactions as well as the identification of optimum operating regions. Since the quadratic terms were found to be statistically significant, the response surfaces exhibited noticeable curvature effects, indicating the presence of nonlinear relationships between the investigated variables and extraction performance. Representative response surface and contour plots illustrating the combined effects of selected variables on extraction recovery are presented in Figures 14–17.

**Figure 14:**
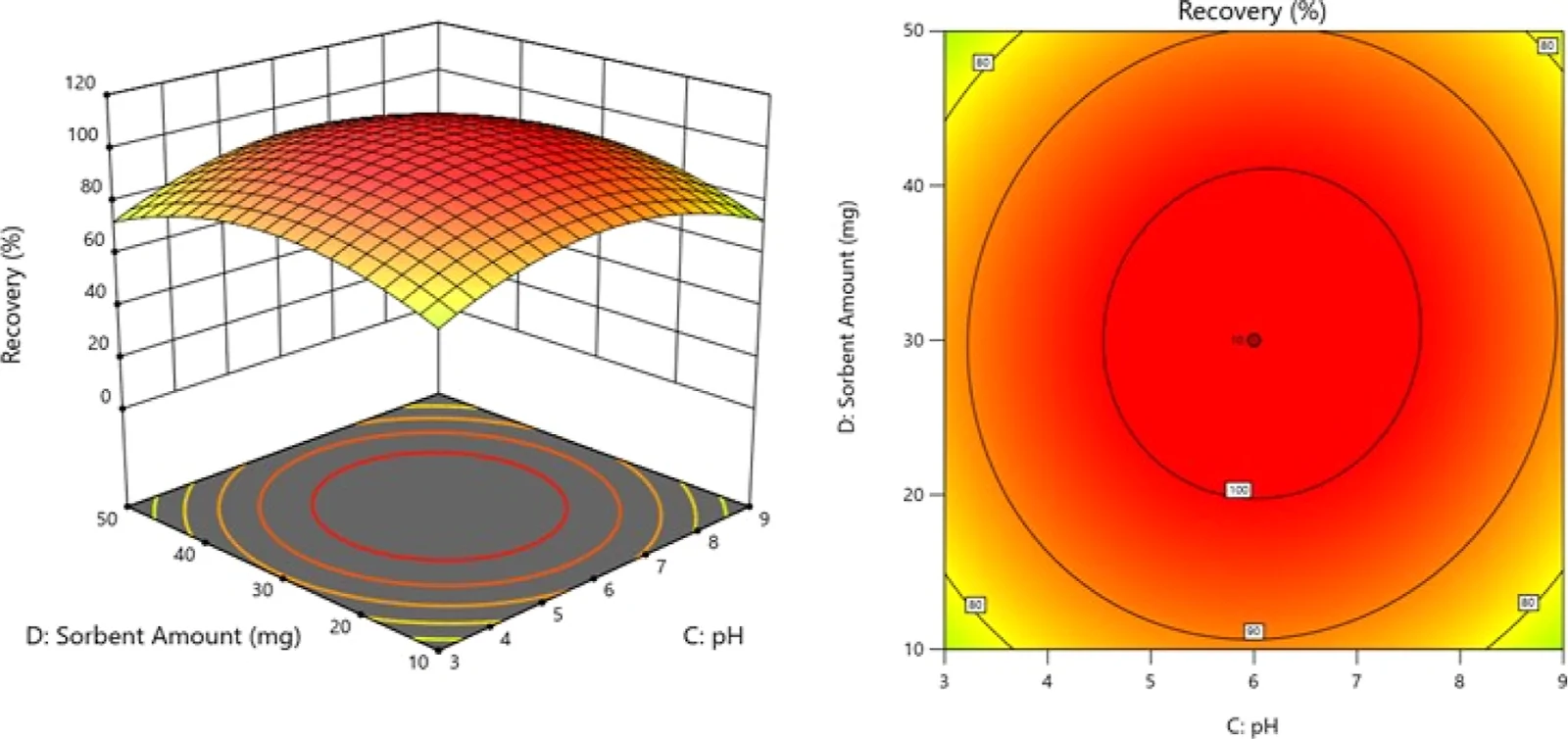
Response surface and contour plots showing the interaction between solution pH and sorbent amount on extraction recovery (%).

The combined influence of solution pH and sorbent amount on extraction recovery is illustrated by the three-dimensional response surface and contour plots shown in Figure 14. As evident from the response surface, extraction recovery was strongly dependent on both variables, with a pronounced curvature effect indicating the existence of a nonlinear relationship between pH, sorbent dosage, and extraction efficiency. At acidic conditions (pH 3–4), relatively low recoveries were observed, which can be attributed to the competition of hydrogen ions with Pb­(II) and Cd­(II) ions for the active coordination sites present on the sorbent surface. As the pH increased, extraction recovery markedly improved and reached a maximum near pH 6, where the oxygen-containing donor groups of the ligand loaded onto the silica surface are expected to provide more favorable coordination interactions with metal ions. Likewise, increasing the sorbent amount enhanced extraction recovery up to approximately 30 mg owing to the increased availability of accessible binding sites. However, no significant improvement was observed at higher sorbent dosages, suggesting that excessive sorbent loading may partially reduce extraction efficiency due to aggregation effects and restricted mass transfer. The contour plot further confirms that the optimum extraction region was concentrated around pH 6 and a sorbent amount of approximately 30 mg, which is in excellent agreement with the numerical optimization results predicted by the CCD model.

The combined influence of solution pH and extraction time on extraction recovery is illustrated in the three-dimensional response surface and contour plots shown in Figure 15. The obtained response surface exhibited a noticeable curvature, indicating a nonlinear relationship between the investigated variables and extraction performance. Extraction recovery was strongly affected by solution pH, with substantially lower recoveries observed under acidic conditions due to competition between hydrogen ions and metal ions for the active adsorption sites of the sorbent. As the pH increased, recovery values markedly improved and reached maximum levels around pH 6, indicating more favorable coordination interactions between Pb­(II)/Cd­(II) ions and the oxygen-containing donor groups of the silica-loaded ligand. In parallel, increasing extraction time enhanced recovery up to approximately 17.5–20 min, which may be attributed to improved contact between the sorbent and analyte ions and accelerated mass transfer under ultrasonic irradiation. However, no additional improvement was observed at longer extraction times, suggesting that adsorption equilibrium had already been established and that prolonged sonication may slightly disturb sorption–desorption equilibrium. The contour plot further confirmed that the optimum extraction region was concentrated around pH 6 and an extraction time of approximately 17.5 min, in good agreement with the optimum conditions predicted by the CCD model.

**Figure 15:**
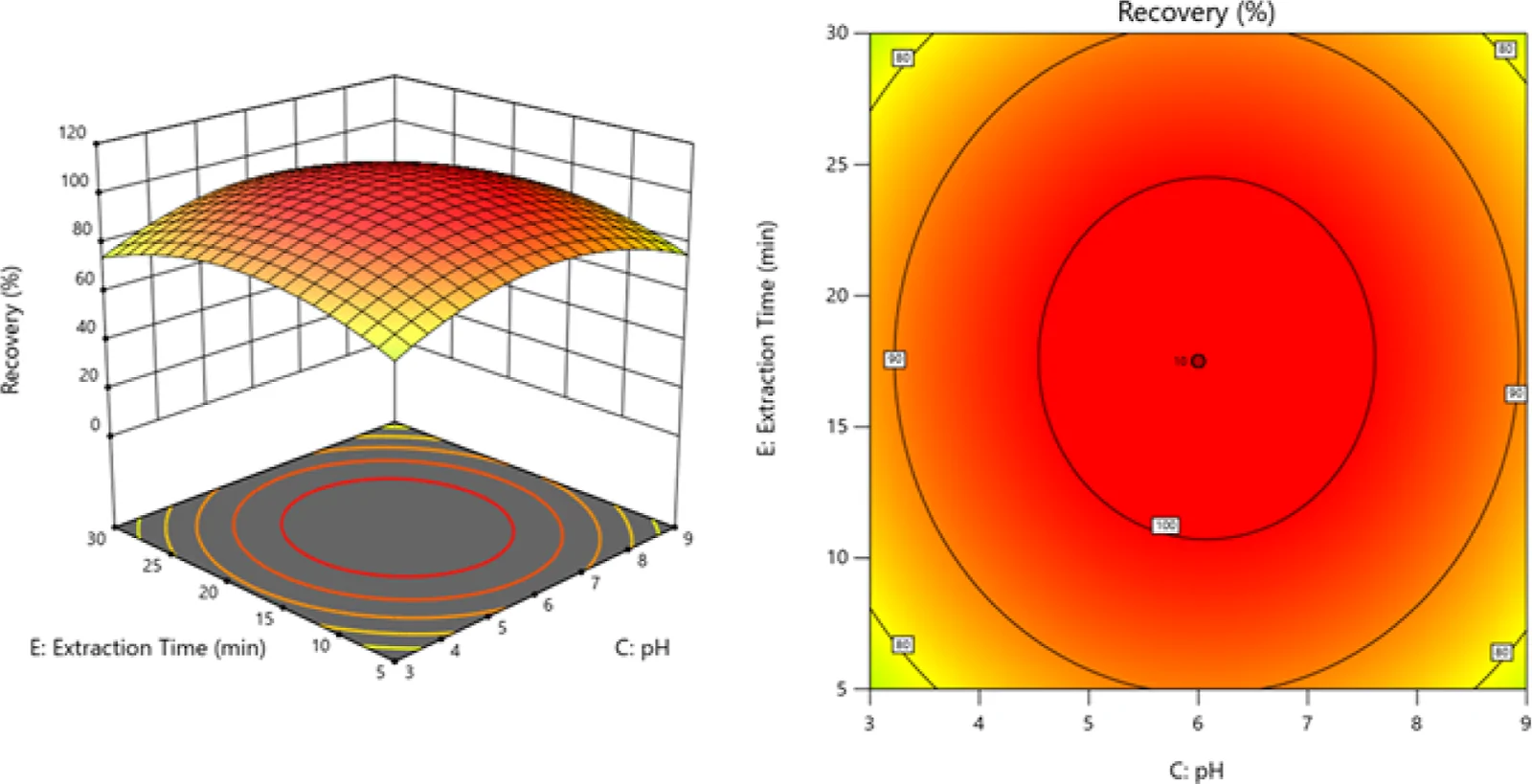
Response surface and contour plots showing the interaction between solution pH and extraction time on extraction recovery (%).

The combined influence of sorbent amount and sample volume on extraction recovery is illustrated in Figure 16. The generated response surface exhibited a distinct curvature effect, indicating a nonlinear relationship between these variables and extraction performance. Increasing the sorbent amount initially enhanced extraction recovery owing to the increased availability of active adsorption and coordination sites for Pb­(II) and Cd­(II) ions. Maximum recovery values were observed at approximately 30 mg of sorbent, suggesting that this dosage provided sufficient active binding capacity for efficient metal retention. However, further increases in sorbent amount did not result in additional improvement and slightly reduced recovery, possibly due to particle aggregation and reduced accessibility of active sites. Likewise, sample volume strongly affected extraction efficiency. At lower sample volumes, efficient interaction between analyte ions and sorbent active sites promoted high recoveries, whereas excessive sample volumes led to a slight decline in extraction efficiency, which may be associated with dilution effects and insufficient sorbent capacity relative to the total analyte load. The contour plot demonstrated that the optimum operational region was centered around approximately 30 mg sorbent amount and 11.25 mL sample volume, which is consistent with the optimum conditions predicted by the CCD model.

**Figure 16:**
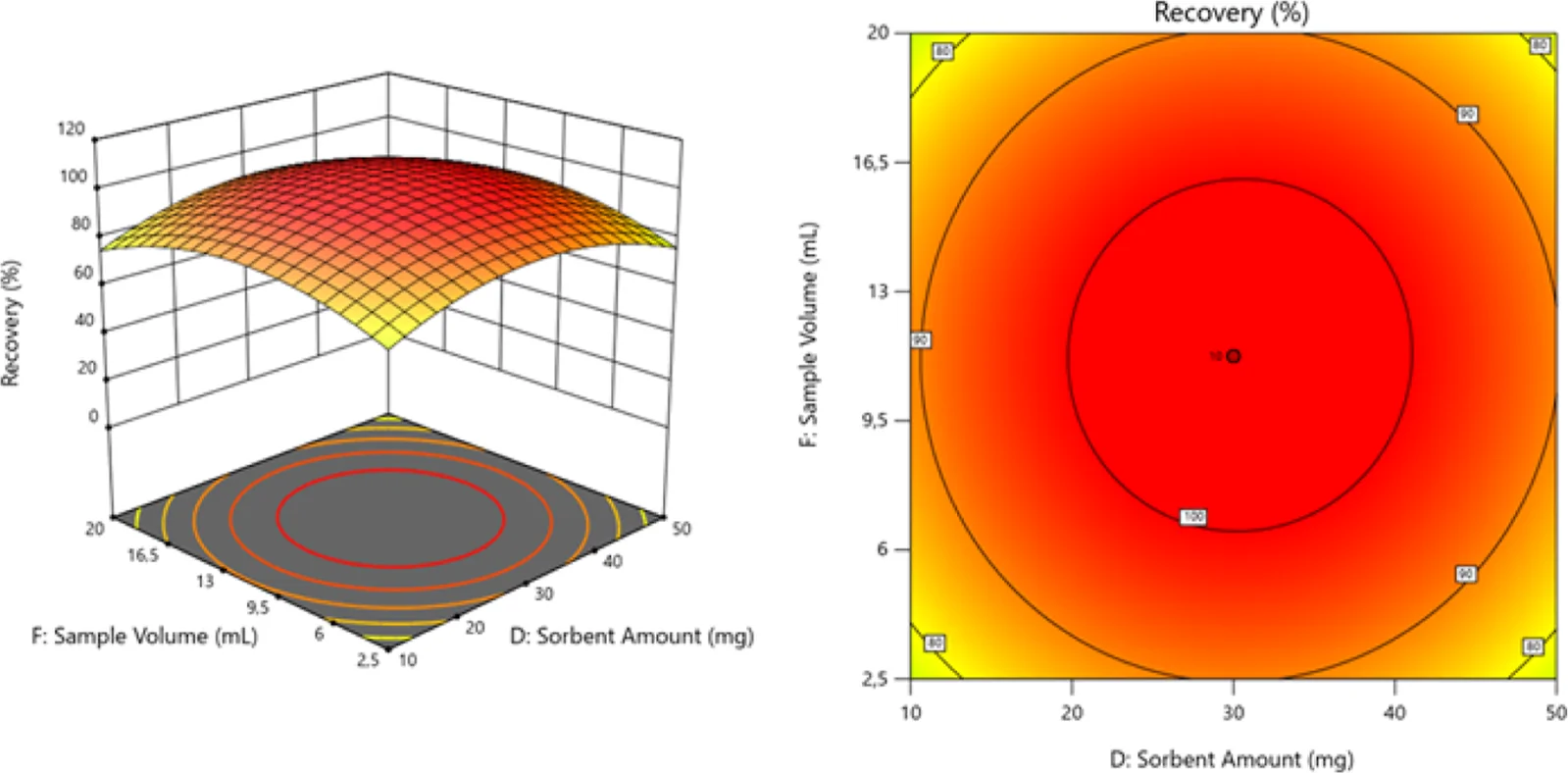
Three-dimensional response surface and contour plots illustrating the combined effect of sorbent amount and sample volume on extraction recovery (%) in the UA-DMSPE procedure.

The combined influence of ligand concentration and eluent volume on extraction recovery is illustrated in Figure 17 The obtained response surface exhibited a characteristic curvature pattern, indicating the existence of nonlinear relationships between these experimental variables and extraction performance. Increasing ligand concentration initially enhanced extraction recovery, which may be attributed to the greater availability of active coordination sites on the sorbent surface resulting from increased ligand loading. Maximum recovery values were obtained at approximately 2.55 × 10^–4^ mol L^–1^, suggesting that sufficient ligand loading was achieved for efficient interaction between Pb­(II)/Cd­(II) ions and the sorbent surface. However, further increases in ligand concentration did not improve recovery and slightly reduced extraction performance, which may be associated with excessive surface coverage and steric hindrance effects limiting accessibility of active sites. Similarly, eluent volume significantly affected extraction efficiency. Increasing eluent volume improved desorption of retained analytes up to an optimum value of approximately 1.35 mL, whereas excessive eluent volumes resulted in a slight decline in recovery, most likely due to dilution effects reducing analyte concentration in the final solution. The contour plot further confirmed that the optimum operational region was centered around a ligand concentration of approximately 2.55 × 10^–4^ mol L^–1^ and an eluent volume of 1.35 mL, in agreement with the optimum conditions predicted by the CCD model.

**Figure 17:**
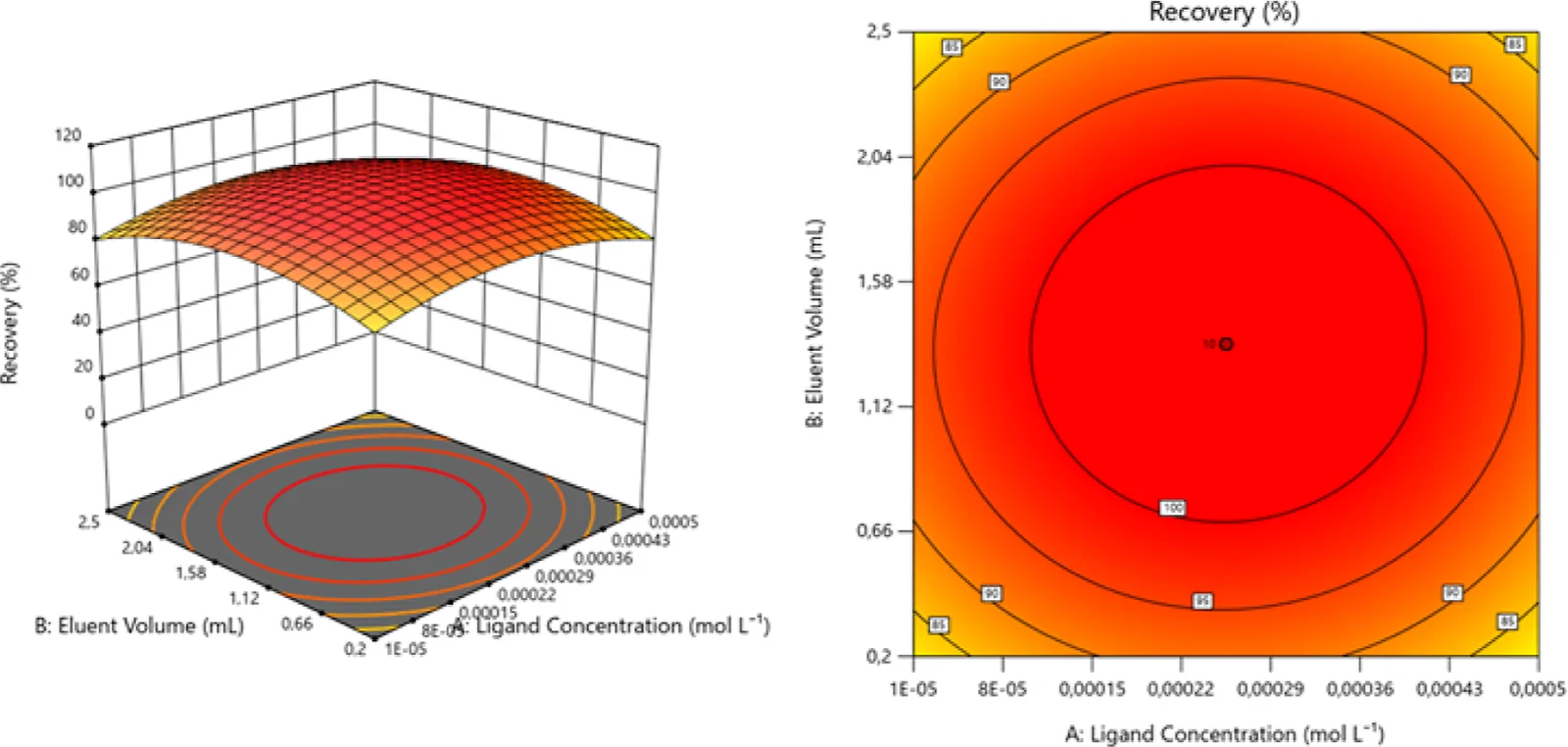
Three-dimensional response surface and contour plots illustrating the combined effect of ligand concentration and eluent volume on extraction recovery (%) in the UA-DMSPE procedure.

As summarized in Table 6, the optimum extraction conditions predicted by the CCD model were found to be in close agreement with the preliminary conditions established through one-factor-at-a-time (OFAT) optimization. Minor variations observed between the investigated CCD ranges and the experimentally selected values did not significantly affect extraction performance. Therefore, the final UA-DMSPE procedure was established using pH 6.0, 30 mg sorbent amount, 20 min extraction time, 10.0 mL sample volume, and elution with 1.0 mL of 0.5 mol L^–1^ HNO_3_. The consistency between the OFAT and CCD results further confirms the reliability and robustness of the developed extraction protocol.

**Table 6:** Comparison of Optimum Extraction Conditions Obtained by OFAT and CCD Optimization Approaches

parameter	OFAT optimum	CCD investigated range	CCD-confirmed/final condition	comment
ligand concentration	3 × 10^–4^ mol L^–1^	1 × 10^–5^–5 × 10^–4^ mol L^–1^	3 × 10^–4^ mol L^–1^	confirmed as suitable
eluent volume	1.0 mL	0.2–2.5 mL	1.0 mL	provided efficient desorption
pH	6.0	3–9	6.0	maximum recovery region
sorbent amount	30 mg	10–50 mg	30 mg	confirmed optimum
extraction time	20 min	5–30 min	20 min	sufficient equilibrium time
sample volume	10.0 mL	2.5–20 mL	10.0 mL	selected as practical optimum
eluent concentration	0.5 mol L^–1^ HNO_3_		0.5 mol L^–1^ HNO_3_	final eluent condition

### Numerical Optimization and Validation of the CCD Model

Numerical optimization based on the desirability function approach was performed to determine the optimum extraction conditions for achieving maximum recovery of Pb­(II) and Cd­(II) ions. The optimization process aimed to maximize extraction recovery while maintaining all experimental variables within the investigated design space. As shown in Figure 18, the optimum extraction conditions predicted by the CCD model were a ligand concentration of 2.98 × 10^–4^ mol L^–1^, an eluent volume of 1.22 mL, solution pH of 6.66, sorbent amount of 35.10 mg, extraction time of 21.68 min, and sample volume of 13.26 mL. Although these values represent the numerical optimum predicted by the response surface model, slight adjustments were made to obtain experimentally practical operating conditions. Therefore, the experimental conditions were set to 3 × 10^–4^ mol L^–1^ ligand concentration, 1.0 mL eluent volume, pH 6.0, 30 mg sorbent, 20 min extraction time, and 10 mL sample volume. These conditions remained within the predicted optimum region and provided comparable extraction recoveries while improving experimental simplicity, reproducibility, and routine applicability. Under these conditions, the model predicted an extraction recovery of 100.22% with an overall desirability value of 1.000, indicating excellent agreement between the selected experimental conditions and the optimization objective. To verify the predictive capability and reliability of the developed quadratic model, validation experiments were performed under the predicted optimum conditions. The experimentally obtained recovery values showed close agreement with the model-predicted response, confirming the adequacy and robustness of the CCD–RSM optimization approach for describing the extraction behavior of the developed UA-DMSPE system. The high desirability value together with the agreement between predicted and experimental recoveries further demonstrated the suitability of the proposed optimization strategy for maximizing extraction performance.

**Figure 18:**
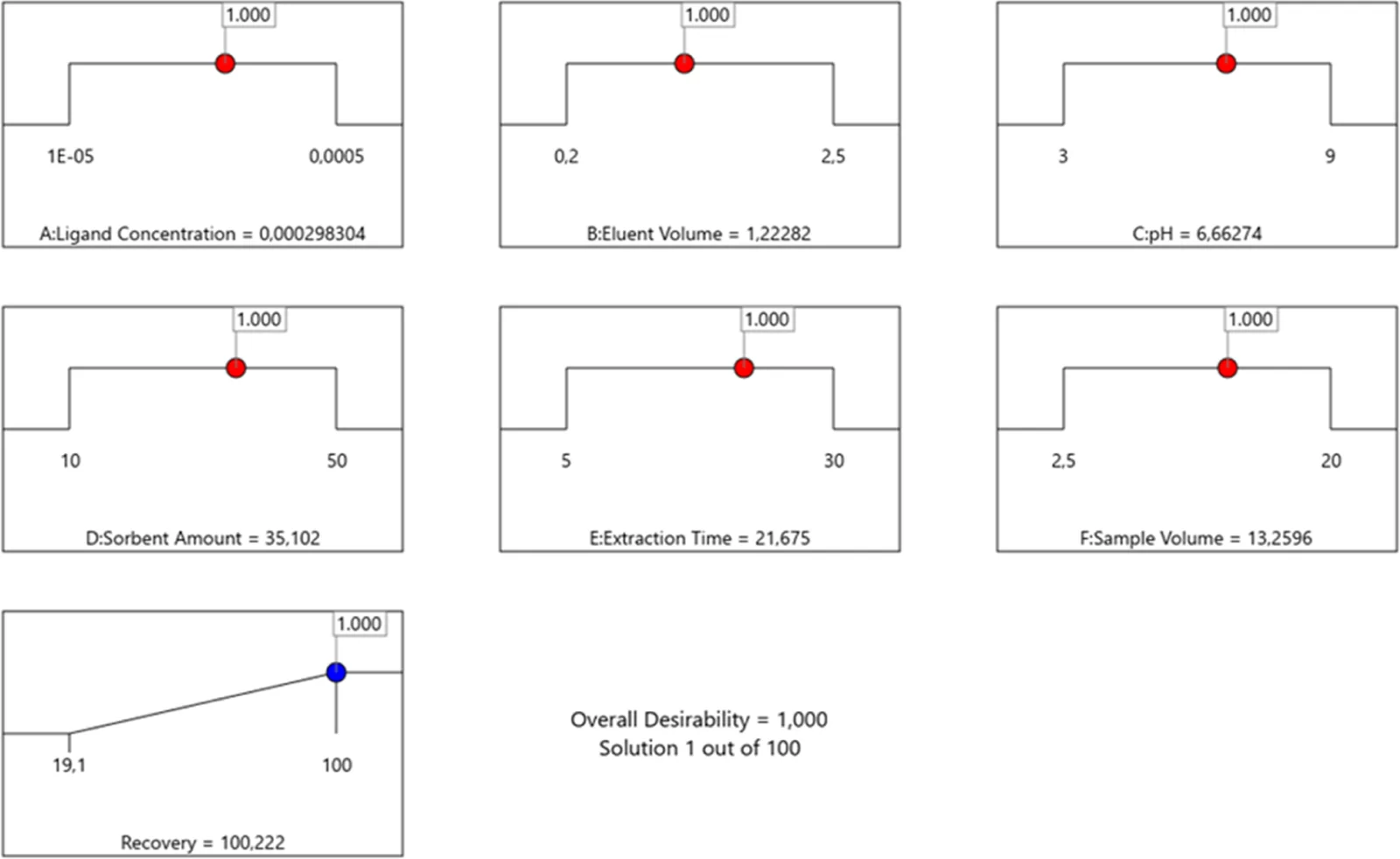
Numerical optimization results obtained using the desirability function approach for the CCD–RSM model applied to the UA-DMSPE procedure.

### Comparative Evaluation of the Synthesized Sorbents Under Optimized Conditions

Following the optimization studies, the extraction performances of the three synthesized anthraquinone-derived sorbents (Derivative 1–SiO_2_, Derivative 2–SiO_2_, and Derivative 3–SiO_2_) were comparatively evaluated under the optimized UA-DMSPE conditions to identify the most efficient sorbent for Pb­(II) and Cd­(II) retention in rice and paddy soil matrices. Since the three derivatives possess the same molecular framework and donor atom composition but differ in the positional arrangement of functional groups, a comparative evaluation was performed to assess the effect of positional isomerism on extraction performance and to experimentally validate the DFT-assisted ligand selection. As presented in Figure 19, all sorbents exhibited appreciable extraction capability toward Pb­(II); however, noticeable differences in recovery performance were observed. Derivative 2 provided the highest recovery values, reaching approximately 99% for rice and 98% for paddy soil samples, whereas lower recoveries were obtained using Derivative 1 (approximately 87–89%) and Derivative 3 (approximately 81–83%). Likewise, as illustrated in Figure 20, a similar trend was observed for Cd­(II), where Derivative 2 again demonstrated superior extraction performance (approximately 95–97%), followed by Derivative 1 and Derivative 3. Overall, the extraction efficiency followed the order: Derivative 2 > Derivative 1 > Derivative 3. The observed differences may be attributed to variations in donor atom orientation and steric accessibility arising from positional isomerism, which may influence the effectiveness of metal–ligand coordination. Importantly, the experimentally observed extraction trend was in good agreement with the DFT calculations, supporting the validity of the DFT-assisted ligand screening approach and confirming Derivative 2 as the most suitable sorbent for subsequent analytical applications.

**Figure 19:**
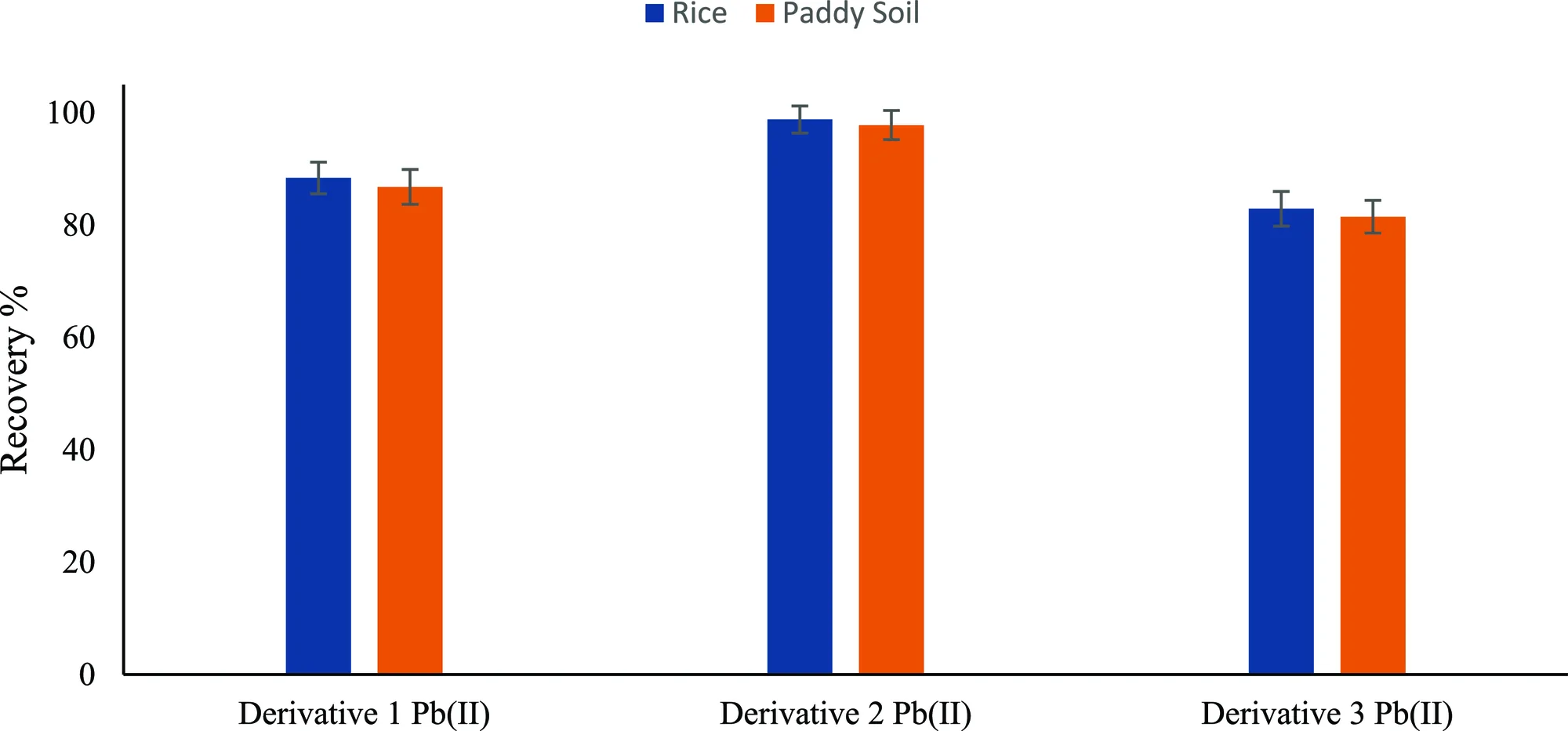
Recovery performance of Derivatives 1–3 for Pb­(II) determination in rice and paddy soil samples under optimized UA-DMSPE conditions (*n* = 3).

**Figure 20:**
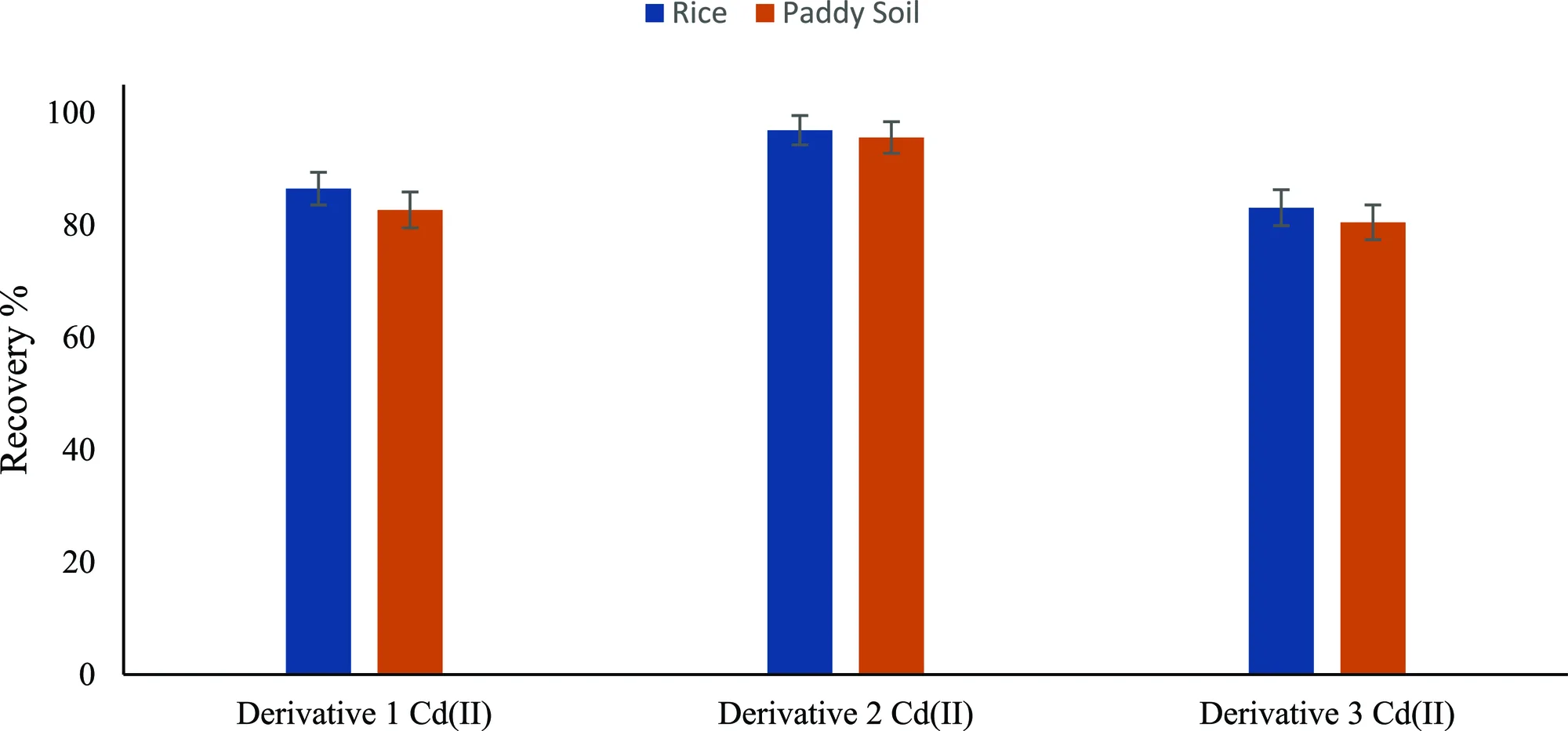
Recovery performance of Derivatives 1–3 for Cd­(II) determination in rice and paddy soil samples under optimized UA-DMSPE conditions (*n* = 3).

### Green Analytical Assessment of the Proposed UA-DMSPE Method

In recent years, the development of environmentally responsible analytical methodologies has become an essential consideration in modern analytical chemistry, with increasing attention directed toward minimizing chemical consumption, reducing hazardous waste generation, and improving overall methodological sustainability.^14^ In this context, the environmental impact of the proposed ultrasound-assisted dispersive micro solid-phase extraction (UA-DMSPE) procedure was systematically assessed to determine its compliance with the principles of green analytical chemistry. The developed method incorporates several environmentally favorable characteristics, including reduced reagent and solvent consumption, low sorbent dosage, relatively short extraction time, and minimal waste generation compared with conventional extraction techniques. Furthermore, the use of a microscale extraction format combined with efficient preconcentration capability contributes to lowering the environmental footprint of the analytical procedure while maintaining satisfactory analytical performance. Therefore, the greenness profile of the developed method was comprehensively evaluated using contemporary green analytical assessment tools, namely the Analytical Eco-Scale, Green Analytical Procedure Index (GAPI), and Analytical GREEnness (AGREE) metrics, which provide complementary information regarding the sustainability and environmental compatibility of the proposed methodology.

### Analytical Eco-Scale Assessment

The environmental performance of the optimized UA-DMSPE procedure was evaluated using the Analytical Eco-Scale according to the literature-established approach. Penalty points were assigned based on reagent hazard, energy consumption, waste generation, and occupational safety. The use of a low volume of aqueous nitric acid for elution (1.0 mL of 0.5 mol L^–1^ HNO_3_) together with only dropwise addition of dilute acid/base for pH adjustment resulted in 5 penalty points for reagent hazard. Energy consumption associated with ultrasound-assisted extraction (20 min), centrifugation, and FAAS detection contributed an additional 5 points. Waste generation was limited to approximately 10 mL aqueous sample phase and 1 mL acidic eluate per run, yielding only 2 penalty points, while no organic solvent waste was produced.^64^ No additional hazards beyond routine laboratory handling of dilute acidic solutions were identified; therefore, no penalty points were assigned for occupational safety. Overall, the method accumulated 12 penalty points, corresponding to an Eco-Scale score of 88, classifying the proposed procedure as an Excellent Green Analysis. These results demonstrate the environmentally favorable character of the developed method due to its low reagent consumption, minimal waste generation, and relatively low energy demand (Table 7).

**Table 7:** Analytical Eco-scale Evaluation of the Optimized UA-DMSPE Procedure for Pb­(II) and Cd­(II) Determination

category	experimental description (optimized conditions)	penalty points	remarks
reagent hazard	elution with 1.0 mL of 0.5 mol L^–1^ HNO_3_ (aqueous). pH adjustment at pH 6.0 using dilute HNO_3_/NaOH (dropwise). No organic solvent employed	5	low-volume aqueous acid; solvent-free operation and reduced chemical consumption
energy consumption	ultrasound-assisted extraction (20 min, room temperature); centrifugation (5000 rpm, 10 min × 2); FAAS measurement	5	moderate sonication and centrifugation time; low-energy instrumental detection (FAAS)
waste generation	approximately 10.0 mL aqueous sample phase discarded per run +1.0 mL acidic eluate after measurement (total ≈11 mL aqueous waste/run). No organic waste generated	2	waste volume mainly originates from sample phase; acidic eluate is limited and can be neutralized prior to disposal
occupational safety	standard PPE and fume hood required for handling dilute nitric acid; no volatile or toxic organic solvents used	0	no significant additional hazards beyond routine handling of dilute acidic solutions

### Green Analytical Procedure Index (GAPI) Assessment of the Proposed Method

The environmental performance of the optimized UA-DMSPE procedure was evaluated using the Green Analytical Procedure Index (GAPI). This approach assesses the greenness of an analytical method throughout all stages of the analytical workflow, ranging from sample collection and preparation to instrumental determination and waste management, by assigning a three-level color code: green (□), yellow (□), and red (●). Green indicates low environmental impact, yellow represents moderate environmental burden, generally associated with limited use of hazardous reagents or moderate energy demand, while red denotes high environmental impact arising from toxic solvents, excessive reagent consumption, or energy-intensive instrumentation.^65^ As shown in Table 8, the developed UA-DMSPE method exhibited a predominantly green GAPI profile, with only a few stages classified as yellow (□). The favorable environmental performance of the method can be attributed to the use of a low sample volume (10 mL), solvent-free operation, low reagent consumption (1.0 mL of 0.5 mol L^–1^ HNO_3_ for elution), simple aqueous-based sample preparation, and limited waste generation. Minor environmental burdens were associated with the use of dilute nitric acid, ultrasound-assisted extraction, and FAAS instrumentation, which require moderate energy input compared to greener alternatives. Nevertheless, the overall GAPI evaluation confirmed that the proposed UA-DMSPE procedure represents an environmentally friendly analytical approach consistent with the principles of green analytical chemistry.

**Table 8:** GAPI Assessment of the Proposed UA-DMSPE–FAAS Method

category	assessment (applied to current procedure)	color	remarks
1. sample collection	rice and paddy soil sampling; 10.0 mL extract analyzed	□	low sample volume
2. sample preservation/storage	short-term storage without extensive preservatives	□	minimal treatment
3. sample preparation	pH adjustment to 6.0 and UA-DMSPE	□	simple preparation
4. sample amount	10.0 mL per experiment	□	microscale analysis
5. reagent type and amount	1.0 mL of 0.5 mol L^–1^ HNO_3_ as eluent	□	acid used, but at low volume
6. solvent use	no organic solvent used	□	major environmental advantage
7. derivatization/chemical modification	none	□	no additional reagents
8. extraction/enrichment method	UA-DMSPE (20 min ultrasound-assisted extraction)	□	moderate energy consumption
9. instrumentation	FAAS	□	lower energy demand than ICP-based techniques
10. calibration method	external aqueous calibration	□	simple and low waste
11. analytical performance	high recovery and low RSD values	□	reliable analytical performance
12. waste generation	∼10.0 mL aqueous phase +1.0 mL acidic eluate	□	limited waste volume
13. waste treatment	neutralizable acidic aqueous waste	□	easy disposal
14. operator safety	PPE and fume hood required	□	nitric acid requires precaution
15. occupational exposure	minimal	□	low risk
16. overall environmental impact	low reagent consumption, solvent-free operation, reusable sorbent	□	highly green analytical method

### AGREE-Based Greenness Assessment of the Proposed Method

The greenness of the optimized UA-DMSPE procedure was evaluated using the Analytical GREEnness (AGREE) approach based on the 12 principles of green analytical chemistry. According to the AGREE assessment, the developed method achieved an overall score of approximately 0.84, indicating a high level of environmental sustainability.^15^ High individual scores were obtained for the absence of derivatization (1.00), low reagent and solvent consumption (0.95), minimal sample size and operational steps (0.95), and waste minimization (0.90). These favorable scores were mainly attributed to the use of a low sample volume (10.0 mL), solvent-free operation, and the consumption of only 1.0 mL of 0.5 mol L^–1^ aqueous nitric acid during elution. In addition, simple pH adjustment (pH 6.0) prior to extraction and limited waste generation further supported the green profile of the method. Moderate score reductions were associated with laboratory-based FAAS detection instead of in situ analysis (0.70), manual operation without automation (0.70), moderate energy consumption related to ultrasound-assisted extraction and centrifugation (0.75), and sequential rather than simultaneous multielement determination (0.75). Overall, the AGREE evaluation confirmed that the proposed UA-DMSPE method is well aligned with the principles of green analytical chemistry, combining efficient analytical performance with low reagent consumption, limited waste generation, and safe operating conditions (Table 9).

**Table 9:** AGREE Assessment of the Developed UA-DMSPE–FAAS Method According to the 12 Principles of Green Analytical Chemistry

no	green analytical chemistry principle	description (applied to current method)	score (0–1)
1	direct analytical techniques preferred	preconcentration by UA-DMSPE is required before FAAS determination of trace Pb(II) and Cd(II)	0.80
2	minimal sample size and number of steps	only 10.0 mL sample volume; simple adsorption–elution procedure with limited operational steps	0.95
3	in-situ measurements if possible	laboratory-based FAAS system; not portable for field analysis	0.70
4	minimal sample preparation	simple pH adjustment (pH 6.0) prior to extraction; limited preparation for rice and paddy soil extracts	0.85
5	automation and miniaturization	manual batch UA-DMSPE operation; miniaturized but not automated	0.70
6	avoid derivatization	no derivatization or additional chemical modification required	1.00
7	low reagent and solvent consumption	1.0 mL of 0.5 mol L^–1^ HNO_3_ used for elution; no organic solvent involved	0.95
8	nonhazardous reagents	mainly aqueous media; dilute HNO_3_ and NaOH used only in small amounts for pH adjustment and elution	0.85
9	energy efficiency	20 min ultrasound-assisted extraction, centrifugation, and FAAS measurement require moderate energy input	0.75
10	waste minimization and treatment	approximately 10.0 mL aqueous phase +1.0 mL acidic eluate generated; easily neutralizable waste	0.90
11	multianalyte or multiparameter capability	Pb(II) and Cd(II) determined sequentially by FAAS rather than simultaneous multielement detection	0.75
12	operator safety and ergonomic design	standard PPE and fume hood sufficient; no toxic organic solvents used	0.95

Compared with previously reported microextraction approaches for heavy metal determination, including dispersive liquid–liquid microextraction (DLLME), ionic liquid-based DLLME (IL-DLLME), cloud point extraction (CPE), and conventional solid-phase extraction (SPE), the proposed UA-DMSPE method exhibits favorable environmental characteristics. Many previously reported methods rely on organic solvents, higher reagent consumption, or relatively longer extraction procedures, which may increase environmental burden. In contrast, the present method was developed as a solvent-free procedure requiring only a low volume of aqueous nitric acid (1.0 mL of 0.5 mol L^–1^ HNO_3_) for elution and a relatively short ultrasound-assisted extraction time. The reduced chemical consumption, limited waste generation, and simplified operational steps positively contributed to the greenness profile of the method, as reflected by the high Analytical Eco-Scale score, predominantly green GAPI profile, and favorable AGREE assessment obtained in this study.^66,67^

### Selectivity and Interference Studies

The selectivity and robustness of the proposed UA-DMSPE method were evaluated by examining the influence of potentially interfering ions commonly encountered in rice and paddy soil matrices. For this purpose, representative alkali and alkaline earth metal ions (Na^+^, K^+^, Mg^2+^, and Ca^2+^), transition metal ions (Fe^3+^, Al^3+^, Mn^2+^, Cr^3+^, Zn^2+^, Co^2+^, and Ni^2+^), and major inorganic anions (Cl^–^, NO_3_^–^, SO_4_^2–^, PO_4_^3–^, and HCO_3_^–^) were individually added to solutions containing Pb­(II) and Cd­(II) under the optimized extraction conditions. The tolerance limit was defined as the maximum concentration of the coexisting ion causing less than ±5% deviation in analyte recovery. As summarized in Table 10, the developed method exhibited good tolerance toward major matrix constituents. Alkali and alkaline earth metal ions, as well as common inorganic anions, caused negligible interference even at relatively high concentrations, with recovery values remaining within the range of 95.8–97.8% for both Pb­(II) and Cd­(II). This behavior suggests that nonspecific ionic interactions had limited influence on the extraction process and that the sorption mechanism was predominantly governed by selective coordination between the target metal ions and the functional groups of the ligand loaded onto the silica surface. Transition metal ions exhibited comparatively lower tolerance limits, particularly Fe^3+^, Zn^2+^, Co^2+^, and Ni^2+^, which may be attributed to competitive interactions for active coordination sites due to similar chemical characteristics. Nevertheless, even in the presence of these potentially competing ions, the recoveries of Pb­(II) and Cd­(II) remained within an acceptable range (94.5–96.8%), while relative standard deviations were generally below ±4%, demonstrating satisfactory precision and method stability. Overall, the interference study confirmed the high selectivity and robustness of the proposed UA-DMSPE method, indicating its suitability for reliable determination of Pb­(II) and Cd­(II) in complex rice and paddy soil matrices.

**Table 10:** Effect of Coexisting Ions on the Recovery of Pb­(II) and Cd­(II) Under the Optimized UA-DMSPE Conditions

interfering ion	source salt	tolerance limit (μg mL^–1^)	Pb(II) recovery (%)	Cd(II) recovery (%)
Na^+^	NaCl	1000	96.8 ± 2.4	97.5 ± 1.8
K^+^	KCl	1000	96.1 ± 2.7	96.9 ± 2.0
Mg^2+^	Mg(NO_3_)_2_	500	95.9 ± 2.6	96.7 ± 1.9
Ca^2+^	CaCl_2_	500	96.0 ± 2.4	96.8 ± 2.1
Fe^3+^	FeCl_3_	90	94.8 ± 3.8	95.6 ± 3.0
Al^3+^	Al(NO_3_)_3_	50	95.1 ± 3.4	96.0 ± 2.6
Mn^2+^	MnCl_2_	100	95.6 ± 2.8	96.4 ± 2.2
Cr^3+^	Cr(NO_3_)_3_	100	95.0 ± 3.5	95.8 ± 2.9
Zn^2+^	Zn(NO_3_)_2_	50	95.4 ± 3.7	97.1 ± 2.4
Co^2+^	Co(NO_3_)_2_	80	94.5 ± 3.4	96.2 ± 2.7
Ni^2+^	NiCl_2_	90	95.2 ± 3.1	96.6 ± 2.3
Cl^–^	NaCl	1000	96.3 ± 2.5	97.2 ± 1.9
NO_3_^–^	NaNO_3_	1000	96.8 ± 2.0	97.6 ± 1.5
SO_4_^2–^	Na_2_SO_4_	1000	97.0 ± 1.8	97.8 ± 1.3
PO_4_^3–^	Na_3_PO_4_	1000	96.0 ± 2.6	96.8 ± 2.0
HCO_3_^–^	NaHCO_3_	1000	96.4 ± 2.2	97.0 ± 1.7

### Effect of Sample Volume on Extraction Efficiency

The effect of sample volume on extraction efficiency was investigated over the range of 2.5–20.0 mL. As shown in Figure 21, quantitative recoveries were achieved for both Pb­(II) and Cd­(II) throughout the investigated range, with recovery values remaining above 92%. The extraction efficiency increased slightly from 2.5 to 5.0 mL and remained nearly constant between 5.0 and 15.0 mL, indicating sufficient interaction between the target metal ions and the available active binding sites of the sorbent. A slight decrease in recovery was observed at 20.0 mL, which may be associated with partial saturation of the sorbent surface and reduced availability of binding sites under higher sample loading conditions. Although larger sample volumes can theoretically improve the preconcentration factor, a sample volume of 10.0 mL was selected as the optimum condition. This choice was made as a deliberate compromise between analytical performance and method sustainability. Under the selected conditions, quantitative recoveries were maintained while minimizing sample handling time, reagent consumption, and waste generation. Moreover, the use of a lower sample volume contributed to improved method stability and operational simplicity, while remaining fully compatible with the principles of green analytical chemistry. Therefore, despite providing a moderate preconcentration factor, the selected sample volume ensured sufficient sensitivity for reliable FAAS determination of Pb­(II) and Cd­(II), together with favorable environmental performance as confirmed by the Eco-Scale, GAPI, and AGREE assessments.

**Figure 21:**
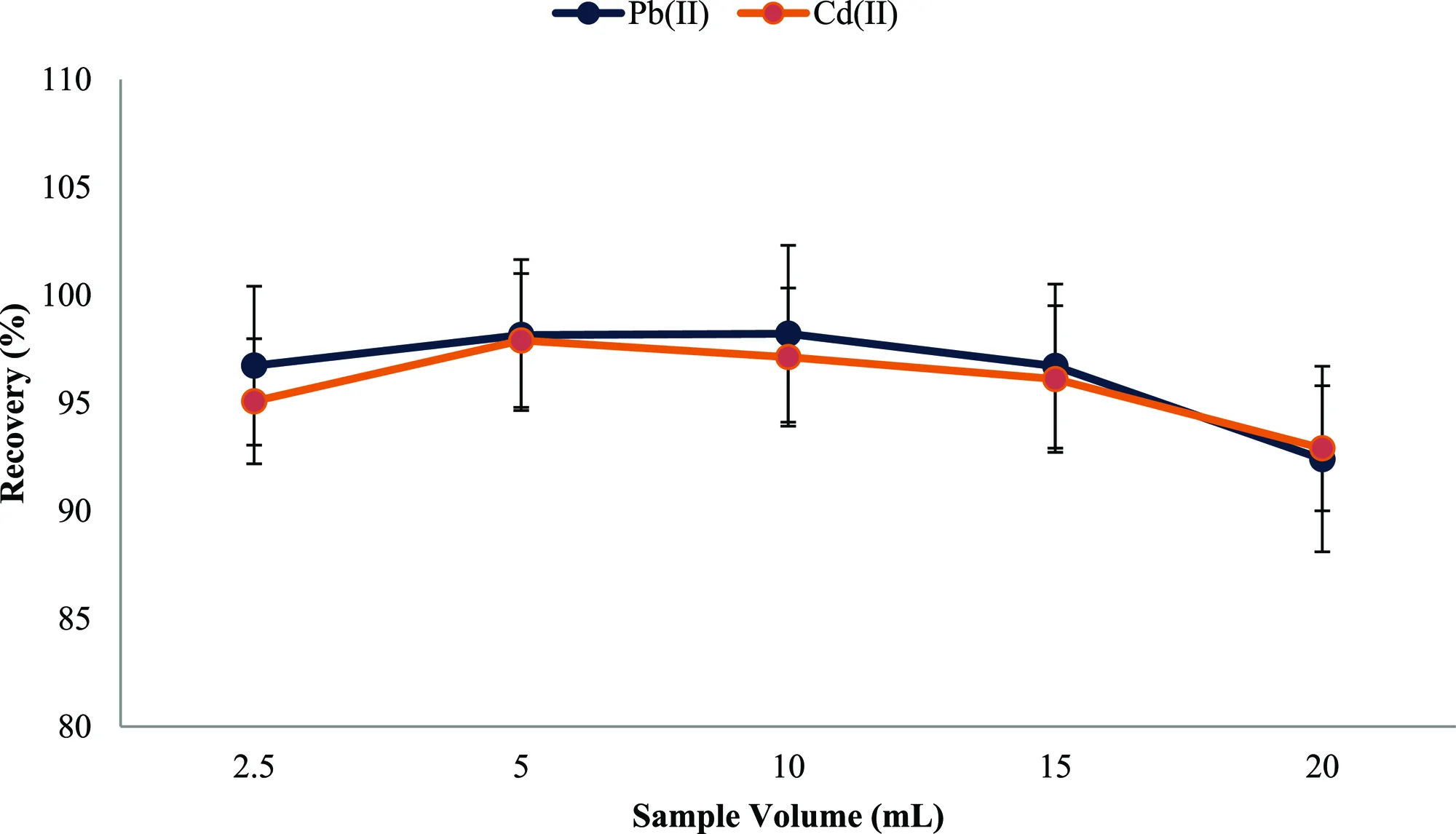
Effect of sample volume on the extraction recovery of Pb­(II) and Cd­(II) under the optimized UA-DMSPE conditions (*n* = 3).

### Evaluation of Sorbent Reusability

The reusability of the developed sorbent was evaluated through six consecutive adsorption–desorption cycles under the optimized UA-DMSPE conditions for Pb­(II) and Cd­(II). As presented in Figure 22, the sorbent exhibited high extraction efficiency during the initial cycle, with recovery values of 98.5% for Pb­(II) and 97.0% for Cd­(II), confirming the strong affinity of the Derivative 2-loaded silica sorbent toward the target analytes. During repeated use, only a gradual and limited decrease in recovery was observed, indicating good regeneration capability and operational stability of the sorbent. After five consecutive extraction cycles, recovery values remained relatively high, ranging from 93.7–98.5% for Pb­(II) and 92.4–97.0% for Cd­(II), demonstrating that the sorbent retained most of its extraction performance during repeated applications. The relatively stable recoveries obtained over the first five cycles suggest that the noncovalent interactions responsible for ligand immobilization were sufficiently stable under the applied extraction and regeneration conditions. The slight reduction in recovery may be attributed to the combined effects of partial occupation of active binding sites and incomplete desorption of metal ions during repeated acid elution. In addition, because the ligand was immobilized on the silica surface through noncovalent interactions rather than covalent grafting, repeated exposure to acidic elution and ultrasonic treatment may have caused limited weakening of the ligand–silica interactions or minor ligand loss from the surface. A more noticeable decrease was observed in the sixth cycle, where recoveries decreased to 86.0% for Pb­(II) and 85.5% for Cd­(II), suggesting a gradual reduction in sorption efficiency after prolonged reuse. Although ligand leaching was not directly quantified in the present study, this mechanism may plausibly contribute to the observed decrease in recovery after repeated regeneration cycles. Overall, the results indicate that the developed sorbent can be reused effectively for at least five consecutive extraction cycles without substantial loss of analytical performance. The observed reusability further enhances the practical applicability and environmental sustainability of the proposed method by reducing sorbent consumption and minimizing analytical waste generation.

**Figure 22:**
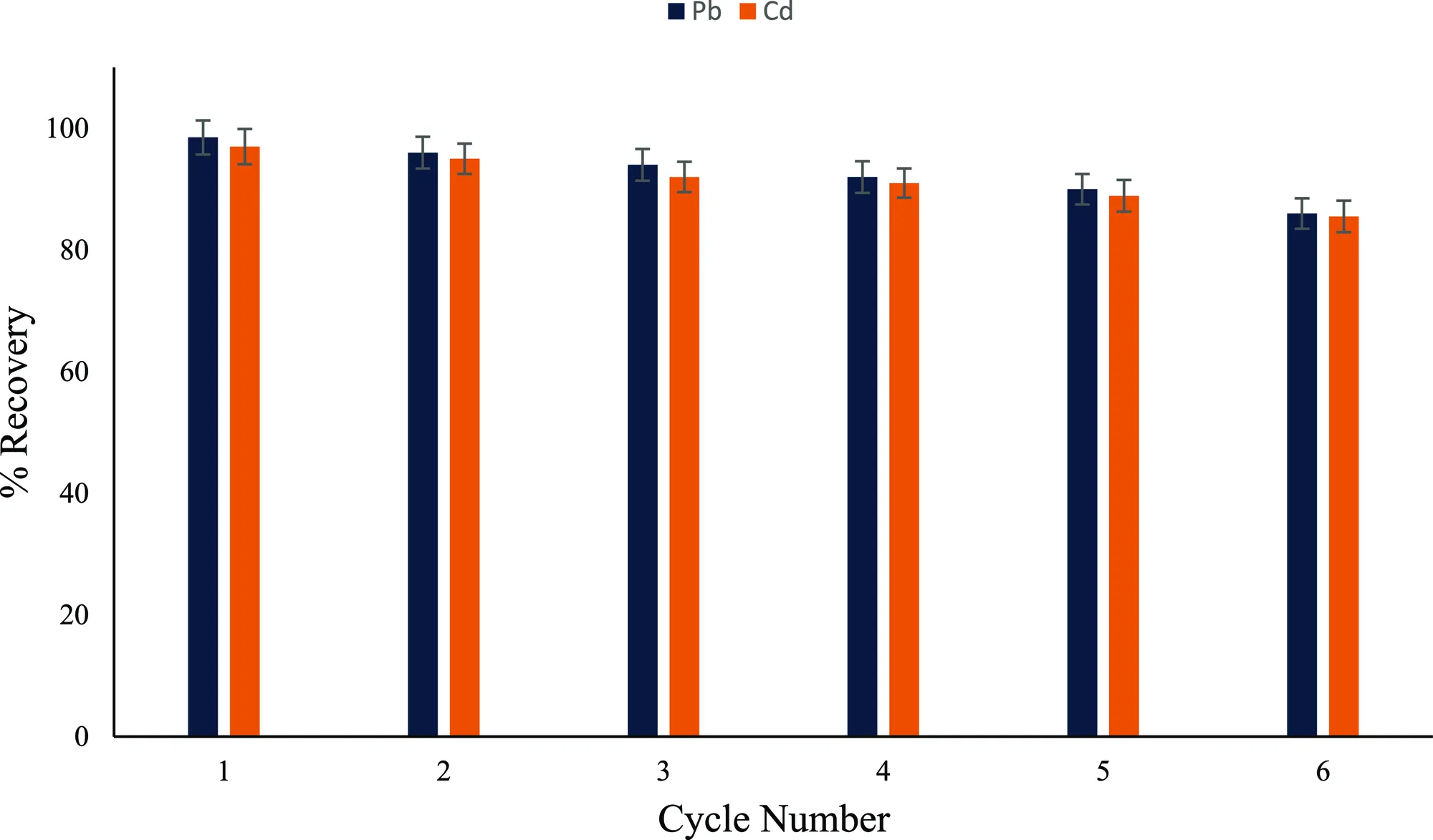
Effect of repeated adsorption–desorption cycles on the recovery of Pb­(II) and Cd­(II) (*n* = 3).

### Method Validation and Analytical Performance

The analytical performance of the proposed UA-DMSPE–FAAS method was systematically evaluated in terms of linearity, sensitivity, and precision for the determination of Pb­(II) and Cd­(II). As summarized in Table 11, satisfactory linear relationships were obtained over the concentration ranges of 0.8–500 μg L^–1^ for Pb­(II) and 1.0–500 μg L^–1^ for Cd­(II), with correlation coefficients (R^2^) of 0.9987 and 0.9983, respectively, demonstrating excellent linearity across a broad concentration interval. The sensitivity of the developed method was assessed by calculating the limits of detection (LOD) and limits of quantification (LOQ). The obtained LOD values were 0.19 μg L^–1^ for Pb­(II) and 0.28 μg L^–1^ for Cd­(II), while the corresponding LOQ values were determined as 0.63 and 0.93 μg L^–1^, respectively. The low detection limits obtained indicate that the proposed method possesses sufficient sensitivity for the determination of trace levels of Pb­(II) and Cd­(II) in rice and paddy soil samples. Furthermore, the LOQ values were found to be approximately three times higher than the corresponding LOD values, supporting the consistency and reliability of the sensitivity calculations. Method precision was evaluated in terms of intraday and interday repeatability at two concentration levels (10 and 50 μg L^–1^), expressed as relative standard deviation (%RSD). The intraday precision values ranged between 1.2–1.7%, while interday %RSD values varied from 1.7–2.2%, indicating satisfactory repeatability and reproducibility of the proposed procedure. Overall, the validation results demonstrate that the developed UA-DMSPE–FAAS method provides high sensitivity, excellent linearity, and reliable precision for the determination of trace Pb­(II) and Cd­(II) ions.

**Table 11:** Analytical Performance Characteristics of the Proposed UA-DMSPE–FAAS Method for the Determination of Pb­(II) and Cd­(II)

validation parameter	Pb(II)	Cd(II)
linear range (μg L^–1^)	0.8–500	1.0–500
R^2^ (*n* = 5)	0.9987	0.9983
LOD (μg L^–1^, *n* = 10)	0.19	0.28
LOQ (μg L^–1^, *n* = 10)	0.63	0.93
intraday precision (10 μg L^–1^, %RSD, *n* = 7)	1.5	1.7
intraday precision (50 μg L^–1^, %RSD, *n* = 7)	1.2	1.4
interday precision (10 μg L^–1^, %RSD, *n* = 7)	2.0	2.2
interday precision (50 μg L^–1^, %RSD, *n* = 7)	1.7	1.9

### Real Sample Analysis and Matrix Effect Evaluation

The applicability of the proposed UA-DMSPE–FAAS method to complex matrices was evaluated by investigating matrix effects in rice and paddy soil samples spiked with Pb­(II) and Cd­(II) at three concentration levels (10, 25, and 50 μg L^–1^). Prior to the spiking experiments, the original rice and paddy soil samples were analyzed. However, the concentrations of Pb­(II) and Cd­(II) in all collected samples were below the method detection limits (LODs); therefore, no quantifiable concentrations could be reported. Consequently, spiked recovery experiments were performed to evaluate the applicability, accuracy, and analytical performance of the proposed method in these matrices. As presented in Table 12, satisfactory recovery values were obtained for both analytes in all tested matrices, ranging between 96.3–97.4% for rice samples and 94.9–96.2% for paddy soil samples. The slightly lower recoveries observed for paddy soil may be attributed to the more complex matrix composition and the presence of naturally occurring inorganic constituents capable of competing with the target metal ions during extraction. Matrix effect assessment revealed only minor signal suppression, with percentage differences (%Diff) ranging from −2.89% to −3.65% for rice and from −3.95% to −4.62% for paddy soil samples. The negative % Diff values indicate a slight suppressive effect of the sample matrix on the analytical signal; however, the observed suppression remained limited. According to commonly accepted criteria, matrix effect values below ±10% are generally considered negligible, indicating minimal matrix interference. In the present study, all obtained % Diff values remained well below this threshold, confirming that matrix-induced effects were effectively controlled under the optimized extraction conditions. Moreover, although slightly higher standard deviation values were observed for paddy soil samples, this behavior is consistent with the expected heterogeneity of soil matrices rather than methodological instability. Overall, the results confirm that the proposed UA-DMSPE method possesses good matrix tolerance and provides reliable quantification of Pb­(II) and Cd­(II) in both rice and paddy soil samples.

**Table 12:** Application of the Proposed UA-DMSPE–FAAS Method to Rice and Paddy Soil Samples

Matrix	metal ion	spiked (μg L^–1^)	measured (μg L^–1^ ± SD)	recovery (%)	matrix effect (μg L^–1^ ± SD)	% diff
Rice	Pb(II)	10	9.74 ± 1.42	97.4	10.05 ± 1.48	–3.08
		25	24.28 ± 1.71	97.1	25.20 ± 1.65	–3.65
		50	48.65 ± 1.94	97.3	50.35 ± 1.88	–3.38
	Cd(II)	10	9.63 ± 1.56	96.3	9.95 ± 1.49	–3.22
		25	24.18 ± 1.84	96.7	24.90 ± 1.79	–2.89
		50	48.35 ± 2.02	96.7	49.95 ± 1.96	–3.20
paddy soil	Pb(II)	10	9.49 ± 1.88	94.9	9.95 ± 1.82	–4.62
		25	24.02 ± 2.14	96.1	25.10 ± 2.05	–4.30
		50	47.72 ± 2.36	95.4	49.80 ± 2.28	–4.18
	Cd(II)	10	9.53 ± 1.92	95.3	9.98 ± 1.86	–4.51
		25	24.06 ± 2.21	96.2	25.05 ± 2.14	–3.95
		50	47.80 ± 2.43	95.6	49.85 ± 2.35	–4.11

### Comparison of the Proposed Method with Previously Reported Methods

As summarized in Table 13, most previously reported extraction and preconcentration methods for heavy metal determination have primarily been applied to relatively simple matrices, including milk, mineral water, swimming pool water, tea, or other aqueous samples. Although these methods generally provide satisfactory analytical performance, their applicability to highly complex environmental and food-related matrices is often limited. In contrast, the proposed UA-DMSPE–FAAS method was successfully applied to rice and paddy soil samples, which represent considerably more challenging matrices due to their heterogeneous composition, elevated inorganic content, organic matter, silicate minerals, and potential metal-binding constituents that may interfere with extraction efficiency and instrumental determination. Despite these matrix-related challenges, the developed method exhibited satisfactory matrix tolerance and robustness, yielding high and reproducible recovery values ranging from 94.9 to 97.4% for Pb­(II) and Cd­(II). Moreover, the observed matrix effect remained minimal, confirming that the developed extraction strategy effectively minimized interference from coexisting species in both agricultural and soil matrices. Compared with previously reported FAAS- and GF-AAS-based methods, the proposed approach provides a considerably wider linear dynamic range (0.8–500 μg L^–1^ for Pb­(II) and 1.0–500 μg L^–1^ for Cd­(II)), allowing reliable determination over a broader concentration interval. In addition, the method achieved competitive detection limits (0.19 μg L^–1^ for Pb­(II) and 0.28 μg L^–1^ for Cd­(II)) using conventional FAAS instrumentation, which is more accessible and cost-effective than advanced techniques frequently employed in the literature. Although some previously reported methods provide higher enrichment factors or slightly lower detection limits, these advantages are often achieved through more complicated extraction procedures, specialized sorbents, or more sophisticated instrumental systems. In contrast, the present UA-DMSPE method combines satisfactory sensitivity, rapid extraction, operational simplicity, and low reagent consumption while maintaining reliable analytical performance in difficult agricultural matrices. Therefore, the major strength of the developed method lies not only in its analytical capability but also in its successful applicability to complex real samples such as rice and paddy soil, highlighting its practical potential for routine monitoring of toxic metals in agricultural environments.

**Table 13:** Comparison of the Proposed UA-DMSPE–FAAS Method With Reported Extraction Methods in the Literature

method	detection	metal	linear range (μg/L)	LOD (μg/L)	LOQ (μg/L)	preconcentration factor	real samples	recovery (%)	reference
UA-DMSPE	FAAS	Pb(II), Cd(II)	Pb(II): 0.8–500	Pb(II): 0.19 Cd(II): 0.28	Pb(II): 0.63 Cd(II): 0.93	10	rice and paddy Soil	94.9–97.4	this study
			Cd(II): 1.0–500						
SoAE	GF-AAS	Cd(II), Pb(II)	Cd(II): 0.2–4.0 Pb(II): 2.0–50.0	Cd(II): 0.08 Pb(II): 0.41	Cd(II): 0.24 Pb(II): 1.3	Cd(II): 7.5	Milk	90.4–110	^68^
						Pb(II): 9.0			
DES-UAE	GF-AAS	Cu(II)	5–80	2.0	8.0	<10	tea samples	95–112	^68^
MAME	GF-AAS	Ni(II), Cu(II), Pb(II), Cd(II)	0.7–1.20	2.0–5.0	7.0–15.0	<4.54	marine bioindicators (mussels, polychaetes)	72–110	^70^
			0.1–1.50						
DES extraction	GF-AAS	Pb(II)	5–100	2.0	5.0	-	biological samples	91–110	^71^
CPE	TS-FF-AAS	Cd(II), Cu(II), Pb(II)	Cd(II): 2–10, Cu(II): 5–50, Pb(II): 5–50	Cd(II): 0.025, Cu(II): 0.38, Pb(II): 0.43	Cd(II): 0.083, Cu(II): 1.27, Pb(II): 1.44	Cd(II): 59, Cu(II): 25, Pb(II): 21	mineral water	94–113	^72^
SPE (PUF–APDC)	GF-AAS	Cd(II), Pb(II)	Cd(II): 0.06–3, Pb(II): 1.8–40	Cd(II): 0.02, Pb(II): 0.5	Cd(II): 0.06, Pb(II): 1.8	-	swimming pool water	82–105	^73^
MSPE	ETAAS	Cd(II), Cr(VI)	5–100	<0.3	<0.3	100	basil, pennyroyal, dill	58.63–99.03	^74^

Although several reported methods provide lower detection limits by employing highly sensitive instrumental techniques such as GF-AAS or ETAAS, the proposed method offers several distinctive advantages. Unlike conventional sorbent selection based on empirical screening, the sorbent used in this work was rationally designed through DFT calculations, enabling the selection of the most suitable anthraquinone derivative prior to synthesis. Furthermore, all critical extraction parameters were simultaneously optimized using central composite design (CCD), providing a statistically reliable multivariate optimization instead of the traditional one-factor-at-a-time approach. In addition, the analytical greenness of the proposed procedure was comprehensively evaluated using three complementary assessment tools (Eco-Scale, GAPI and AGREE), demonstrating its environmental sustainability.^69^

## Conclusion

In the present study, a novel ultrasound-assisted dispersive micro solid-phase extraction (UA-DMSPE) method coupled with flame atomic absorption spectrometry (FAAS) was successfully developed for the sensitive, reliable, low-cost, and environmentally friendly determination of Pb­(II) and Cd­(II) ions in rice and paddy soil samples. Three previously unreported anthraquinone-derived ligands were synthesized and comparatively evaluated through an integrated theoretical and experimental strategy. Density functional theory (DFT) calculations revealed that Derivative 2, exhibiting the lowest HOMO–LUMO energy gap (Δ*E* = 2.1284 eV) together with the most favorable electronic reactivity descriptors (η = 1.0642, σ = 347.8739, ω = 28.2323), possessed superior coordination potential toward Pb­(II) and Cd­(II) ions. This theoretical prediction was experimentally validated, and the Derivative 2-functionalized sorbent demonstrated excellent extraction performance, providing recoveries of approximately 95–99% for both analytes under optimized conditions. To achieve reliable extraction performance, the extraction procedure was systematically optimized through a combined OFAT–CCD/RSM strategy. Preliminary one-factor-at-a-time (OFAT) experiments were initially employed to establish suitable working intervals for the investigated variables, whereas CCD coupled with response surface methodology enabled simultaneous evaluation of both individual and interactive effects of experimental parameters on extraction efficiency. The developed quadratic model exhibited high statistical significance (*F* = 39.85, *p* < 0.0001) together with a nonsignificant lack-of-fit (*p* = 0.2669), confirming the robustness, adequacy, and predictive capability of the optimization model. Furthermore, validation experiments performed under predicted optimum conditions demonstrated close agreement between experimental and predicted responses, supporting the reliability of the developed method. Under optimum conditions, the proposed UA-DMSPE–FAAS method provided low detection limits of 0.19 and 0.28 μg L^–1^, wide linear working ranges (0.8–500 and 1.0–500 μg L^–1^), satisfactory precision (%RSD < 2.2), and excellent recoveries, indicating highly satisfactory analytical performance. Matrix effect investigations demonstrated only minor signal suppression, while satisfactory recoveries obtained from spiked rice and paddy soil samples confirmed the applicability, selectivity, and reliability of the developed method in complex matrices. The simultaneous assessment of rice and paddy soil samples further enabled a more comprehensive evaluation of the potential transfer of toxic metals from agricultural soils to edible plant systems, which is of particular importance considering the food safety and public health risks associated with Pb­(II) and Cd­(II) contamination in rice cultivation. Furthermore, the environmental sustainability of the developed analytical procedure was comprehensively confirmed through Analytical Eco-Scale, GAPI, and AGREE assessments, demonstrating strong compliance with green analytical chemistry principles, with an AGREE score of approximately 0.84. The solvent-free extraction procedure, low reagent consumption, minimal waste generation, and reduced acid usage collectively highlight the environmentally benign nature of the proposed method. In addition, the developed method offers operational simplicity, relatively short analysis time, low operational cost, and compatibility with widely accessible FAAS instrumentation, supporting its practical applicability for routine monitoring purposes. To the best of our knowledge, this study represents the first integrated analytical strategy combining newly synthesized anthraquinone-derived ligands, DFT-guided sorbent selection, OFAT-assisted CCD optimization, and comprehensive green analytical assessment for the simultaneous determination of Pb­(II) and Cd­(II) in rice and paddy soil matrices. Overall, the integration of a novel anthraquinone-based sorbent, theoretically supported ligand selection, statistically validated multivariate optimization, satisfactory matrix tolerance, and strong green analytical performance within a single analytical platform represents a significant contribution to the literature. The developed UA-DMSPE–FAAS method offers an effective, theoretically supported, practical, and environmentally friendly analytical strategy for the trace determination of Pb­(II) and Cd­(II) in environmentally and agriculturally important matrices.

## Supplementary Material



## Notes

^1^H NMR, ^13^C NMR, IR, and HR-MS spectra of the three anthraquinone derivatives; central composite design (CCD) experimental matrix and corresponding extraction recovery values for Pb­(II) and Cd­(II); detailed Analytical Eco-Scale scoring worksheet; detailed GAPI classification worksheet; complete AGREE scoring worksheet according to the 12 principles of Green Analytical Chemistry (PDF)
