# Supplementary material for: CCD-Optimized UA-DMSPE Combined with DFT Assisted Selection of a Novel Anthraquinone-Based Sorbent for Pb(II) and Cd(II) Determination in Rice and Paddy Soil
Source: ACS Omega. 2026 Aug 26;11(35):53127–55. doi: 10.1021/acsomega.6c05694 (PMC13564005; doi:10.1021/acsomega.6c05694)
Supplement: Supplementary file 1 [file ao6c05694_si_001.pdf]

SUPPLEMENTARY MATERIAL

CCD-Optimized UA-DMSPE Combined with DFT Assisted Selection of a Novel Anthraquinone-Based Sorbent for Pb(II) and Cd(II) Determination in Rice and Paddy Soil

Taşkın Mumcu*[a], Serkan Öncüoğlu[b], AkınMumcu[c], Ülkü Yılmaz[d]

[a]Department of Chemistry, Faculty of Sciences, Dokuz EylulUniversity, Merkez Campus, 35160 Buca-Izmir, TÜRKİYE

[b]Department of Chemistry, Faculty of Sciences, Dokuz EylulUniversity, Merkez Campus, 35160 Buca-Izmir, TÜRKİYE

[c]InonuUniversity, ScientificandTechnologicalResearch Center, Laboratory of NMR, Malatya 44280, TÜRKİYE

[d]Malatya Turgut Özal University, Faculty of Engineering and Natural Sciences, Department of Engineering Basic Sciences, Malatya 44900, TÜRKİYR

*Corresponding author: Taşkın Mumcu

**Content**
**Figure S1**. ^{1}H NMR spectrumacquired using a 600 MHz NMR spectrometerof Derivative 1.
**Figure S2**. ^{13}C NMR spectrumacquired using a 600 MHz NMR spectrometer of Derivative 1.
**Figure S3**. IR spectrum of Derivative1.
**Figure S4.**HR-MSspectrum of Derivative1.
**Figure S5**. ^{1}H NMR spectrumacquired using a 600 MHz NMR spectrometerof Derivative 2.
**Figure S6**. ^{13}C NMR spectrumacquired using a 600 MHz NMR spectrometer of Derivative 2.
**Figure S7**. IR spectrum of Derivative2.
**Figure S8.**HR-MSspectrum of Derivative2.
**Figure S9**. ^{1}H NMR spectrumacquired using a 600 MHz NMR spectrometerof Derivative 3.
**Figure S10**. ^{13}C NMR spectrumacquired using a 600 MHz NMR spectrometer of Derivative 3.
**Figure S11**. IR spectrum of Derivative3.
**Figure S12.**HR-MSspectrum of Derivative3.
**Table S1.** Central compositedesign (CCD) experimentalmatrixand corresponding extractionrecoveryvaluesfor Pb(II) andCd(II) ionsused in the optimisation of the UA-DMSPE procedure.
**Table S2.** Detailed Analytical Eco-Scale scoring worksheet for the optimized UA-DMSPE–FAAS procedure
**Table S3.** Detailed GAPI classification worksheet for the optimized UA-DMSPE–FAAS procedure
**Table S4.** Complete AGREE scoring worksheet according to the twelve principles of Green Analytical Chemistry

Spectra

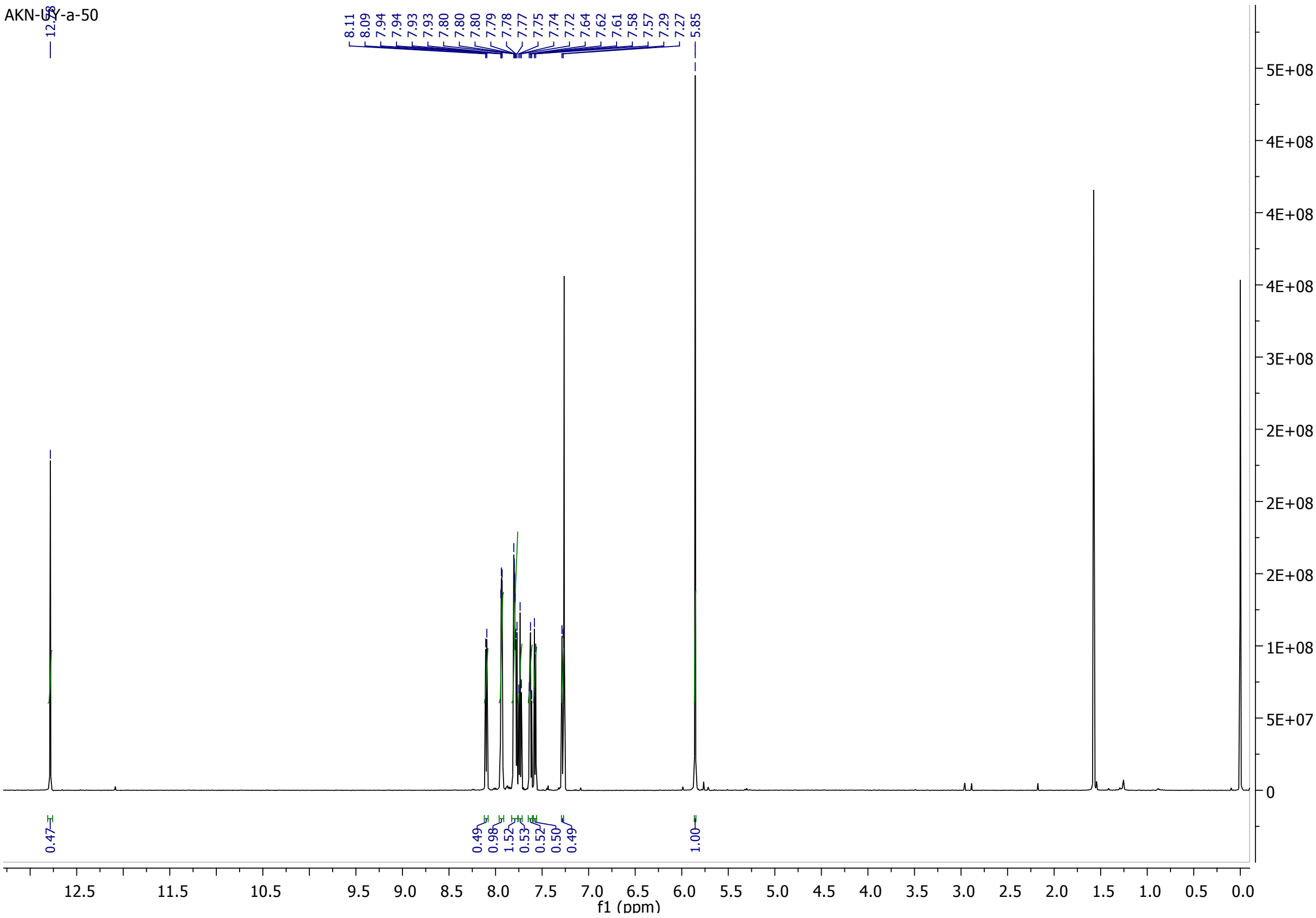

Figure S1. ^{1}H NMR spectrumacquired using a 600 MHz NMR spectrometerof Derivative **1**.

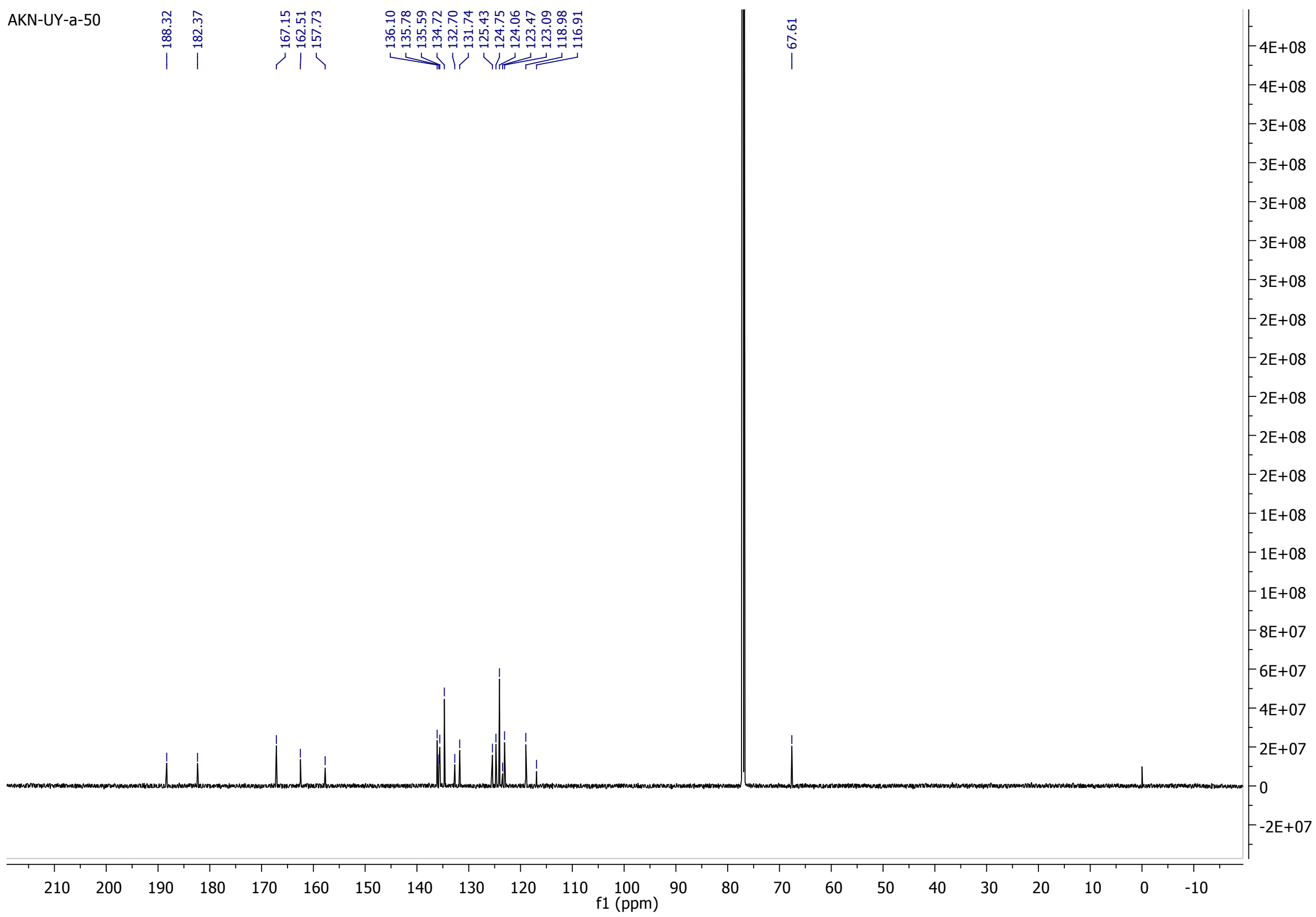

Figure S2. ^{13}C NMR spectrumacquired using a 600 MHz NMR spectrometer of Derivative **1**.

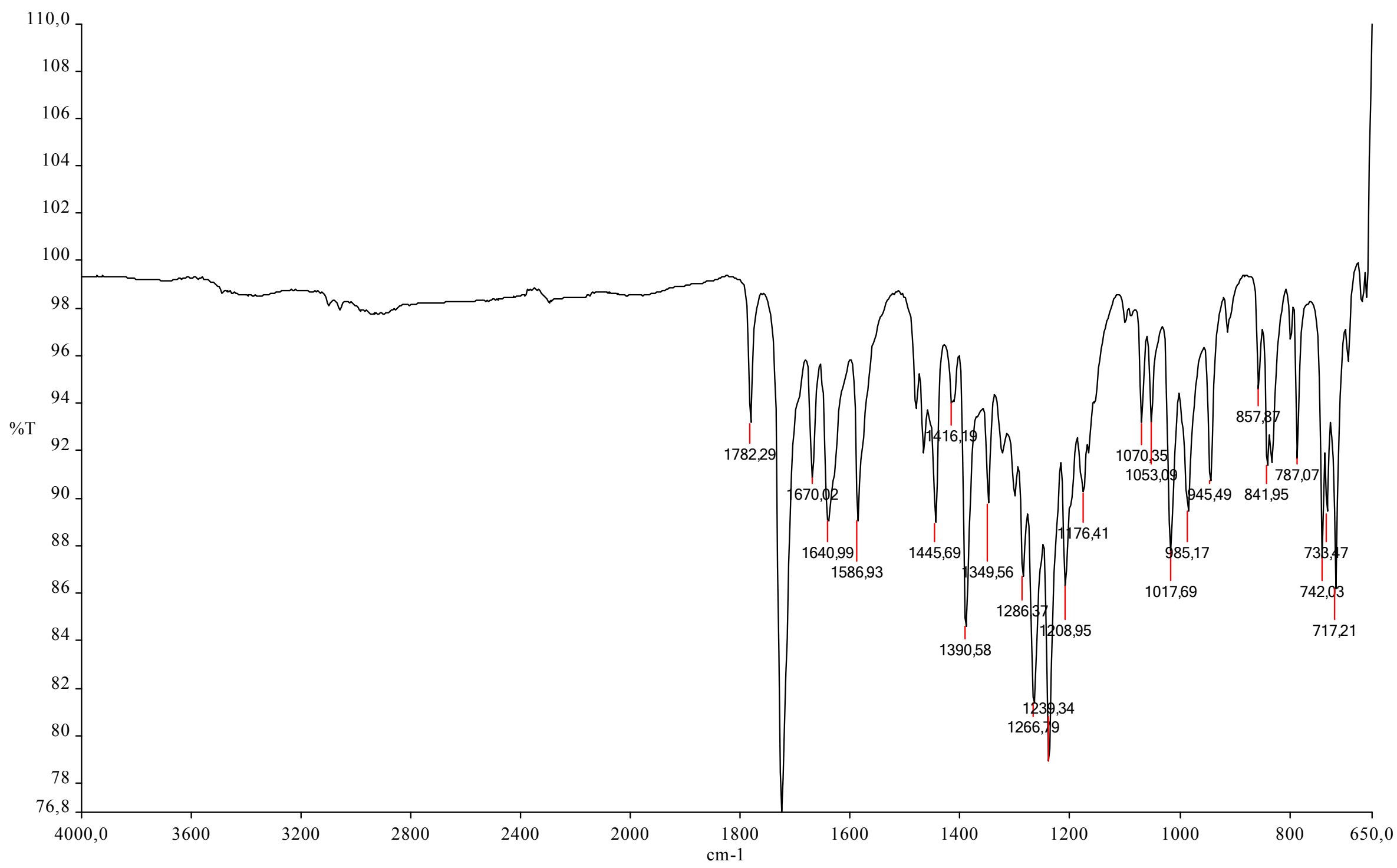

Figure S3.IR spectrum of Derivative **1**

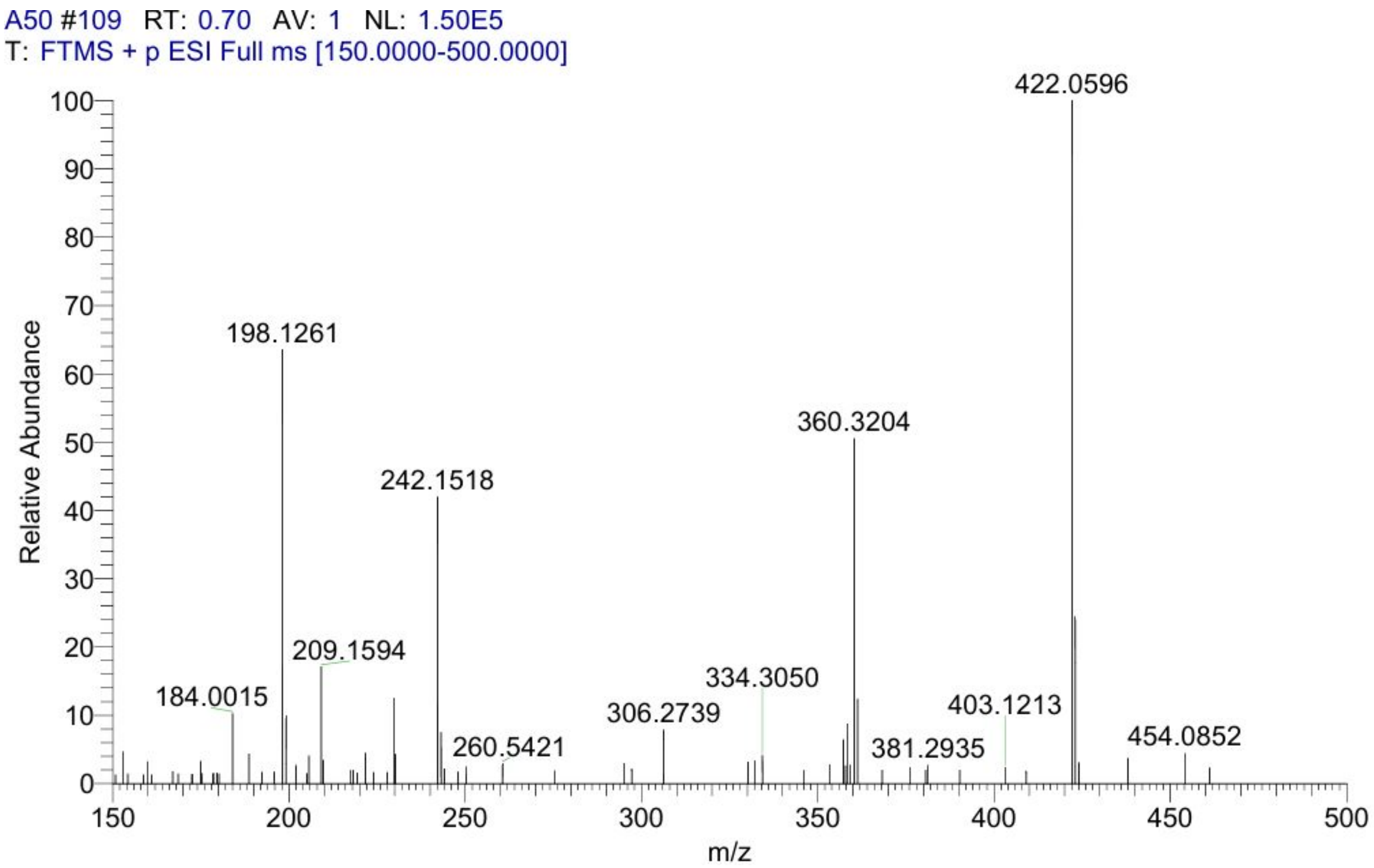

Figure S4.HR-MSspectrum of Derivative **1**

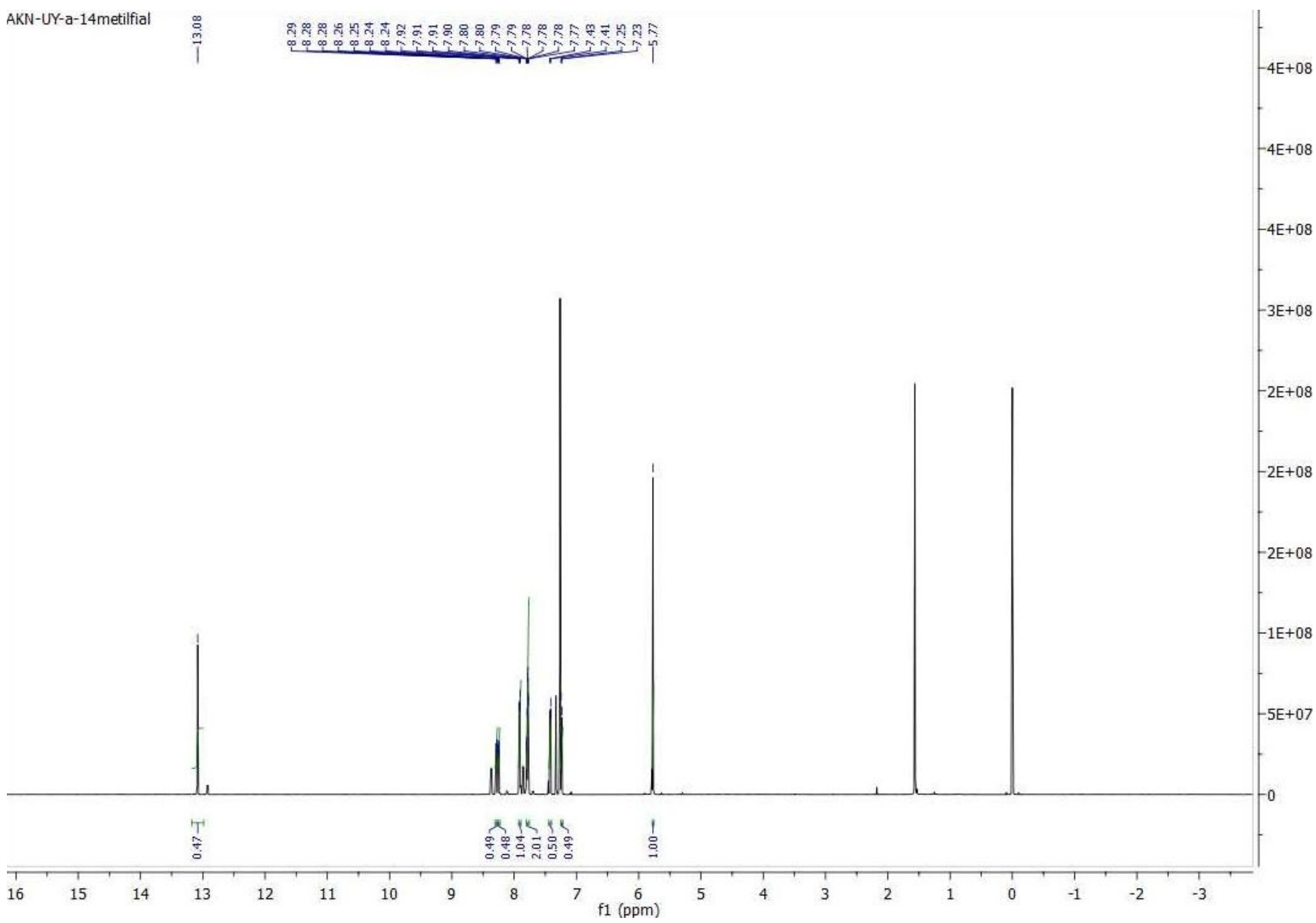

Figure S5. ^{1}H NMR spectrumacquired using a 600 MHz NMR spectrometerof Derivative **2**.

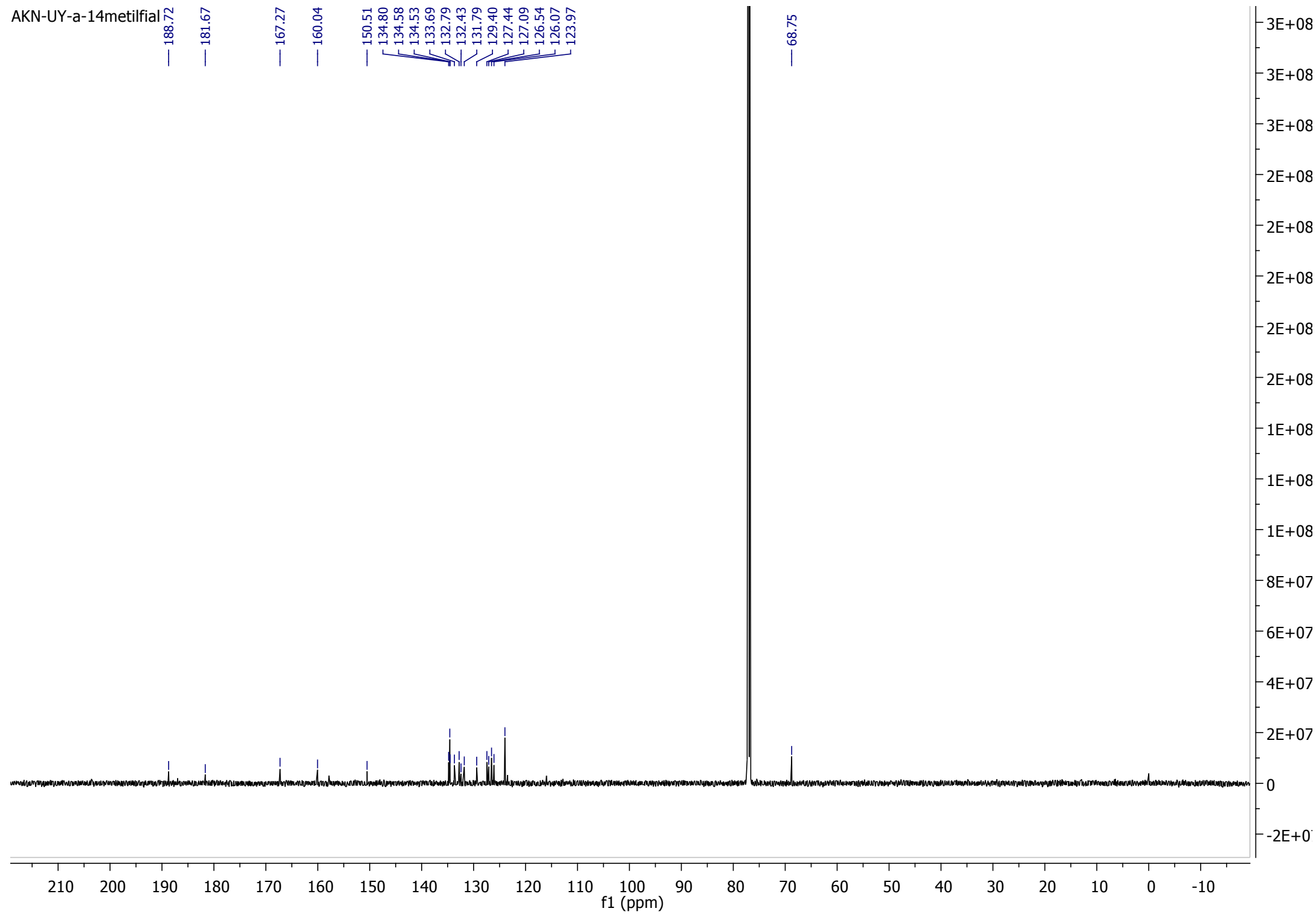

Figure S6. ^{13}C NMR spectrumacquired using a 600 MHz NMR spectrometer of Derivative **2**.

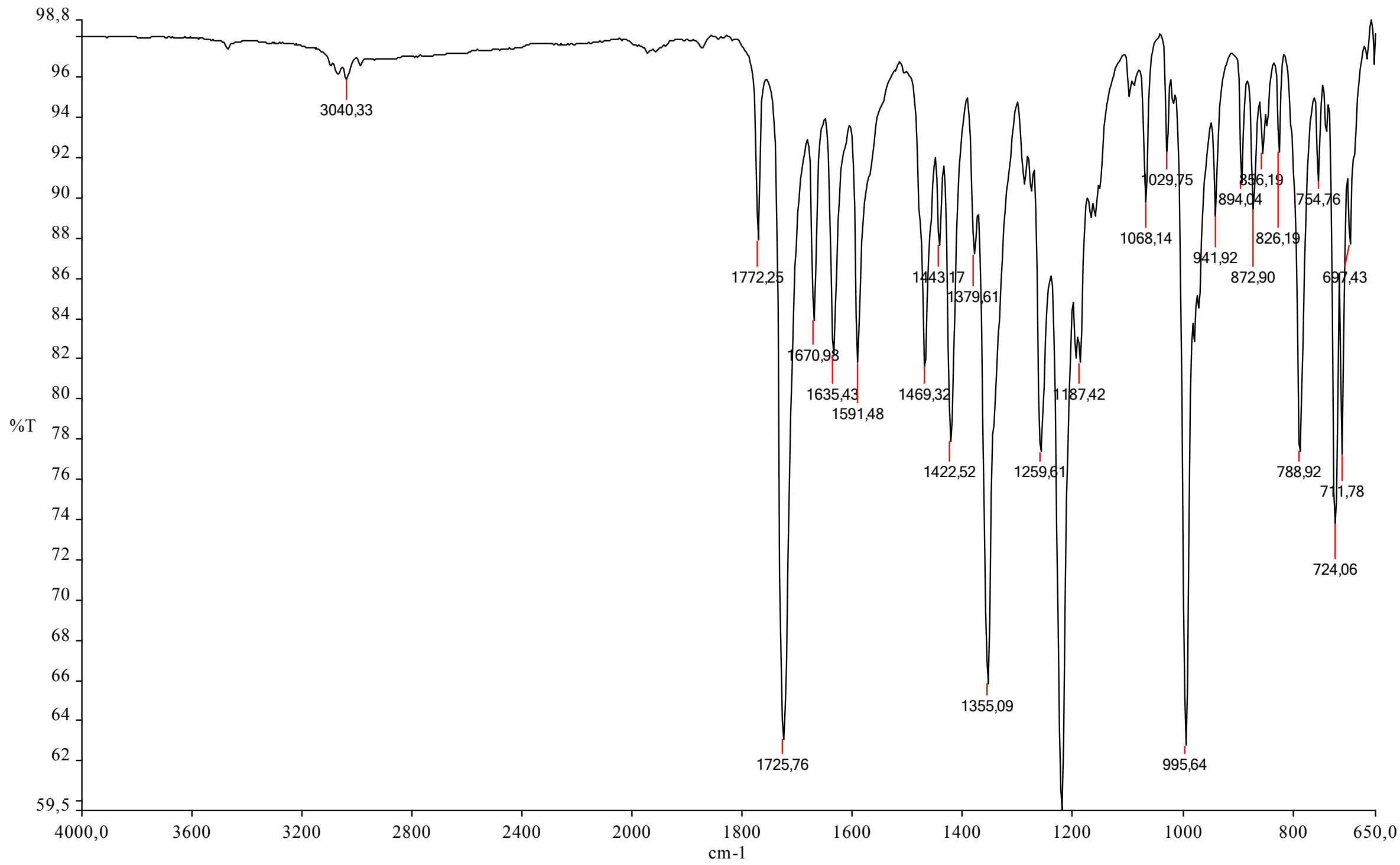

Figure S7. IR spectrum of Derivative **2**.

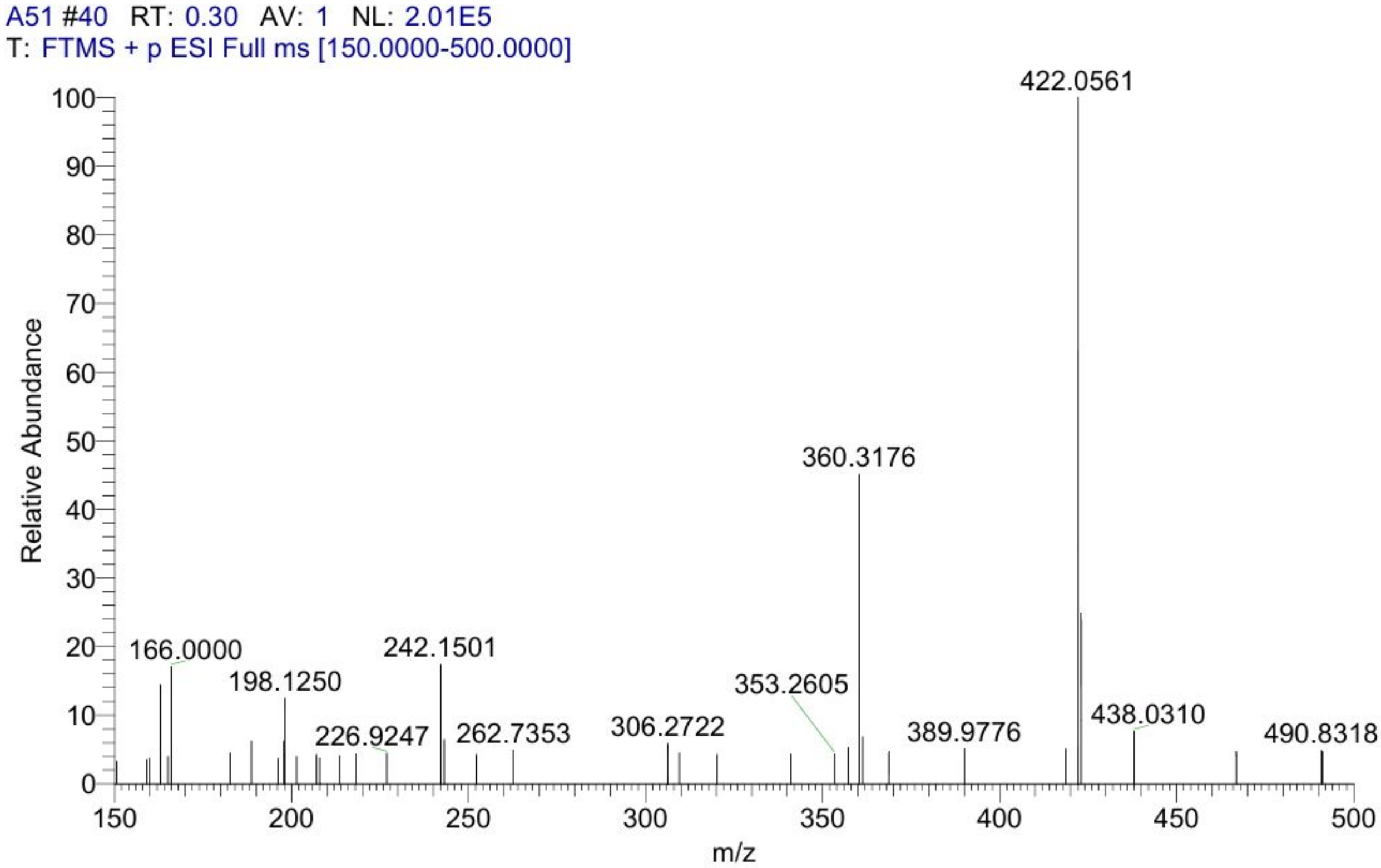

Figure S8.HR-MSspectrum of Derivative **2**.

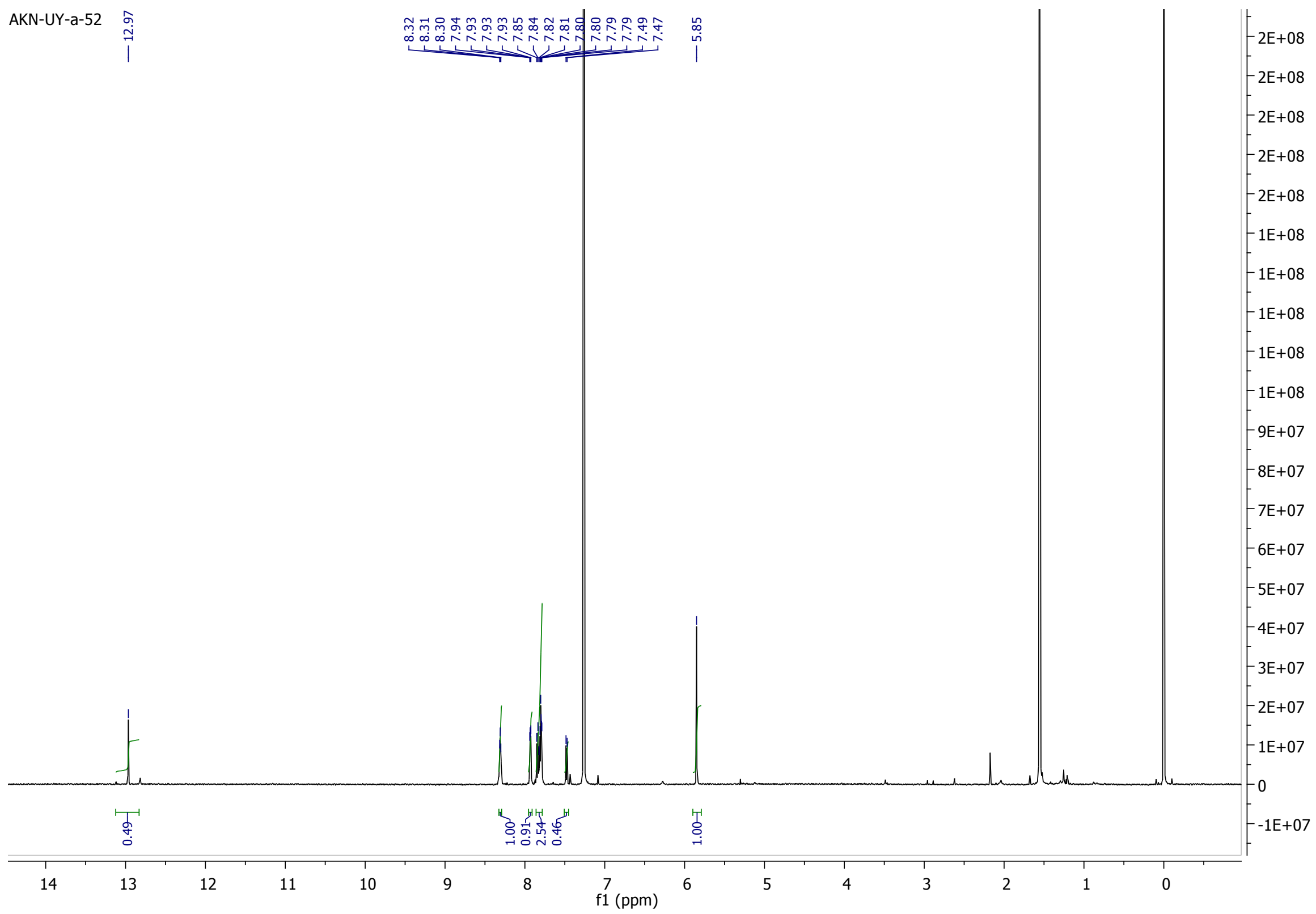

Figure S9. ^{1}H NMR spectrumacquired using a 600 MHz NMR spectrometerof Derivative **3**.

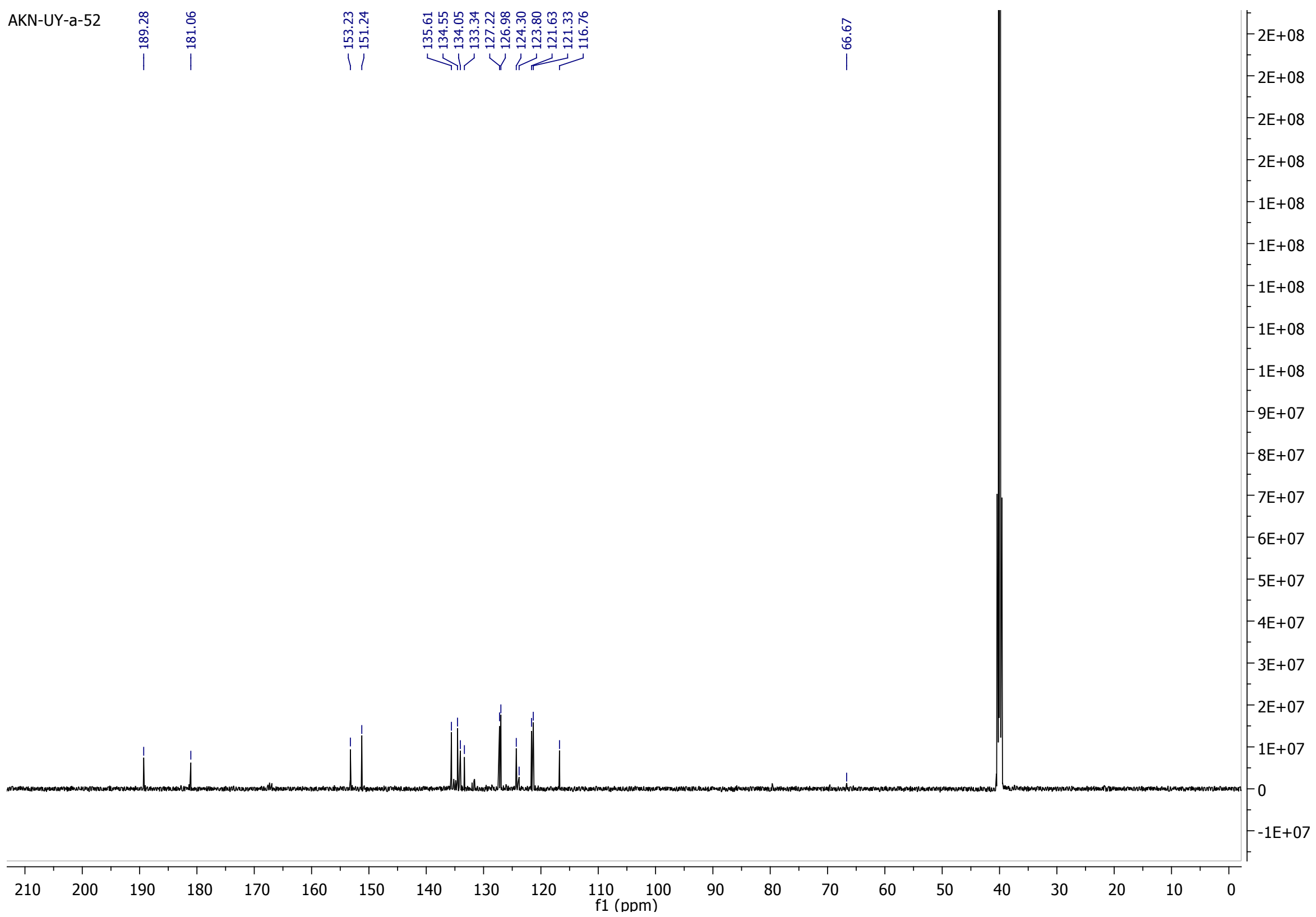

Figure S10. ^{13}C NMR spectrumacquired using a 600 MHz NMR spectrometer of Derivative **3**.

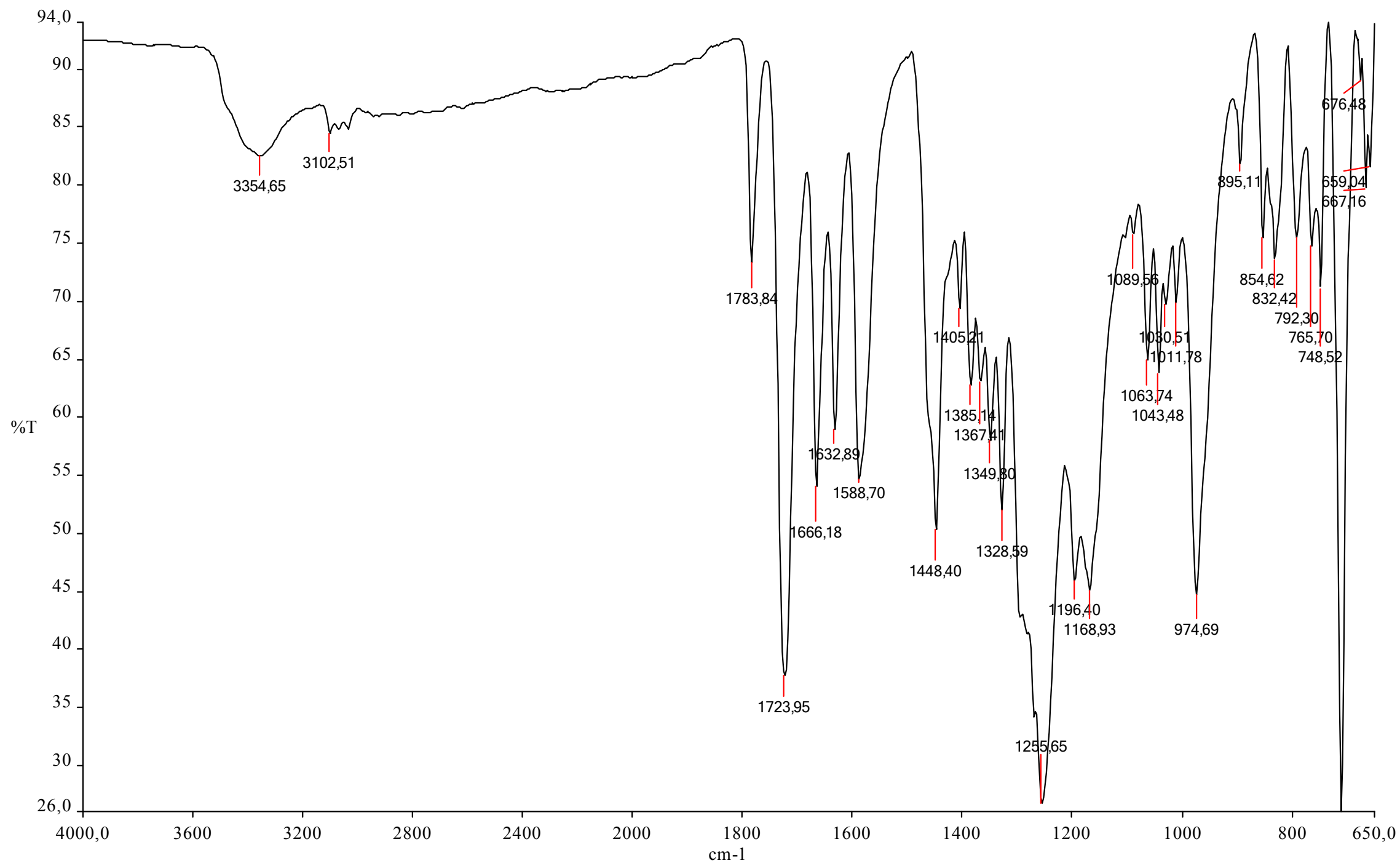

Figure S11. IR spectrum of Derivative **3**.

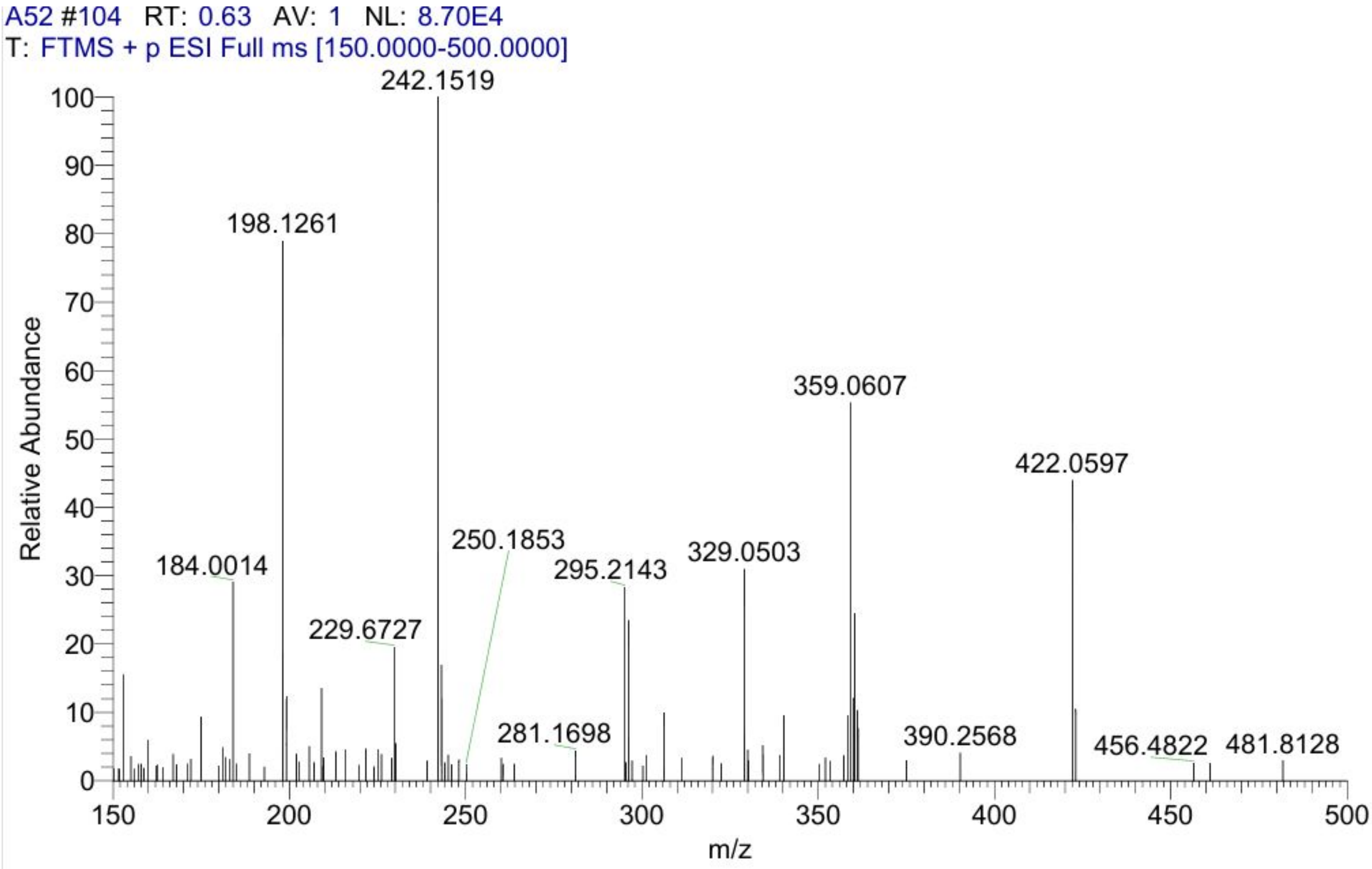

Figure S12.HR-MSspectrum of Derivative **3**.

Table S1. Central composite design (CCD) experimental matrixand corresponding extraction recovery values for Pb(II) andCd(II) ionsused in the optimisation of the UA-DMSPE procedure.

		Factor 1	Factor 2	Factor 3	Factor 4	Factor 5	Factor 6	Response 1
Std	Run	A:Ligand Concentration	B:Eluent Volume	C:pH	D:Sorbent Amount	E:Extraction Time	F:Sample Volume	Recovery
		mol L^{-1}	mL		mg	min	mL	%
7	1	1E-05	2,5	9	10	5	2,5	20,7
64	2	0,0005	2,5	9	50	30	20	31,6
52	3	0,0005	2,5	3	10	30	20	20,8
26	4	0,0005	0,2	3	50	30	2,5	20,3
77	5	0,000255	1,35	6	30	17,5	11,25	100
37	6	1E-05	0,2	9	10	5	20	21,2
53	7	1E-05	0,2	9	10	30	20	22,3
30	8	0,0005	0,2	9	50	30	2,5	23,7
44	9	0,0005	2,5	3	50	5	20	21,4
6	10	0,0005	0,2	9	10	5	2,5	22,8
48	11	0,0005	2,5	9	50	5	20	29,6
69	12	0,000255	1,35	3	30	17,5	11,25	78,7
17	13	1E-05	0,2	3	10	30	2,5	26,2
42	14	0,0005	0,2	3	50	5	20	19,6

80	15	0,000255	1,35	6	30	17,5	11,25	92,3
68	16	0,000255	2,5	6	30	17,5	11,25	84,7
34	17	0,0005	0,2	3	10	5	20	22,2
86	18	0,000255	1,35	6	30	17,5	11,25	92,8
74	19	0,000255	1,35	6	30	30	11,25	84,9
13	20	1E-05	0,2	9	50	5	2,5	23,8
3	21	1E-05	2,5	3	10	5	2,5	20,6
63	22	1E-05	2,5	9	50	30	20	27,7
38	23	0,0005	0,2	9	10	5	20	20,6
60	24	0,0005	2,5	3	50	30	20	23,1
28	25	0,0005	2,5	3	50	30	2,5	21,6
27	26	1E-05	2,5	3	50	30	2,5	21,1
56	27	0,0005	2,5	9	10	30	20	22,6
57	28	1E-05	0,2	3	50	30	20	22,3
2	29	0,0005	0,2	3	10	5	2,5	26
71	30	0,000255	1,35	6	5	17,5	11,25	82,1
47	31	1E-05	2,5	9	50	5	20	26,5
15	32	1E-05	2,5	9	50	5	2,5	24,6
16	33	0,0005	2,5	9	50	5	2,5	26,4
45	34	1E-05	0,2	9	50	5	20	25,4
31	35	1E-05	2,5	9	50	30	2,5	26,2
67	36	0,000255	0,5	6	30	17,5	11,25	89,6
62	37	0,0005	0,2	9	50	30	20	24,1
1	38	1E-05	0,2	3	10	5	2,5	26,5
85	39	0,000255	1,35	6	30	17,5	11,25	100
18	40	0,0005	0,2	3	10	30	2,5	25,8
78	41	0,000255	1,35	6	30	17,5	11,25	82,2
50	42	0,0005	0,2	3	10	30	20	23,4
43	43	1E-05	2,5	3	50	5	20	21,9
8	44	0,0005	2,5	9	10	5	2,5	22,6
41	45	1E-05	0,2	3	50	5	20	21,1
5	46	1E-05	0,2	9	10	5	2,5	21,8
51	47	1E-05	2,5	3	10	30	20	20,4
81	48	0,000255	1,35	6	30	17,5	11,25	100
66	49	0,0005	1,35	6	30	17,5	11,25	91,4
84	50	0,000255	1,35	6	30	17,5	11,25	97,9
58	51	0,0005	0,2	3	50	30	20	21,1
11	52	1E-05	2,5	3	50	5	2,5	21,5
24	53	0,0005	2,5	9	10	30	2,5	23,3
19	54	1E-05	2,5	3	10	30	2,5	21,6
10	55	0,0005	0,2	3	50	5	2,5	20,6
83	56	0,000255	1,35	6	30	17,5	11,25	94,5
25	57	1E-05	0,2	3	50	30	2,5	22,6
14	58	0,0005	0,2	9	50	5	2,5	21,7
22	59	0,0005	0,2	9	10	30	2,5	22,8
36	60	0,0005	2,5	3	10	5	20	21,8
76	61	0,000255	1,35	6	30	17,5	20	84,9
32	62	0,0005	2,5	9	50	30	2,5	28,6

61	63	1E-05	0,2	9	50	30	20	24,6
55	64	1E-05	2,5	9	10	30	20	22,8
54	65	0,0005	0,2	9	10	30	20	23,4
82	66	0,000255	1,35	6	30	17,5	11,25	100
12	67	0,0005	2,5	3	50	5	2,5	23,9
35	68	1E-05	2,5	3	10	5	20	19,1
75	69	0,000255	1,35	6	30	17,5	2,5	83,4
33	70	1E-05	0,2	3	10	5	20	24,2
73	71	0,000255	1,35	6	30	5	11,25	85
46	72	0,0005	0,2	9	50	5	20	24,2
72	73	0,000255	1,35	6	50	17,5	11,25	82,9
21	74	1E-05	0,2	9	10	30	2,5	20,3
29	75	1E-05	0,2	9	50	30	2,5	23,2
79	76	0,000255	1,35	6	30	17,5	11,25	83,6
39	77	1E-05	2,5	9	10	5	20	21,9
65	78	1E-05	1,35	6	30	17,5	11,25	94,1
59	79	1E-05	2,5	3	50	30	20	22,5
70	80	0,000255	1,35	9	30	17,5	11,25	80,6
9	81	1E-05	0,2	3	50	5	2,5	21,7
49	82	1E-05	0,2	3	10	30	20	24,3
4	83	0,0005	2,5	3	10	5	2,5	21,9
23	84	1E-05	2,5	9	10	30	2,5	21,3
20	85	0,0005	2,5	3	10	30	2,5	21,7
40	86	0,0005	2,5	9	10	5	20	22,8

Detailed greenness-assessment worksheets. To improve transparency and reproducibility, complete scoring worksheets for the Analytical Eco-Scale, GAPI, and AGREE evaluations are provided in Tables S2–S4. The assessment scope was restricted to the optimized UA-DMSPE–FAAS analytical procedure, comprising a 10.0 mL sample extract, pH adjustment to 6.0, 30 mg of Derivative 2–SiO_2 sorbent, 20 min ultrasound-assisted extraction, two centrifugation steps, elution with 1.0 mL of 0.5 mol L^{-1} HNO_3, and FAAS determination. The basis for every penalty point, numerical score, and GAPI color assignment is explicitly stated. The Analytical Eco-Scale score was calculated as 100 − 12 = 88. The GAPI profile contained seven green and eight yellow fields, with no red field. The AGREE assessment yielded an overall score of approximately 0.84.

Table S2. Detailed Analytical Eco-Scale scoring worksheet for the optimized UA-DMSPE–FAAS procedure

Assessment item	Experimental condition	Penalty/reward standard applied	Assigned penalty points	Calculation and justification
Eluent: nitric acid	1.0 mL of 0.5 mol L^{-1} HNO_3 per analytical run.	A corrosive mineral acid is used, but only as a dilute aqueous solution and at a micro-volume.	3	The acid is necessary for quantitative desorption. The low concentration and 1.0 mL volume limit the penalty.
pH-adjustment reagents	Dilute HNO_3 and NaOH added dropwise to obtain pH 6.0.	Corrosive acid/base reagents used in very small quantities.	2	Small, non-bulk additions are required only for pH adjustment. Reagent-hazard subtotal = 3 + 2 = 5.
Organic extraction solvent	No organic solvent is used during the optimized UA-DMSPE analytical procedure.	Absence of organic extraction solvent receives no penalty.	0	The extraction and elution media are aqueous.
Sorbent consumption	30 mg Derivative 2–SiO_2 per run.	Micro-scale solid-reagent consumption; no separate penalty included in the reported score.	0	Only 0.030 g sorbent is used per extraction.
Ultrasound-assisted extraction	20 min at room temperature.	Limited electrical-energy input without external heating.	1	Short sonication at ambient temperature.
Centrifugation	5000 rpm for 10 min after adsorption and again after elution (2 × 10 min).	Two electrically powered separation steps.	2	The procedure requires two centrifugation cycles.
FAAS determination	Sequential measurement of Pb(II) and Cd(II) using air–acetylene FAAS.	Instrumental energy and fuel-gas consumption.	2	Energy subtotal = ultrasound (1) + centrifugation (2) + FAAS (2) = 5.
Waste generation	Approximately 10.0 mL aqueous supernatant plus 1.0 mL acidic eluate; total ≈11 mL per run.	Low-volume aqueous waste; no organic-solvent waste.	2	Waste must be collected as heavy-metal-containing laboratory waste; waste subtotal = 2.
Occupational safety	Routine PPE, fume hood for acid handling, and standard FAAS flame precautions.	No additional penalty beyond reagent and instrumental hazards already counted.	0	No volatile toxic extraction solvent or unusual process-specific hazard is present.
TOTAL PENALTY POINTS			12	5 (reagents) + 5 (energy/instrumentation) + 2 (waste) + 0 (additional occupational hazard) = 12.
FINAL ECO-SCALE SCORE			88	100 − 12 = 88.
Classification:				Excellent green analysis (Eco-Scale >75).

Table S3. Detailed GAPI classification worksheet for the optimized UA-DMSPE–FAAS procedure

GAPI field	Method-specific input	Decision standard	Assigned color	Reason for assignment
1. Sampling approach	Rice and paddy-soil samples are collected externally and transported to the laboratory.	In-situ/on-line sampling is greener; external off-line sampling receives a moderate classification.	Yellow	The determination is not performed directly at the sampling site.
2. Sample preservation	No chemical preservative is added; samples are dried and stored.	No chemical preservation or only simple physical stabilization receives a favorable classification.	Green	No preservation reagent is consumed.
3. Sample transport	Samples are transported to the laboratory.	Laboratory transport has a greater footprint than in-situ determination.	Yellow	Transport is necessary.
4. Sample storage	Dried solid samples are stored under ordinary laboratory conditions.	No refrigeration and no stabilizing chemicals are favorable.	Green	Storage demands are limited.
5. Sample preparation	The evaluated analytical stage uses the previously prepared 10.0 mL sample extract, followed by pH adjustment and UA-DMSPE.	A short microextraction procedure is favorable, although more than one handling step is required.	Yellow	Adsorption, centrifugation, elution, and a second centrifugation are required.
6. Extraction scale	10.0 mL sample extract and 30 mg sorbent.	Micro-scale sample and sorbent consumption are favorable.	Green	The working volume and sorbent mass are low.
7. Reagent amount	1.0 mL 0.5 mol L^{-1} HNO_3 and dropwise dilute HNO_3/NaOH.	Low-volume reagent use is favorable.	Green	Only small aqueous reagent volumes are consumed.
8. Reagent hazard	Dilute HNO_3 and NaOH are corrosive.	Use of corrosive reagents produces a moderate hazard classification even at low volume.	Yellow	PPE and controlled handling are required.
9. Organic-solvent use	No organic extraction solvent is used.	Solvent-free aqueous extraction receives the most favorable classification.	Green	No volatile or flammable extraction solvent is generated as waste.
10. Additional treatment	20 min ultrasonication and two 10 min centrifugation steps.	Multiple powered operations create a moderate impact.	Yellow	Electrical energy and equipment are required.
11. Automation/miniaturization	The method is miniaturized but manually operated.	Miniaturization is favorable; lack of automation prevents the most favorable overall classification.	Yellow	Manual transfers and decantation are necessary.
12. Detection technique	Sequential Pb and Cd determination by FAAS.	Routine instrumental analysis with flame-gas and electrical-energy requirements gives a moderate classification.	Yellow	FAAS is accessible but consumes fuel gas and energy.
13. Occupational safety	Dilute corrosive reagents and an air–acetylene flame are used.	Routine but non-zero operator hazard.	Yellow	Fume-hood, PPE, and flame-safety procedures are required.
14. Waste amount	Total liquid waste ≈11 mL per run.	Waste below approximately 15 mL per run is relatively favorable.	Green	The volume is low and no organic-solvent waste is produced.
15. Waste management	Aqueous residue may contain Pb and Cd and is collected as hazardous heavy-metal waste.	Collection and controlled disposal are necessary; direct disposal is not acceptable.	Yellow	The volume is low, but the presence of toxic metals requires regulated disposal.

Table S4. Complete AGREE scoring worksheet according to the twelve principles of Green Analytical Chemistry

No.	Green Analytical Chemistry principle	Method-specific input / evidence	Assigned score (0–1)	Detailed scoring justification
1	Direct analytical techniques preferred	Trace Pb(II) and Cd(II) require UA-DMSPE preconcentration before FAAS.	0.80	The method is not direct because an extraction/preconcentration stage precedes measurement; however, the preparation is relatively simple.
2	Minimal sample size and minimum number of operations	10.0 mL sample extract; adsorption, separation, elution, and measurement.	0.95	The analytical sample volume is small and the operation count is limited compared with conventional SPE/LLE.
3	In-situ measurements	Laboratory-based FAAS; no field-portable or in-situ measurement.	0.70	The analytical system requires transport to and operation in a laboratory.
4	Integration and minimization of sample preparation	Only pH adjustment is required immediately before UA-DMSPE; extraction and preconcentration are combined.	0.85	The extraction stage is compact, but manual centrifugation and elution remain necessary.
5	Automation and miniaturization	Miniaturized 10 mL batch procedure, manually operated.	0.70	Miniaturization is achieved, but automation and on-line integration are absent.
6	Avoidance of derivatization	No analyte derivatization is required.	1.00	Pb(II) and Cd(II) are retained directly by the functionalized sorbent.
7	Low reagent and solvent consumption	30 mg sorbent; 1.0 mL 0.5 mol L^{-1} HNO_3; dropwise dilute acid/base; no organic extraction solvent.	0.95	Reagent volumes and sorbent mass are low and the extraction is solvent-free.
8	Use of non-hazardous reagents	Predominantly aqueous media; dilute HNO_3 and NaOH are used.	0.85	The amount is low, but the corrosive character of acid/base reagents prevents a maximum score.
9	Energy efficiency	20 min ultrasonication, two centrifugation cycles, and FAAS.	0.75	Room-temperature extraction is favorable, but powered equipment and flame operation are required.
10	Waste minimization and proper waste management	≈11 mL aqueous waste per run; no organic-solvent waste; Pb/Cd-containing waste is collected separately.	0.90	The volume is low; however, toxic-metal content necessitates controlled hazardous-waste disposal.
11	Multi-analyte / multi-parameter capability	The same extraction treats Pb(II) and Cd(II), but FAAS measurements are sequential.	0.75	Two analytes are covered by one preparation procedure, but the detector is not simultaneous multi-element.
12	Operator safety and ergonomic design	Routine PPE, fume hood, and FAAS flame precautions; no toxic volatile extraction solvent.	0.95	Operator exposure is limited, although corrosive reagents and an open flame are present.
